# Solid-state molecular organometallic chemistry. Single-crystal to single-crystal reactivity and catalysis with light hydrocarbon substrates†
†Electronic supplementary information (ESI) available: Full details of experimental details, spectroscopic and other analytical data, X-ray crystallography, catalytic conditions, and computational studies. CCDC 1539832–1539836. For ESI and crystallographic data in CIF or other electronic format see DOI: 10.1039/c7sc01491k


**DOI:** 10.1039/c7sc01491k

**Published:** 2017-07-06

**Authors:** F. Mark Chadwick, Alasdair I. McKay, Antonio J. Martinez-Martinez, Nicholas H. Rees, Tobias Krämer, Stuart A. Macgregor, Andrew S. Weller

**Affiliations:** a Department of Chemistry , Chemistry Research Laboratories , University of Oxford , OX1 3TA , UK . Email: andrew.weller@chem.ox.ac.uk; b Institute of Chemical Sciences , Heriot Watt University , Edinburgh , EH14 4AS , UK . Email: S.A.Macgregor@hw.ac.uk

## Abstract

Single-crystal to single-crystal solid/gas reactivity and catalysis starting from the precursor sigma-alkane complex **[Rh(Cy_2_PCH_2_CH_2_PCy_2_)(η^2^η^2^-NBA)][BAr^F^_4_]** (NBA = norbornane; Ar^F^ = 3,5-(CF_3_)_2_C_6_H_3_) is reported. By adding ethene, propene and 1-butene to this precursor in solid/gas reactions the resulting alkene complexes **[Rh(Cy_2_PCH_2_CH_2_PCy_2_)(alkene)*_x_*][BAr^F^_4_]** are formed. The ethene (*x* = 2) complex, **[Rh(Cy_2_PCH_2_CH_2_PCy_2_)(ethene)_2_][BAr^F^_4_]-Oct**, has been characterized in the solid-state (single-crystal X-ray diffraction) and by solution and solid-state NMR spectroscopy. Rapid, low temperature recrystallization using solution methods results in a different crystalline modification, **[Rh(Cy_2_PCH_2_CH_2_PCy_2_)(ethene)_2_][BAr^F^_4_]-Hex**, that has a hexagonal microporous structure (*P*6_3_22). The propene complex (*x* = 1) **[Rh(Cy_2_PCH_2_CH_2_PCy_2_)(propene)][BAr^F^_4_]** is characterized as having a π-bound alkene with a supporting γ-agostic Rh···H_3_C interaction at low temperature by single-crystal X-ray diffraction, variable temperature solution and solid-state NMR spectroscopy, as well as periodic density functional theory (DFT) calculations. A fluxional process occurs in both the solid-state and solution that is proposed to proceed *via* a tautomeric allyl-hydride. Gas/solid catalytic isomerization of d_3_-propene, H_2_C=CHCD_3_, using **[Rh(Cy_2_PCH_2_CH_2_PCy_2_)(η^2^η^2^-NBA)][BAr^F^_4_]** scrambles the D-label into all possible positions of the propene, as shown by isotopic perturbation of equilibrium measurements for the agostic interaction. Periodic DFT calculations show a low barrier to H/D exchange (10.9 kcal mol^–1^, PBE-D3 level), and GIPAW chemical shift calculations guide the assignment of the experimental data. When synthesized using solution routes a bis-propene complex, **[Rh(Cy_2_PCH_2_CH_2_PCy_2_)(propene)_2_][BAr^F^_4_]**, is formed. **[Rh(Cy_2_PCH_2_CH_2_PCy_2_)(butene)][BAr^F^_4_]** (*x* = 1) is characterized as having 2-butene bound as the *cis*-isomer and a single Rh···H_3_C agostic interaction. In the solid-state two low-energy fluxional processes are proposed. The first is a simple libration of the 2-butene that exchanges the agostic interaction, and the second is a butene isomerization process that proceeds *via* an allyl-hydride intermediate with a low computed barrier of 14.5 kcal mol^–1^. **[Rh(Cy_2_PCH_2_CH_2_PCy_2_)(η^2^η^2^-NBA)][BAr^F^_4_]** and the polymorphs of **[Rh(Cy_2_PCH_2_CH_2_PCy_2_)(ethene)_2_][BAr^F^_4_]** are shown to be effective in solid-state molecular organometallic catalysis (SMOM-Cat) for the isomerization of 1-butene to a mixture of *cis*- and *trans*-2-butene at 298 K and 1 atm, and studies suggest that catalysis is likely dominated by surface-active species. **[Rh(Cy_2_PCH_2_CH_2_PCy_2_)(η^2^η^2^-NBA)][BAr^F^_4_]** is also shown to catalyze the transfer dehydrogenation of butane to 2-butene at 298 K using ethene as the sacrificial acceptor.

## Abstract 2

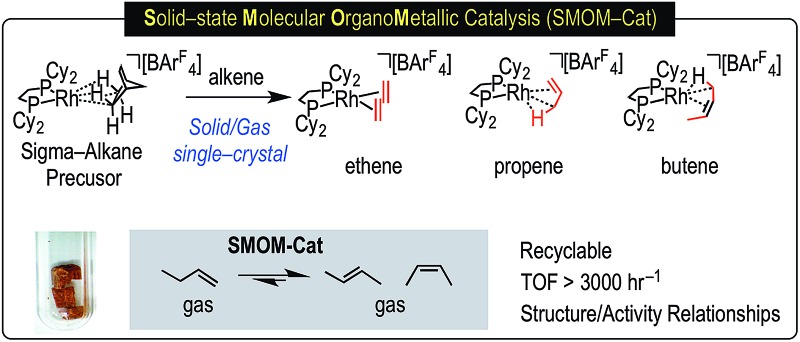
Solid-state molecular organometallic catalysis (SMOM-cat): synthetic routes, unique structural motifs, mobility in the solid-state and very active gas/solid isomerization catalysts.

## 1. Introduction

**Scheme 1:**
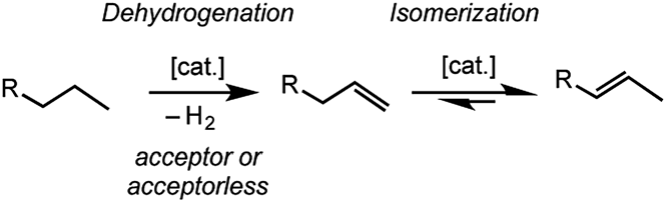
Dehydrogenation/isomerization of simple, light, alkanes (R = alkyl).

The transition metal promoted isomerization of alkenes is an atom efficient process that has many applications in industry and fine-chemicals synthesis;^1^–^3^ such as the Shell Higher Olefin Process,^4^ “on purpose” olefin conversion technologies that produce propene from butene/ethene mixtures,^5^–^8^ and the isomerization of functionalized alkenes.^9^ Homogeneous processes are well-studied for a wide range of transition metal catalysts^1^,^9^–^11^ and commonly, although by no means exclusively, use catalysts based upon later transition metals such as Co,^12^ Ni,^13^,^14^ Ru,^15^–^17^ Rh,^18^–^20^ Pd,^21^ and Ir,^22^–^24^ which operate at relatively low temperatures, sometimes at room temperature.^17^,^19^–^21^,^25^–^27^ Process based upon heterogeneous catalysts or surface organometallic chemistry (SOMC) are also well established.^28^,^29^ Alkene isomerization also plays a key role in alkane dehydrogenation,^30^ and subsequent tandem upgrading processes such as metathesis^31^ or dimerization,^32^,^33^ where the kinetic product of dehydrogenation is a terminal alkene that can then undergo isomerization (Scheme 1).^34^

The dehydrogenation of light alkanes such as butane and pentane, and their subsequent isomerization is particularly interesting, as while these alkanes are unsuitable as transportation fuels or feedstock chemicals, their corresponding alkenes have myriad uses.^32^,^33^,^35^ The discovery of abundant sources of light alkanes in shale and offshore gas fields provides additional motivation to study their conversion into fuels and commodity chemicals.^36^ As light alkanes are gaseous at, or close to, room temperature and pressure the opportunity for solid/gas catalytic processes under these conditions is presented. Such conditions are also attractive due to the physical separation of catalyst and substrates/products that they offer, as well as opportunities to reduce thermally-induced catalyst decomposition processes.

Although heterogeneous solid/gas systems for alkane dehydrogenation and alkene isomerization are well known,^29^,^37^–^40^ they often require high temperatures for their operation which lead to reductions in selectivity as well as catalyst deactivation through processes such as coking. As is often the case^41^,^42^ well-defined supported or molecular systems can offer lower barriers, albeit still having to overcome the endergonic dehydrogenation reaction when run without a sacrificial acceptor.^43^,^44^ As far as we are aware there are only a handful of examples of purely molecular, *i.e.* not supported, solid-phase catalysts for alkane dehydrogenation or alkene isomerization. The Ir-pincer catalysts, such as Ir(PCP^iPr^)(C_2_H_4_) [PCP^iPr^ = κ^3^-C_6_H_3_-2,6-(CH_2_P^i^Pr_2_)_2_], recently reported by Goldman and co-workers, promote the transfer dehydrogenation, and subsequent double-bond isomerization, of butane, pentane and octane using acceptors such as *tert*-butylethene, ethene or propene.^45^,^46^ These operate at temperatures of 200 °C or above in sealed-tube conditions in which all the alkane is expected to be in the gas phase, and can actually outperform homogeneous systems in terms of activity. Experimental evidence points towards a presumed molecular species as the active catalyst, although the precise details have not been disclosed. Siedle & Newmark reported the room temperature solid/gas isomerization of simple alkenes using iridium or rhodium phosphine cations partnered with Keggin-type trianions, such as [Ir(H)_2_(PPh_3_)_2_]_3_[PW_12_O_40_],^47^–^49^ however the precise molecular structure of the catalyst was not determined.

**Scheme 2:**
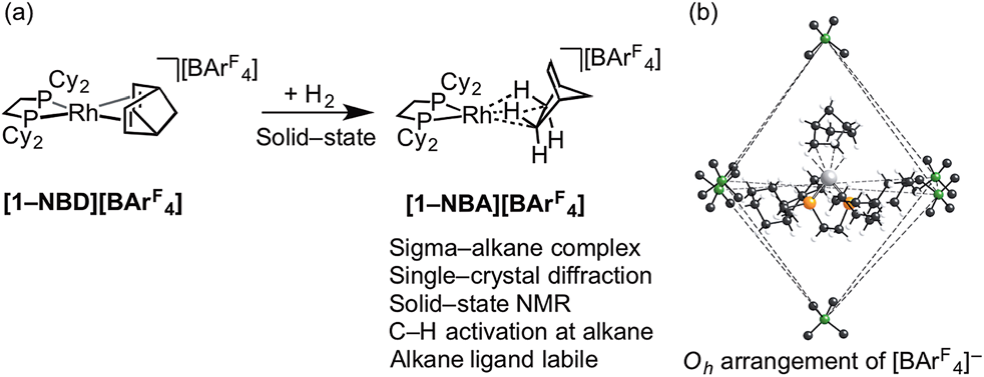
(a) Synthesis of sigma-alkane complex **[1-NBA][BAr^F^_4_]** by solid/gas reactivity; (b) *O*_h_ arrangement of **[BAr^F^_4_]** anions in the solid-state that encapsulate cation (Ar^F^ groups removed).

We have recently reported the synthesis, using single-crystal to single-crystal solid/gas techniques,^41^,^50^–^53^ of well-defined sigma-alkane complexes,^54^,^55^ typified by [Rh(R_2_PCH_2_CH_2_PR_2_)(η^2^η^2^-NBA)][BAr^F^_4_] [R = ^i^Bu, Cyp, Cy; NBA = norbornane; Ar^F^ = 3,5-(CF_3_)_2_C_6_H_3_]; Scheme 2a.^56^–^59^ The key to these complexes' relative stability in the solid-state is the arrangement of [BAr^F^_4_]^–^ anions that provide a well-defined cavity (*i.e.* they are “crystalline molecular flasks”,^60^,^61^ Scheme 2b) that results in very small changes in unit cell volumes and retention of crystallinity during the transformations of the organometallic cation. This allows for the characterization of products directly by single-crystal X-ray crystallography and solid-state NMR spectroscopy (SSNMR). These complexes, some of which are stable at room temperature (*e.g.* R = Cy, **[1-NBA][BAr^F^_4_]**), allow for the reaction chemistry of sigma-alkane complexes to be probed using solid/gas experimental techniques, for example C–H activation processes.^62^ Of relevance to this paper is the use of the alkane as a labile ligand that can be readily displaced in solid/gas reactivity and catalysis. We have recently reported that [Rh(^i^Bu_2_PCH_2_CH_2_P^i^Bu_2_)(η^2^η^2^-NBA)][BAr^F^_4_] reacts with ethene to form [Rh(^i^Bu_2_PCH_2_CH_2_P^i^Bu_2_)(ethene)_2_][BAr^F^_4_], that will catalyze ethene hydrogenation using solid/gas techniques, and also briefly commented on 1-butene isomerization.^63^

**Scheme 3:**
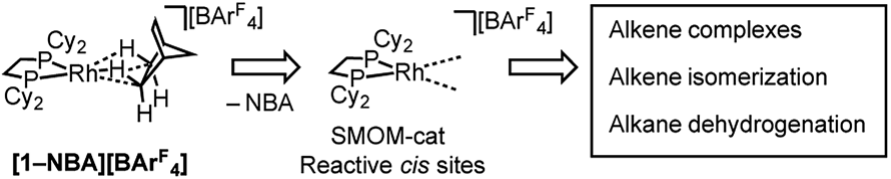
Generation of active *cis*-latent sites by solid/gas reactivity by displacement of a weakly bound alkane ligand.

This [Rh(^i^Bu_2_PCH_2_CH_2_P^i^Bu_2_)(η^2^η^2^-NBA)][BAr^F^_4_] system can suffer from loss of crystallinity in substitution reactions in the solid-state, as well as thermal instability to form the [BAr^F^_4_]^–^ coordinated zwitterion that is a poor catalyst. By contrast **[1-NBA][BAr^F^_4_]**, with its more rigid cyclohexyl groups, is stable as a crystalline solid for months at 298 K under an Ar-atmosphere, although on dissolution – even at very low temperature in CDFCl_2_ – the zwitterion [Rh(Cy_2_PCH_2_CH_2_PCy_2_)(η^6^-3,5-(CF_3_)_2_C_6_H_3_)BAr^F^_3_], **[1-BAr^F^_4_]**, is immediately formed reflecting the weak binding of the alkane ligand (*ca.* 80 kJ mol^–1^ or less).^54^–^56^ This weak binding, albeit stabilized in the solid-state, suggests that **[1-NBA][BAr^F^_4_]** may provide the ideal platform for studying solid/gas reactivity and catalysis in exceptionally well-defined molecular systems, providing a highly reactive {Rh(bis-phosphine)}^+^ fragment with *cis* vacant (or at least very weakly stabilized) sites, Scheme 3. We report here that this is the case, and show that the alkane ligand in **[1-NBA][BAr^F^_4_]** can be substituted for ethene, propene and 1-butene to give well-defined alkene complexes, some of which can only be prepared using such solid/gas routes. For propene and butene complexes rapid double-bond isomerization processes occur in the solid-state, which have been probed using variable temperature solid-state NMR spectroscopy, D-labelling studies and periodic DFT calculations. These exceptionally well-defined crystalline systems, which we term solid-state molecular organometallic catalysts (SMOM-Cat), are also active precatalysts for the solid/gas isomerization of 1-butene, demonstrating structure/activity relationships between the extended molecular structure and the measured catalytic activity. They also catalyze the transfer dehydrogenation/isomerization of butane to 2-butene.

## 2. Results and discussion

### 2.1 Synthesis of ethene complexes: **[1-(ethene)_2_][BAr^F^_4_]-Oct**

**Scheme 4:**
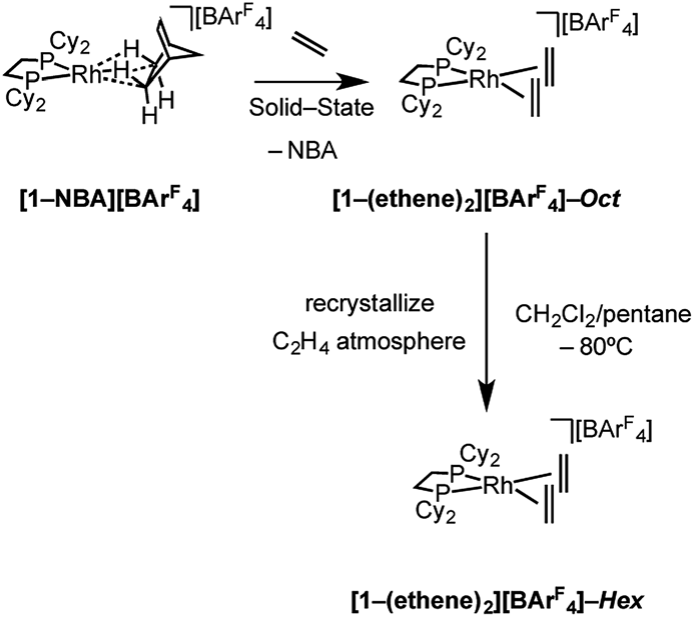
Synthesis of **[1-(ethene)_2_][BAr^F^_4_]** as octahedral (“*Oct*”, *C*2/*c*) and hexagonal (“*Hex*”, *P*6_3_22) polymorphs.

Addition of ethene (1 atm, 298 K) to a CD_2_Cl_2_ solution of [Rh(Cy_2_PCH_2_CH_2_PCy_2_)(η^6^-1,2-F_2_C_6_H_4_)][BAr^F^_4_], **[1-F_2_C_6_H_4_][BAr^F^_4_]**,^58^ resulted in the immediate formation of a new compound that displayed ^1^H and ^31^P NMR data that were consistent with the formation of an ethene complex. However there was also significant and rapid decomposition, with this new complex having a half-life of *ca.* 10 min in solution under these conditions. By contrast addition of ethene (1 atm, 2 h) to a single-crystalline sample of **[1-NBA][BAr^F^_4_]** resulted in the quantitative formation of the bis-ethene complex [Rh(Cy_2_PCH_2_CH_2_PCy_2_)(η^2^-C_2_H_4_)_2_][BAr^F^_4_], **[1-(ethene)_2_][BAr^F^_4_]-Oct**^64^ (Scheme 4) that is stable in the solid-state for at least 24 h, but over longer periods under an ethene atmosphere slow dehydrogenative coupling of ethene occurs to form a butadiene complex (see later). This transformation of **[1-NBA][BAr^F^_4_]** to **[1-(ethene)_2_][BAr^F^_4_]-Oct** results in the generation of one equivalent of free NBA, which manifests itself by a thin coating on the resulting single-crystals. SSNMR spectroscopy at 298 K provided diagnostic data indicative of reaction in the bulk. A single, but broad, environment was observed in the ^31^P{^1^H} SSNMR spectrum at *δ* 73.7, shifted 36 ppm upfield from **[1-NBA][BAr^F^_4_]**. In the ^13^C{^1^H} SSNMR spectrum a broad signal assigned to coordinated ethene was observed at *δ* 82.3.

Solution ^1^H and ^31^P{^1^H} NMR spectroscopy (CD_2_Cl_2_, Ar atmosphere, 193 K) of a freshly dissolved sample prepared in the solid-state were also fully consistent with formulation as a bis-ethene complex. In particular, at 193 K a sharp doublet at *δ* 73.6 [*J*(RhP) = 145 Hz] was observed, while in the ^1^H NMR spectrum bound ethene (8 H relative integral) was observed at *δ* 4.15. Warming to 298 K resulted in a broadening of all these signals, but no significant chemical shift change. After only 20 minutes at 298 K in CD_2_Cl_2_ solution significant decomposition had occurred, even when placed under an ethene atmosphere, to give unidentified products. Dissolving **[1-(ethene)_2_][BAr^F^_4_]-Oct** in 1,2-F_2_C_6_H_4_ solvent returned **[1-F_2_C_6_H_4_][BAr^F^_4_]**.

**Fig. 1:**
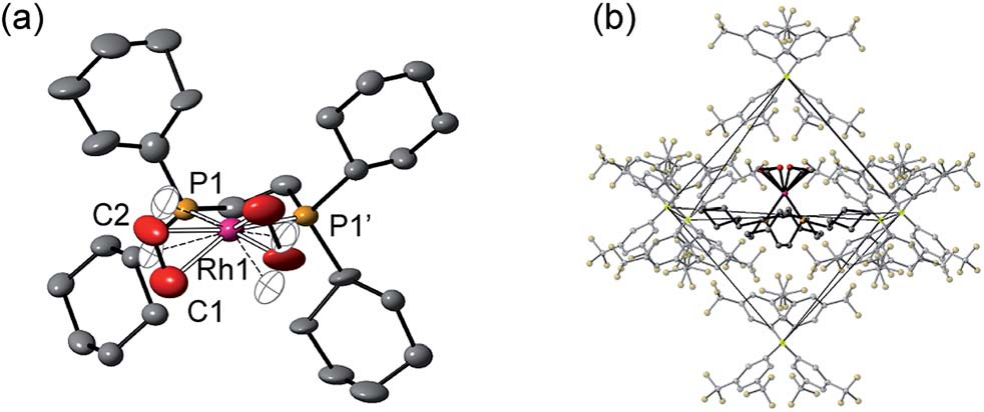
Solid-state structure of **[1-(ethene)_2_][BAr^F^_4_]-Oct**. (a) Cation showing the numbering scheme, displacement ellipsoids shown at the 50% probability level, 50% disorder component shown as open ellipsoids. (b) Local environment around the cation showing the arrangement of [BAr^F^_4_]^–^ anions. H-Atoms are omitted.

Remarkably, given that NBA is being expelled, this transformation is also a single-crystal to single-crystal one in the solid-state, as shown by an X-ray structure determination at 150 K. Starting from **[1-NBD][BAr^F^_4_]** (Scheme 2) this represents a rare example of a sequential reaction sequence for such processes.^50^ We suggest that the CF_3_ groups on the anions result in some plasticity of the solid-state lattice, which allows for the movement of the NBA,^65^ given that there are no significant channels in the crystal lattice. There is a space group change from to *P*2_1_/*n* (*Z* = 4) to *C*2/*c* (*Z* = 4) on substitution, and we,^56^,^59^ and others,^52^,^66^–^68^ have commented upon similar changes previously in solid/gas reactions. The final refined structural model (Fig. 1a) has a significant *R*-factor (10.7%) which we attribute to an increase in mosaicity on the single-crystal to single-crystal transformation and the loss of some high-angle data.

Nevertheless the refinement is unambiguous and shows a [Rh(Cy_2_PCH_2_CH_2_PCy_2_)(η^2^-C_2_H_4_)_2_]^+^ cation encapsulated by an almost perfect octahedron of [BAr^F^_4_]^–^ anions in the extended lattice (Fig. 1b). There is crystallographically imposed *C*_2_ symmetry. The ethene ligands on the Rh-center are disordered over two sites, with a C=C distance of 1.36(1) Å, consistent with a double bond and are also canted slightly from the square plane by 14°. Similar distortions have been noted in *trans*-[Rh{PR_2_(alkene)}_2_]^+^ species and are thought to be driven by enhanced π-back donation from the Rh d_*z*^2^_ orbital.^69^**[1-(ethene)_2_][BAr^F^_4_]-Oct** is stable to short periods of vacuum but satisfactory elemental analysis was not obtained as the NBA formed during the reaction was persistent and could not be removed.

**[1-(Ethene)_2_][BAr^F^_4_]-Oct** is a rare example of a bis- or tris-ethene adduct of a simple {Rh(PR_3_)_*n*_}^+^ fragment, which in solution are generally sensitive to loss of ethene.^70^–^72^ Bis-ethene complexes with other supporting ligand sets are more common. This scarcity no doubt reflects the instability of species such as **[1-(ethene)_2_][BAr^F^_4_]** in solution, and highlights the benefits of the solid/gas technique. This allows for **[1-(ethene)_2_][BAr^F^_4_]-Oct** to be reliably prepared in ∼0.2 g batches (unoptimized).

**Scheme 5:**
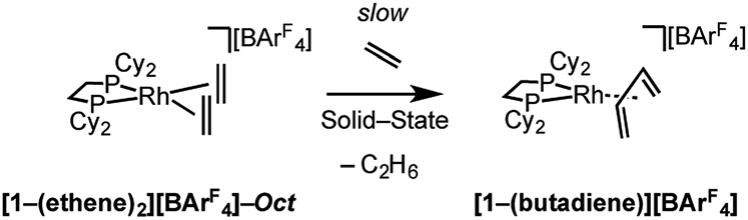
Dehydrocoupling of **[1-(ethene)_2_][BAr^F^_4_]**.

Over time, in the solid-state under an ethene atmosphere (1 atm), the butadiene complex, [Rh(Cy_2_PCH_2_CH_2_PCy_2_)(η^2^η^2^-C_4_H_6_)][BAr^F^_4_] **[1-(butadiene)][BAr^F^_4_]**, slowly forms (weeks), Scheme 5, as measured by ^31^P{^1^H} SSNMR. Interrogation of the head-space using gas-phase NMR spectroscopy after 1 week shows that approximately 1 equivalent of 2-butene is also formed, arising from initial ethene coupling and subsequent isomerization. **[1-(Butadiene)][BAr^F^_4_]** is better made directly from addition of excess 1-butene to **[1-F_2_C_6_H_4_][BAr^F^_4_]** in solution (see later). **[1-(Butadiene)][BAr^F^_4_]** presumably forms in the solid-state *via* dehydrocoupling and loss of H_2_, as previously reported for [Rh(^i^Bu_2_PCH_2_CH_2_P^i^Bu_2_)(ethene)_2_][BAr^F^_4_];^63^,^73^ possibly aided by sacrificial ethene, as ethane was also observed.

### 2.2 Synthesis of ethene complexes: **[1-(ethene)_2_][BAr^F^_4_]-Hex**

**Fig. 2:**
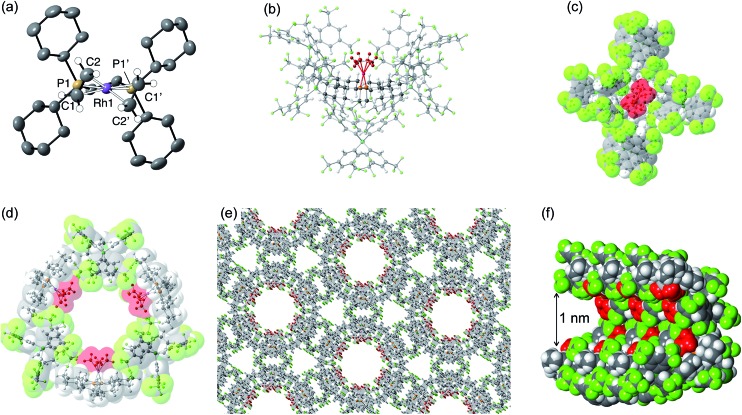
Solid-state structure of **[1-(ethene)_2_][BAr^F^_4_]-Hex** with the ethene groups colored in red [(b) to (f)] to highlight their positions. (a) Cation showing the numbering scheme, displacement ellipsoids shown at the 50% probability level; (b) Local environment around the cation showing the arrangement of [BAr^F^_4_]^–^ anions; (c) Van der Waals radii space-filling representation of (b) showing an alternate view highlighting the {Rh(ethene)_2_}^+^ fragment; (d) Van der Waals radii space-filling representation showing the packing arrangement leading to a solvent-accessible channel, as viewed down the *c*-axis; (e) Extended structure viewed down the *c*-axis; (f) Detail of a channel shown at the van der Waals radii highlighting the arrangement of {Rh(ethene)_2_}^+^ fragments.

Although **[1-(ethene)_2_][BAr^F^_4_]-Oct** is not stable in solution at 298 K, by working quickly under an atmosphere of ethene, with solvents already saturated with ethene, layering a CH_2_Cl_2_ solution of **[1-(ethene)_2_][BAr^F^_4_]-Oct** – as prepared by the solid/gas route – with pentane and recrystallization at –80 °C reliably affords small batches of crystals that are a polymorph of the starting material, **[1-(ethene)_2_][BAr^F^_4_]-Hex**. This space group change is from monoclinic *C*2/*c* (*Z* = 4) to hexagonal *P*6_3_22 (*Z* = 6). The quality of the refinement was reasonable (*R* = 6.6%). Fig. 2a shows the solid-state structure of an isolated cation, which demonstrates that this polymorph has a very similar cation compared with **[1-(ethene)_2_][BAr^F^_4_]-Oct**, [*e.g. d*(C=C) = 1.35(1) Å]. The major, unexpected, difference is that the [BAr^F^_4_]^–^ anions now do not form an octahedron, but are arranged so that only 5 surround the cation leaving a gap proximate to the {Rh(ethene)_2_}^+^ fragment (Fig. 2b). This results in ethene ligands that sit in a well-defined pocket of [BAr^F^_4_]^–^ anions (Fig. 2c). When inspected down the crystallographic *c*-axis the cations and anions are arranged under 3-fold symmetry so that they form a hexagonal structure of three ion pairs (Fig. 2d), resulting in cylindrical pores that run through the crystalline lattice (Fig. 2e). Moreover, these pores are decorated with the inward pointing {Rh(ethene)_2_}^+^ fragments, so that the ethene ligands are potentially accessible from the pore channels (Fig. 2f). Taking into account the van der Waals radii^74^ this pore-width is just less than 1 nm, and the calculated (PLATON^75^) solvent-accessible volume is 25%, making **[1-(ethene)_2_][BAr^F^_4_]-Hex** a microporous material.^76^ This compares with **[1-NBA][BAr^F^_4_]** and **[1-(ethene)_2_][BAr^F^_4_]-Oct** in which there are no solvent-accessible voids. These pores are presumably filled with solvent, but we find no definitive regions of electron density that we could assign to pentane (the most likely candidate) or CH_2_Cl_2_, and the calculated solvent-accessible volume likely represents the upper limit. There are other smaller trigonal prismatic pores, but these are formed from the CF_3_ groups of the [BAr^F^_4_]^–^ anion and do not contain any {Rh(ethene)_2_}^+^ fragments. Crystals of **[1-(ethene)_2_][BAr^F^_4_]-Hex** lose long range order when isolated in bulk by removal of solvent and rapid drying under vacuum, as measured by X-ray crystallography. We suggest this is due to loss of the disordered solvent in the pores, as ^1^H and ^31^P{^1^H} solution NMR spectroscopy of this material shows essentially identical signals to **[1-(ethene)_2_][BAr^F^_4_]-Oct** indicating that ethene has not been lost; while elemental analysis is consistent with the formulation. Material that retains its pore structure, as measured by a unit-cell determination of an isolated crystal, and that is useful for catalysis (*vide infra*) is prepared by rapid transfer of single-crystals from the cold mother liquor and drying using a flush of ethene.^77^ Using this methodology an acceptable yield of 62% (72 mg) is achieved. Material that has been exposed to an extended vacuum is not active for butene isomerization (see later).

Porous materials made from metal–organic frameworks (MOFs) are well known and can be used for a wide range of applications including gas separation and catalysis, and can often incorporate reactive metal sites as part of the framework,^52^,^78^–^83^ or as an encapsulated cation in an anionic porous network.^84^,^85^ However porous organometallic materials that are principally constructed from non-covalent interactions are less common.^51^,^86^–^89^ As far as we are aware **[1-(ethene)_2_][BAr^F^_4_]-Hex** represents a rare example where the likely site of any potential catalytic activity, that is labile ethene ligands, are focused directly into the pore, being similar to that reported by Brookhart for Ir(POCOP)(C_2_H_4_) [POCOP = κ^3^-C_6_H_3_-2,6-(OP(C_6_H_2_-2,4,6-(CF_3_)_3_)_2_)].^51^ The contrast between the extended structure of **[1-(ethene)_2_][BAr^F^_4_]-Oct** and its polymorph **[1-(ethene)_2_][BAr^F^_4_]-Hex** is dramatic. As demonstrated (Section 2.5) this leads to a significant difference in their ability to promote 1-butene isomerization catalysis when in single-crystalline form.

### 2.3 Synthesis of a propene complex: **[1-(propene)][BAr^F^_4_]**

**Scheme 6:**
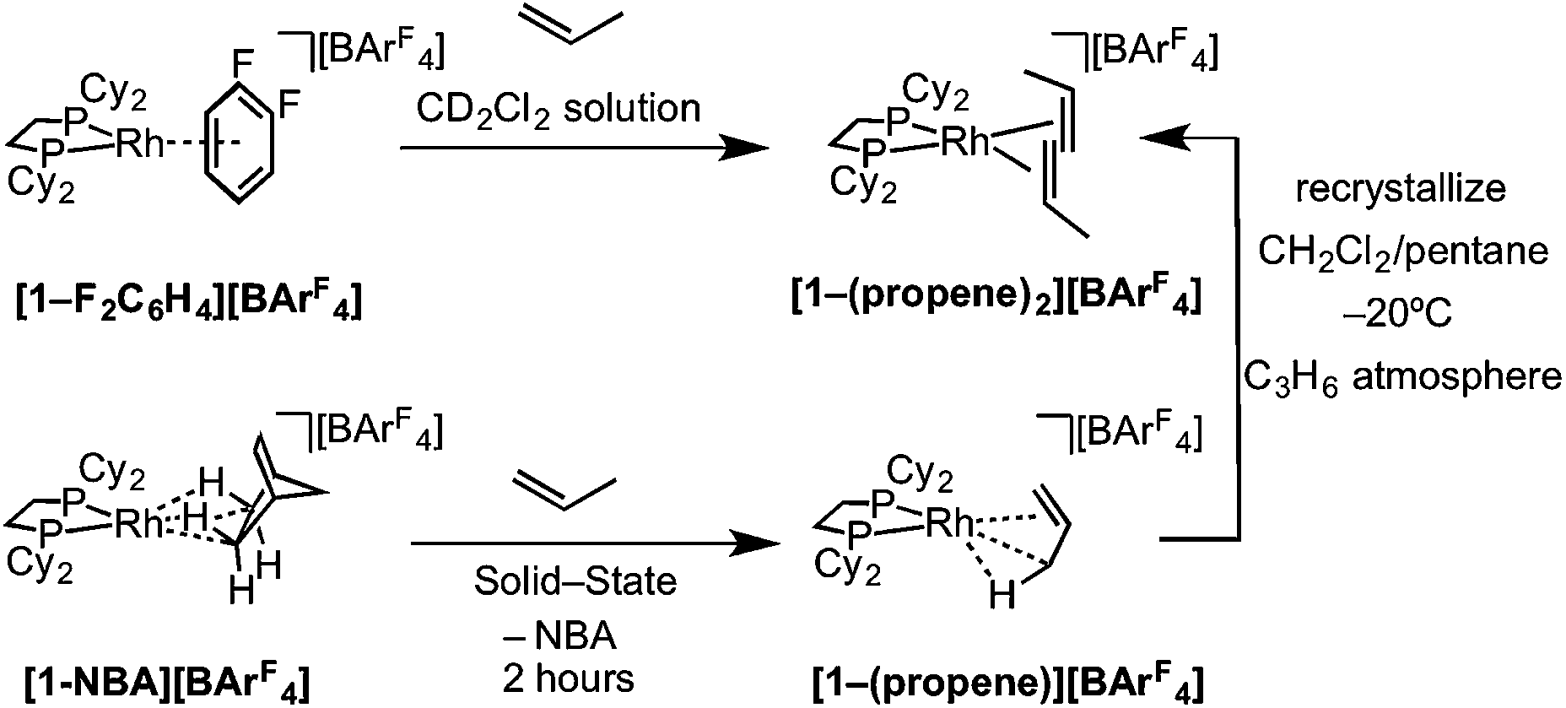
Synthesis of **[1-(propene)*_x_*][BAr^F^_4_]** by solution (*x* = 2) and solid/gas (*x* = 1) single-crystal to single-crystal reactivity.

The solid/gas reaction of propene with **[1-NBA][BAr^F^_4_]** over 2 hours, with its weakly-bound NBA alkane ligand, led to the formation in the solid-state of a new complex shown to be [Rh(Cy_2_PCH_2_CH_2_PCy_2_)(η^2^-C_3_H_6_)][BAr^F^_4_], **[1-(propene)][BAr^F^_4_]**, which we show to have one propene ligand η^2^-bound with a supporting γ-agostic Rh···H_3_C interaction, and the ∼*O*_h_ arrangement of anions retained. This is also a single-crystal to single-crystal transformation that allows for the molecular structure to be determined directly by single-crystal X-ray diffraction. Shorter reaction times (1 hour) led to incomplete reaction as evidenced by the formation of **[1-BAr^F^_4_]** (that comes from displacement of NBA in **[1-NBA][BAr^F^_4_]**^56^) on dissolution in cold CD_2_Cl_2_ (198 K). By contrast, addition of propene gas to a CD_2_Cl_2_ solution of **[1-F_2_C_6_H_4_][BAr^F^_4_]**^58^ resulted in a product assigned to **[1-(propene)_2_][BAr^F^_4_]** being formed [*ca.* 70% by ^31^P{^1^H} NMR spectroscopy, *δ*(^31^P) 91.0 *J*(RhP) = 176 Hz], the rest being unreacted **[1-F_2_C_6_H_4_][BAr^F^_4_]** (Scheme 6). These data differ slightly – but significantly – from those which come from material prepared by the solid/gas route (*vide infra*). Crystalline material of this bis-propene adduct can be obtained by recrystallization of **[1-(propene)][BAr^F^_4_]**, as prepared by solid/gas routes, from CH_2_Cl_2_/pentane saturated with propene at low temperature (–20 °C). A single-crystal X-ray determination confirms the formulation (see ESI†) showing a similar structure to the cation **[1-(ethene)_2_][BAr^F^_4_]**, with the two propene ligands canted from the square plane by 29.4°,^90^ but now with a distorted-octahedral arrangement of [BAr^F^_4_]^–^ anions, similar to that observed for **[1-(pentane)][BAr^F^_4_]**.^58^ Solid-state and solution routes, thus, lead to different products: mono- and bis-propene respectively.

**Fig. 3:**
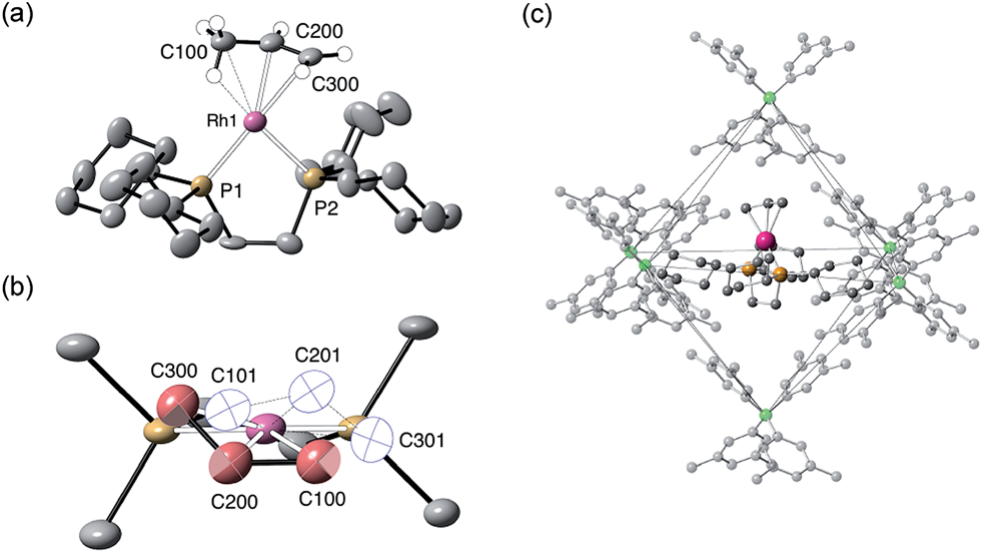
Solid-state structure of **[1-(propene)][BAr^F^_4_]**. Displacement ellipsoids are shown at the 30% probability level. (a) Cation (only one disordered component) with selected hydrogen atoms shown (placed in calculated positions); (b) Disordered propene ligand (with the two components shown in red and white); (c) Packing of the [BAr^F^_4_]^–^ anions with fluorine atoms omitted for clarity.

**Scheme 7:**
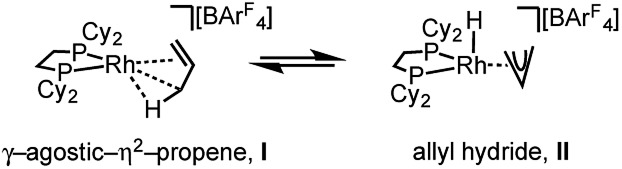
Agostic/η^2^ and allyl-hydride tautomers of **[1-(propene)][BAr^F^_4_]**.

The solid-state structure of **[1-(propene)][BAr^F^_4_]** is shown in Fig. 3. The C_3_-hydrocarbon is disordered over two positions, related by a non-crystallographic two-fold rotation, Fig. 3b; while the octahedral arrangement of anions is retained, Fig. 3c. As for **[1-(ethene)_2_][BAr^F^_4_]-Oct** the solid/gas reaction led to loss in high-angle data and a reduction in the quality of the refinement (*R* = 12.7%). This, alongside the disordered organic fragment, means that a detailed discussion of the bond lengths and angles is not appropriate, and the hydrogen atoms were placed in calculated positions. Although the two C–C distances appear to show differentiation between C–C and C=C bonds [*e.g.* C100–C200, 1.361(9); C200–C300, 1.239(9) Å], both measure shorter than might be expected (and calculated, *vide infra*)^91^,^92^ which likely is a consequence of the poor structure and rotational disorder. All three Rh–C distances reflect Rh–C bonding interactions, but within error are the same [*e.g.* 2.15(2)–2.29(3) Å]. Thus, although the gross structure is unambiguous in showing a single C_3_ fragment bound to the metal center, whether it is an η^2^-bound propene with a supporting γ-agostic^93^ interaction (**I**, Scheme 7) or the isomeric allyl-hydride, that arises from γ-C–H activation of propene (**II**),^22^,^49^,^94^–^97^ cannot be determined due to the quality of the data. We thus turned to variable temperature SSNMR and solution NMR spectroscopy, as well as periodic DFT calculations, to determine the precise structure. These studies show that at low temperatures the alkene/agostic tautomer is favored, which at higher temperatures accesses the allyl-hydride in both solid-state and solution.

The 158 K ^31^P{^1^H} SSNMR spectrum of **[1-(propene)][BAr^F^_4_]**, prepared *in situ*, shows two broad environments at *δ* 101.3 and 90.4, consistent with the two different phosphorus environments in the single-crystal X-ray structure. At this low temperature in the ^13^C{^1^H} SSNMR spectrum two, approximately equal intensity, signals are observed in the region associated with bound alkene ligands,^98^ at *δ* 94.2 and 78.8, alongside a high-field signal at *δ* 6.5 in the region indicative of an agostic M···H_3_C interaction.^99^ Warming to 298 K results in a broad, but asymmetric, ^31^P signal in the SSNMR spectrum at *δ* 95.6; while in the ^13^C{^1^H} SSNMR spectrum a broad signal was observed at *δ* 93.7, and the high-field signal present at 158 K was absent. These data suggest a fluxional process is occurring in the solid-state at room temperature,^100^ that is slowed at 158 K. Low temperature ^1^H/^13^C HETCOR experiments, that we^56^,^62^ and others^101^ have previously shown to be useful in determining ^1^H NMR chemical shifts for sigma interactions in the solid-state, were not successful. The variable temperature ^31^P{^1^H} NMR data have been modelled using rate-constants derived from a line-shaped analysis, and a resulting Eyring analysis gives Δ*G*^‡^ = 10(1) kcal mol^–1^ and Δ*S*^‡^ = –7(3) cal K^–1^ suggesting a slightly ordered transition-state.

Similar behavior is observed using solution NMR spectroscopy when **[1-(propene)][BAr^F^_4_]**, prepared by the solid/gas route, is dissolved in CD_2_Cl_2_. Although rapid decomposition (less than 30 minutes) occurs at 298 K to give unidentified products, immediate data collection led to reliable solution NMR data. At 298 K the ^31^P{^1^H} NMR spectrum shows a single environment *δ* 95.2 [*J*(RhP) = 181 Hz], while the ^1^H NMR spectrum shows a very broad signal at *δ* 5.07 of relative integral ∼1 H in addition to signals in the aliphatic and aryl regions. Such a chemical shift is characteristic of the methine proton in an η^3^-allyl ligand.^98^,^102^ The hydride region was featureless. These data suggest a fluxional process is also occurring in solution at 298 K. Cooling to 193 K in CD_2_Cl_2_ slows both decomposition and the fluxional process. The ^31^P{^1^H} NMR spectrum now shows two environments, *δ* 100.4 [*J*(RhP) = 200 Hz] and *δ* 89.9 [*J*(RhP) = 161 Hz], similar to those measured in the SSNMR spectrum at 158 K, with the larger coupling constant suggesting a weakly-bound *trans* ligand. The ^1^H NMR solution spectrum at 193 K shows three integral 1-H environments in the alkene region [*δ* 4.84, 4.54, 3.55] and high-field integral 3-H signal at *δ* –0.02 assigned to the methyl group that includes the agostic C–H···Rh interaction that is undergoing rapid rotation. The signals at *δ* 4.54, 3.55 and –0.02 become broad on warming, and disappear into the baseline at 253 K suggesting that they are mutually exchanging. In contrast the signal assigned to the methine proton remains essentially unchanged in chemical shift, and can be tracked to the broad signal observed at *δ* 5.07 at 298 K.

**Fig. 4:**
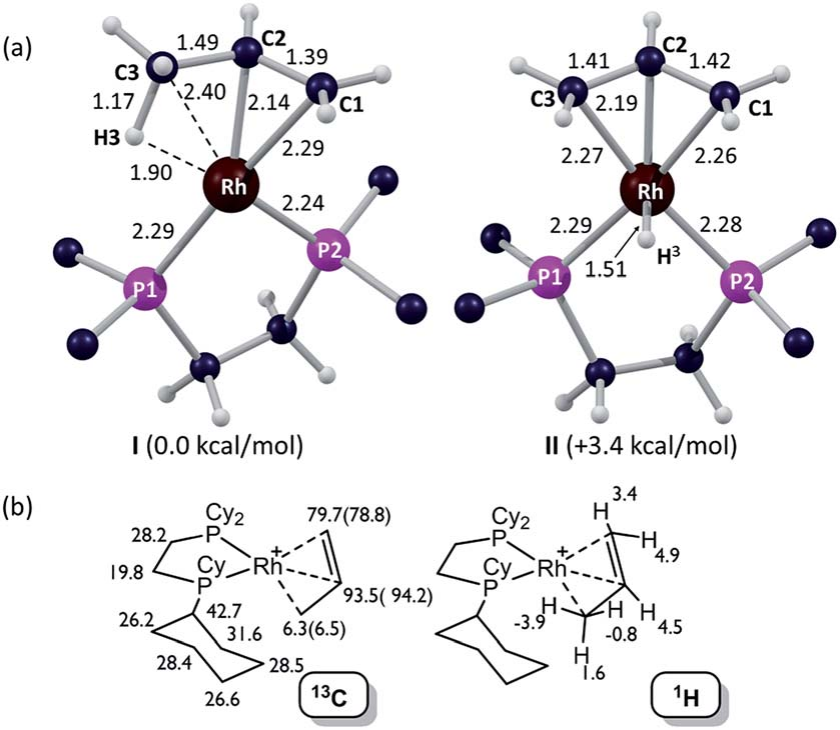
Computational characterization of **[1-(propene)][BAr^F^_4_]** in the solid-state: (a) structures of the molecular cation in its [1-(propene)]^+^ and [1-(allyl-hydride)]^+^ forms (**I** and **II** respectively, selected distances in Å; truncated Cy groups); (b) computed ^13^C and ^1^H data for the [1-(propene)]^+^ cation, with selected ^13^C experimental data (SSNMR, 158 K) in parenthesis.

Insight into the detailed structure of the propene adduct **[1-(propene)][BAr^F^_4_]** was obtained *via* periodic density functional theory (DFT) calculations at the PBE-D3 level, where this approach has previously been shown to reproduce the solid-state structures and fluxionality of related sigma-alkane complexes very effectively.^54^,^58^ Geometry optimization of **[1-(propene)][BAr^F^_4_]** based on one component of the crystal structure (using propene carbon positions C100, C200 and C300, see Fig. 3) confirmed the presence of an η^2^-propene ligand that also engages in a γ-agostic interaction with the metal center (Rh···C3 = 2.40 Å; Rh···H3 = 1.90 Å; C3–H3 = 1.17 Å; see Fig. 4a for the labelling scheme used in the computational studies). The agostic interaction lies in the {P^1^RhP^2^} plane (the {P1RhP2}/{RhC3H3} interplane angle = 7.3°) whereas the C1=C2 double bond is rotated by 52.3°. The extended solid-state structure is also well reproduced (see ESI† for an overlay of experimental and computed structures). The energy of this η^2^-propene cation within the extended lattice, **I**, was computed to lie 3.4 kcal mol^–1^ below an alternative η^3^-allyl hydride cation, **II**; however, the latter is computed to be kinetically accessible in the solid-state (see below). Addition of a second propene molecule to **I** to form a bis-η^2^-propene adduct in the solid-state was computed to be endergonic by 4.7 kcal mol^–1^. In contrast, in solution, molecular calculations indicate the formation of [Rh(Cy_2_PCH_2_CH_2_PCy_2_)(η^2^-C_3_H_6_)_2_]^+^ from free propene and [Rh(Cy_2_PCH_2_CH_2_PCy_2_)(η^2^-C_3_H_6_)]^+^ is exergonic by 9.5 kcal mol^–1^, and this bis-propene adduct is accessible experimentally in solution (see above).

Fig. 4b provides calculated ^13^C and ^1^H chemical shifts associated with the **[1-(propene)]^+^** cation in the solid-state, based on GIPAW calculations on the extended **[1-(propene)][BAr^F^_4_]** structure. Excellent agreement is found with the experimental low temperature ^13^C SSNMR data for the propene ligand, providing further support for the formulation of an η^2^-propene/γ-agostic complex. The calculations assign a high-field ^1^H resonance of *δ* –3.9 to the agostic proton in the ^1^H NMR spectrum of the static structure, while the average chemical shift computed for all three methyl protons is *δ* –1.0 that reflects a dynamic CH_3_ group. This is to the high field of the observed value of *δ* –0.02 in solution and may reflect environment effects in the solid-state. Thus when the model used in the calculation is changed to the isolated cation an average value of *δ* –0.1 is computed, with the geminal protons in particular shifting to lower field (*δ*_calc_(^1^H) +2.3, +0.9). In contrast, the agostic proton is less sensitive to the model employed, shifting by only 0.4 ppm to *δ*_calc_ –3.5 in the isolated cation. Local ring current effects arising from proximal aryl groups of the [BAr^F^_4_]^–^ anion have previously been shown to be significant for **[1-NBA][BAr^F^_4_]** in the solid-state.^55^

One possible mechanism for the fluxional process observed experimentally at room temperature in solution and the solid-state is a 1,3-hydrogen shift involving C–H activation of the bound propene in **I** to give the allyl-hydride **II** (Scheme 7), followed by reinsertion, either degenerate or onto the distal carbon atom. A similar process has been suggested for the double bond shift in Ir–pincer systems such as Ir(POCOP^*t*Bu^)(η^2^-propene) [POCOP^*t*Bu^ = κ^3^-C_6_H_3_-2,6-(OP^*t*^Bu_2_)_2_].^22^ The 1,3-hydrogen shift would result in a formal double bond isomerization in propene, but proceeds with no overall chemical change to the complex. If this was happening rapidly^94^ at room temperature, and such an equilibrium favored the propene tautomer, then a hydride signal would likely not be observed in the ^1^H NMR spectrum. In contrast, as the proton associated with the central carbon (C2 in Fig. 4) does not undergo rapid exchange, it should be observed, and we propose that this corresponds to the signal at *δ* 5.07 in the 298 K solution ^1^H NMR spectrum.

**Scheme 8:**
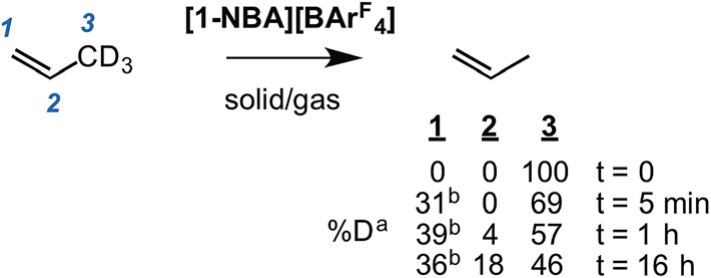
Catalytic D-label scrambling in 3,3,3-d_3_-propene_(g)_ by solid/gas reactivity using **[1-NBA][BAr^F^_4_]** as a catalyst. ^a^Measured by gas-phase ^2^H NMR spectroscopy, error estimated ±5%. Statistical distribution between all three positions (%): 33 : 17 : 50. ^b^*cis* : *trans* D observed in an ∼1 : 1 ratio as measured by ^1^H NMR spectroscopy in the gas phase.

To probe this fluxional process further, solid/gas catalysis using **[1-NBA][BAr^F^_4_]** and 3,3,3-d_3_-propene was performed using ∼6 equivalents of alkene at 298 K and the headspace gas interrogated using ^2^H NMR spectroscopy (Scheme 8). After 5 minutes deuterium was now observed in both the C1 alkene (*cis* and *trans* positions relative to the methyl) and the methyl positions, and after 1 hour D-incorporation approached that expected for a statistical distribution at the C1 and C3 positions [0.39 : 0.57 ratio]. A very small amount of D-incorporation into the C2 methine position (4%) was also measured at this time. After 16 hours all positions were deuterated to a level close to that predicted from a simple statistical distribution between all three positions. The rapid D-scrambling at the C1 and C3 positions is fully consistent with a mechanism for fluxionality that invokes facile C–H activation *via* an allyl-intermediate.^24^ This rapid catalytic solid/gas H/D scrambling in 3,3,3-d_3_-propene using **[1-NBA][BAr^F^_4_]** can be compared to that measured in solution phase under stoichiometric conditions for Ir(POCOP^*t*Bu^)(η^2^-d_3_-propene) that requires heating (∼40 h at 333 K),^22^ or the slow (greater than 16 h) solid/gas reactivity of [(Ph_3_P)_3_IrH_2_]_3_[PW_12_O_40_] with 3,3,3-d_3_-propene that results in intramolecular scrambling in the final allyl-hydride product.^49^ Interestingly, that this solid/gas catalysis is much faster (∼5 minutes) compared with bulk-scale synthesis of **[1-(propene)][BAr^F^_4_]** (2 h) suggests that the most active sites are at, or near, the surface. We have previously drawn similar conclusions regarding the use of [Rh(^i^Bu_2_PCH_2_CH_2_P^i^Bu_2_)(η^2^η^2^-C_4_H_6_)][BAr^F^_4_] as a solid-state ethene hydrogenation catalyst.^63^ As we discuss later, these observations are consistent with the relative rates of 1-butene isomerization by **[1-NBA][BAr^F^_4_]** for different-sized crystalline samples.

**Scheme 9:**
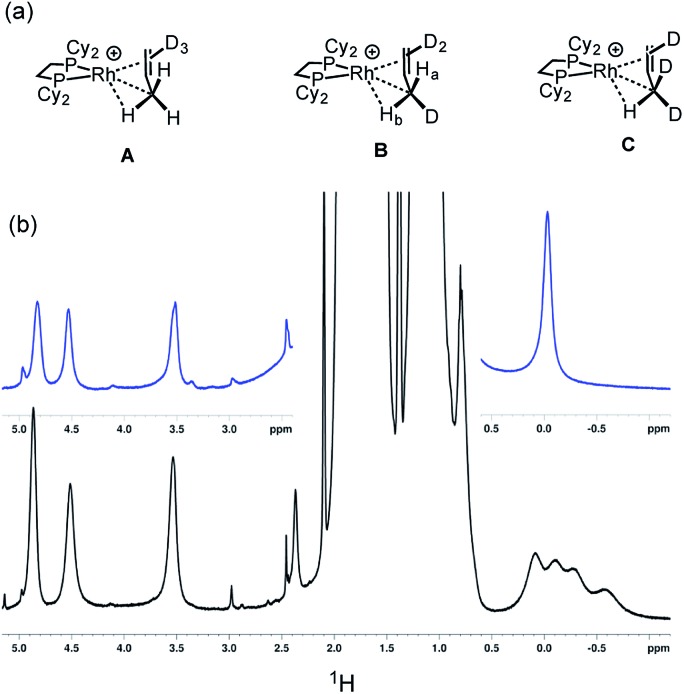
(a) Isotopomers **[1-(d_3_-propene)][BAr^F^_4_]** highlighting the methyl substitution patterns. (b) ^1^H NMR spectra (193 K, CD_2_Cl_2_) of a sample prepared by the solid/gas route after 16 hours. Top, **[1-(propene)][BAr^F^_4_]**; bottom, **[1-(d_3_-propene)][BAr^F^_4_]**.

Further evidence for both the agostic interaction and an exchange process occurring in **[1-(propene)][BAr^F^_4_]** comes from interrogation of a number of samples prepared using 3,3,3-d_3_-propene after 16 hours, in which the D-label would be expected to be in all three C-positions (*i.e.*Scheme 8).^103^ The corresponding γ-agostic signal in the 193 K solution ^1^H NMR spectrum integrates to a total of 1.5 protons, as expected for the statistical distribution of deuterium, and comes from an ensemble combination of CH_3_ and CDH_2_ and CD_2_H groups (**A**, **B** and **C**, Scheme 9). A significant isotopic perturbation of equilibrium (IPE) would be thus expected to be observed in the ^1^H NMR spectrum for the three CH_3_, CH_2_D and CHD_2_ isotopomers, as, due to zero-point energy differences between C–H and C–D, agostic Rh···H–C interactions are favored.^104^

However four signals are observed at *δ* 0.08, –0.11, –0.28 and –0.58 in the 500 MHz ^1^H NMR spectrum at 193 K.^105^ We suggest that the extra signal comes from the diastereomeric pair in the CH_2_D isotopomer that arises from the relative orientation of the C–D, agostic and alkene bonds so that the two hydrogen atoms cannot become equivalent by a simple rotation (H_a_ and H_b_ in structure **B**). These data fully support the presence of an agostic interaction in **[1-(propene)][BAr^F^_4_]**. Chemical shift calculations on the isolated cation of these isotopomers, taking into account the respective Boltzmann weighting factors recreate the observed relative chemical shifts well (*δ* –0.1, –0.24, –0.41 and –0.62, ESI†). The lowest-field signal for the agostic C–H, experimentally observed at *δ* 0.08, is assigned to isotopomer **A** and would be expected to have a very similar chemical shift to that observed in per-protio **[1-(propene)][BAr^F^_4_]**, *δ* –0.02, assuming any intrinsic chemical shift change is small.^106^ We speculate that this difference in chemical shift may be due to a small, but significant, equilibrium concentration of the (close in energy) allyl-hydride being present on isotopic substitution at low temperature, that is not observed in the ^1^H NMR spectrum at low temperature due to a combination of low abundance and broad signals. Three signals are observed for the alkene protons, that are slightly shifted from the per-protio complex: *δ* 4.87, 4.52 and 3.54 each integrating to 0.5 H. The isotopomers are not resolved in these signals.

**Scheme 10:**
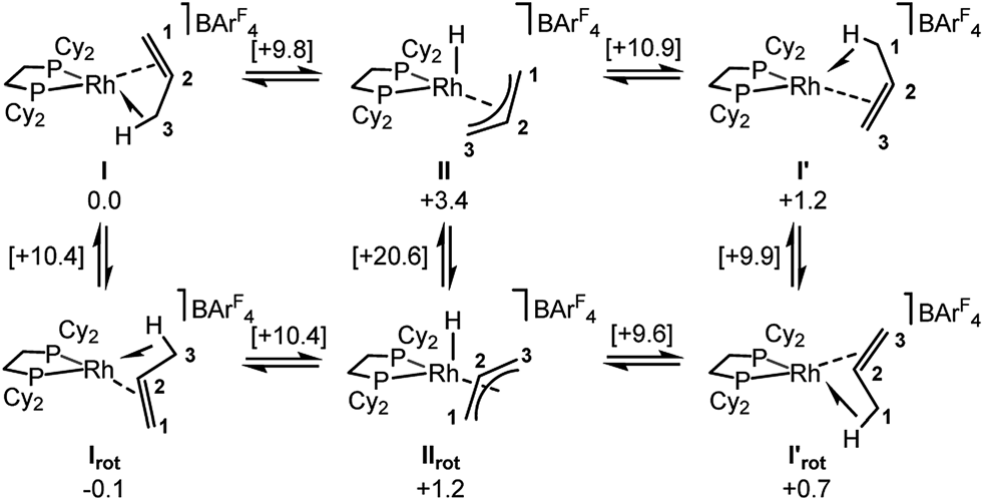
Computed mechanisms for fluxionality and D-label scrambling in [**1-(propene)][BAr^F^_4_]** in the solid-state. Gibbs free energies are indicated in kcal mol^–1^ with those for transition states provided in square brackets.

Periodic DFT calculations have also been used to explore the fluxionality and related H/D exchange processes associated with the propene ligand in the solid-state (Scheme 10). Starting from cation **I** (0.0 kcal mol^–1^), oxidative cleavage of the agostic C3–H bond proceeds with a barrier of 9.8 kcal mol^–1^ to give allyl-hydride **II** at +3.4 kcal mol^–1^. C1–H reductive coupling then proceeds *via* a transition state at +10.9 kcal mol^–1^ to reform the propene complex as **I′** (+1.2 kcal mol^–1^) in which the alkene and agostic moieties have swapped positions compared to **I**. This exchange process renders the two phosphorus centers near-equivalent with a modest overall barrier of 10.9 kcal mol^–1^, consistent with it being readily accessible at room temperature.^107^ The slightly different energies of **I** and **I′** (and the transition states linking these structures to **II**) reflect the different orientations of the propene ligand within the crystal lattice.^108^ In addition to this net 1,3-H shift, rotation of the propene ligand is also readily accessible, with a barrier of 10.4 kcal mol^–1^ interconverting **I** and its rotated form **I_rot_**, while **I′** and **I′_rot_** are linked *via* a transition state at 9.9 kcal mol^–1^. These rotated forms correspond to the alternative orientation of the propene ligand seen crystallographically (defined by positions C101, C201 and C301, Fig. 3) and their similar energies (in particular **I** and **I_rot_** are within 0.1 kcal mol^–1^) are consistent with the approximately 50 : 50 occupation of these two components in the solid-state structure. These calculated barriers to fluxionality compare very well with that derived experimentally (Δ*G*^‡^(exp) = 10(1) kcal mol^–1^). An alternative rearrangement *via* rotation of the η^3^-allyl ligand (**II** to **II_rot_**) involves a transition state at +20.6 kcal mol^–1^ and so is not competitive.

The γ-agostic interaction observed in the ground state structure of **[1-(propene)][BAr^F^_4_]** is directly related to C–H activation transition states calculated for the isomerization of η^2^-bound alkenes *via* allyl-hydride intermediates,^22^ and closely related to those calculated for β-methyl migration from alkyl groups – the microscope reverse of the chain propagation step in olefin polymerization.^109^

### 2.4 **[1-(Butene)][BAr^F^_4_]** and **[1-(butadiene)][BAr^F^_4_]**

**Scheme 11:**
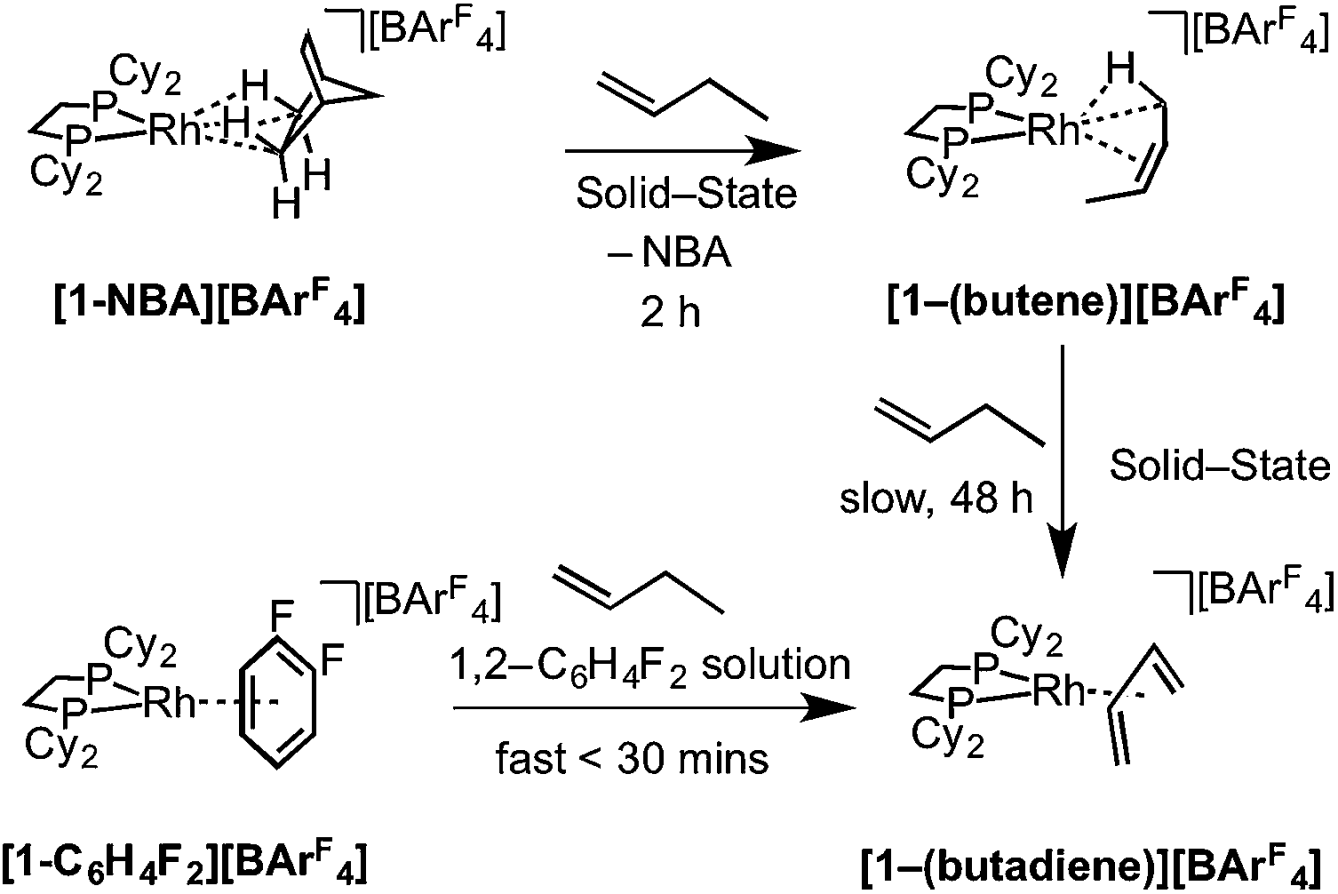
Synthesis of **[1-(butene)][BAr^F^_4_]** and **[1-(butadiene)][BAr^F^_4_]** by solid/gas and solution routes.

**Scheme 12:**
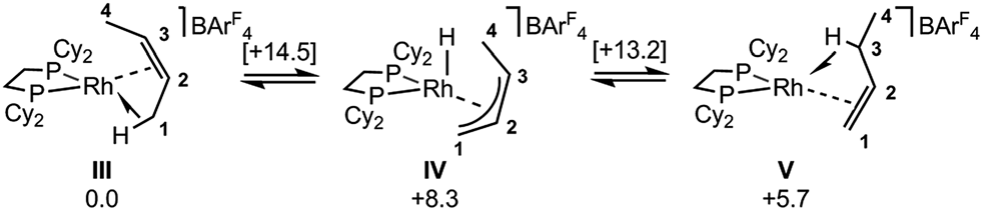
Computed mechanisms for interconversion of **[1-(*cis*-2-butene)]^+^**, **III**, and **[1-(1-butene)]^+^**, **V**, cations in the solid-state. Free energies are indicated in kcal mol^–1^ with those for transition states provided in square brackets.

As for propene, excess 1-butene can be added to **[1-NBA][BAr^F^_4_]** resulting in a solid/gas single-crystal to single-crystal reaction over 2 h to give **[1-(butene)][BAr^F^_4_]**. Shorter reaction times (∼20 minutes) led to incomplete reaction as evidenced by the formation of **[1-BAr^F^_4_]**^56^ on dissolution in cold CD_2_Cl_2_. By contrast, addition of excess 1-butene to **[1-F_2_C_6_H_4_][BAr^F^_4_]** in CD_2_Cl_2_ solution resulted in the rapid formation of **[1-(butadiene)][BAr^F^_4_]** (Scheme 11). Over time (48 h) in the solid-state under a butene atmosphere (1 atm) **[1-(butene)][BAr^F^_4_]** converts to **[1-(butadiene)][BAr^F^_4_]**, as measured by ^31^P{^1^H} SSNMR spectroscopy. Thus samples of **[1-(butene)][BAr^F^_4_]** are always contaminated with small amounts of recalcitrant **[1-(butadiene)][BAr^F^_4_]**. We suggest that **[1-(butadiene)][BAr^F^_4_]** forms *via* an intermolecular process involving transfer dehydrogenation to exogenous butene. Consistent with this butane was observed to be formed in the headspace. A likely mechanism is one of interception of an associated allyl-hydride (*e.g.***IV**Scheme 12) by butene and subsequent β-elimination/reductive elimination of butane.^63^ We suggest that in the solid-state such an intermolecular process is attenuated as it requires the coordination of two equivalents of the C4-alkene which is disfavored (as computed for the bis-propene analogue) by the local crystalline environment around the metal cation due to intermolecular steric effects within the anion-cage. Despite **[1-(butadiene)][BAr^F^_4_]** being formed by a solid/gas reaction with crystalline **[1-NBA][BAr^F^_4_]** we have not been able to determine its solid-state structure by single-crystal X-ray diffraction.

**Fig. 5:**
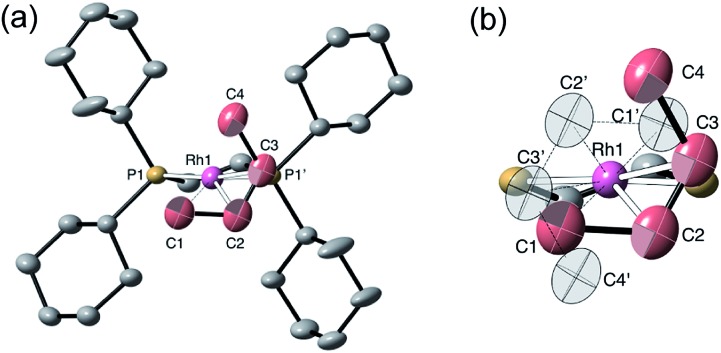
Solid-state structure of **[1-(butene)][BAr^F^_4_]** at 150 K. Ellipsoids are shown at the 50% displacement level. (a) Showing the cation and one of the disordered *cis*-butene ligands, hydrogen atoms omitted for clarity; (b) the two, symmetry-related, disordered components. Selected bond distances (Å) Rh–C1, 2.37(2); Rh–C2, 2.25(2); Rh–C3, 2.23(2); Rh–C4, 2.83(2) Å; C1–C2, 1.52(3); C2–C3, 1.33(3); C3–C4, 1.62(3) Å.

**Fig. 6:**
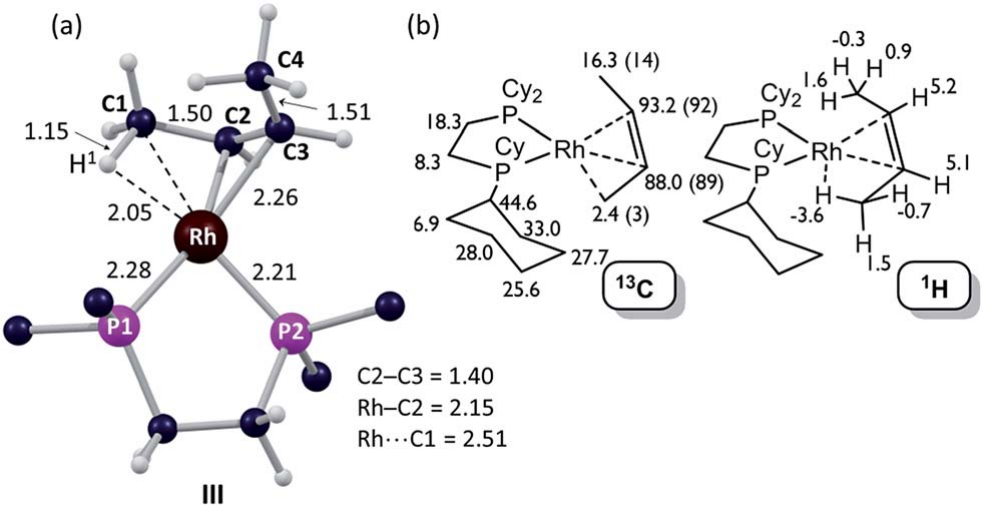
Computational characterization of **[1-(butene)][BAr^F^_4_]** in the solid-state: (a) structure of the molecular cation, **III**, with selected distances in Å (truncated Cy groups; input geometry based on the C1–C4 positions in the X-ray analysis, see Fig. 5); (b) computed ^13^C and ^1^H data for **[1-(butene)][BAr^F^_4_]** (cation only) with selected ^13^C experimental data (SSNMR, 158 K) in parenthesis.

The single-crystal X-ray structure of **[1-(butene)][BAr^F^_4_]** prepared by the solid/gas route shows a {Rh(Cy_2_PCH_2_CH_2_PCy_2_)}^+^ moiety on which a *cis*-2-butene ligand can be successfully modelled (150 K, space group *C*2/*c*, *Z* = 4, Fig. 5). Unfortunately, the alkene ligand bound with the metal is disordered over two sites (crystallographically imposed) which when coupled with the loss in high-angle data on the solid/gas transformation means that bond metrics have an associated significant error, and the hydrogen atoms associated with the butene fragment were not located. Nevertheless the structure is clear, and very closely related to that of **[1-(propene)][BAr^F^_4_]**. More structural detail is provided by periodic DFT calculations on **[1-(butene)][BAr^F^_4_]** that provide firm evidence for an η^2^-binding mode supported by a γ-agostic interaction from one methyl (see Fig. 6a). Although butene is introduced as the 1-isomer it is 2-butene that is predominately bound to the metal center, in its *cis*-form. This is verified by vacuum transfer of CD_3_CN onto **[1-(butene)][BAr^F^_4_]** to form the CD_3_CN adduct, [Rh(Cy_2_PCH_2_CH_2_PCy_2_)(CD_3_CN)_2_][BAr^F^_4_] **[1-(CD_3_CN)_2_][BAr^F^_4_]** that is itself a poor isomerization catalyst, and free butene, followed by a further vacuum transfer of the condensable volatiles. Analysis by ^1^H NMR spectroscopy showed *cis*-2-butene to be dominant: *δ* 1.59 [CDCl_3_, d, *J*(HH) = 4.9 Hz, CH_3_].^110^ As we show next, this is fully consistent with the low temperature solution and SSNMR spectra, and DFT calculations that show 2-butene to be bound as the *cis*-isomer.

The ^31^P{^1^H} SSNMR spectrum of **[1-(butene)][BAr^F^_4_]** at 298 K shows two closely separated environments at *δ* 98.4 and 95.1, as well as a small amount of **[1-(butadiene)][BAr^F^_4_]***δ* 81.0. In the ^13^C{^1^H} SSNMR spectrum a single broad environment is observed at *δ* 91.8 in the region associated with the alkene ligand. A broad signal at *δ* 6.3 is also observed, which may point to an agostic interaction. Cooling to 158 K resolved the ^31^P{^1^H} SSNMR spectrum into two clear environments [*δ* 100.0, 93.3]; while in the ^13^C{^1^H} SSNMR spectrum two signals are now observed in the alkene region [*δ* 92.1, 89.3] alongside a high field signal at *δ* 3.4. Another relatively high field signal at *δ* 14.3 is also present. These data are consistent with the 150 K single-crystal X-ray structure (Fig. 5) and the structure computed in the solid-state by DFT [Fig. 6a], and point to a fluxional process in the solid-state at 298 K, that is slowed at lower temperatures, while retaining one agostic Rh···H_3_C interaction (*i.e. δ* 3.4) and one non-agostic methyl (*i.e. δ* 14.3). Computed NMR data for **[1-(butene)][BAr^F^_4_]** featuring a *cis*-2-butene ligand also correspond well to this being the ground state structure [Fig. 6b]. We were not successful in obtaining a meaningful ^1^H/^13^C HETCOR spectrum, as for **[1-(propene)][BAr^F^_4_]**.

Dissolving **[1-(butene)][BAr^F^_4_]** (prepared by solid/gas route) in CD_2_Cl_2_ at 193 K, allows more details to be revealed of the structure of this complex, which also point towards *cis*-2-butene being bound. The ^31^P{^1^H} NMR spectrum at this temperature shows two clearly resolved doublets of doublets: *δ* 97.5 [*J*(RhP) = 211, *J*(PP) 24 Hz] and *δ* 89.9 [*J*(RhP) = 159, *J*(PP) 24 Hz]. These, as for **[1-(propene)][BAr^F^_4_]**, indicate a weakly bound ligand *trans* to one phosphorus environment – likely the agostic interaction observed in the solid-state structure. The ^13^C{^1^H} NMR solution spectrum shows a single environment in the alkene region, *δ* 90.2, and a single high-field signal, *δ* 10.9, assigned to the methyl groups. Both these signals are at approximately the frequency average of the corresponding signals in the 158 K ^13^C{^1^H} SSNMR spectrum, which we suggest reflects the low-temperature limiting structure. The ^1^H NMR spectrum displays a single alkene environment (2 H relative integral) at *δ* 5.08, and an integral 6 H high field signal at *δ* 0.56. A ^31^P/^1^H HMBC experiment shows that this high field signal correlates strongly with the ^31^P environment that shows the large coupling with ^103^Rh; and DEPT experiments indicate it to be a CH_3_ group. These data suggest time-averaged *C*_s_ symmetry at 193 K in solution. On warming rapid decomposition starts that eventually forms **[1-(butadiene)][BAr^F^_4_]** in *ca.* 50% yield alongside other uncharacterized products,^111^ that mean we have not been able to study this process at higher temperatures in solution.

**Fig. 7:**
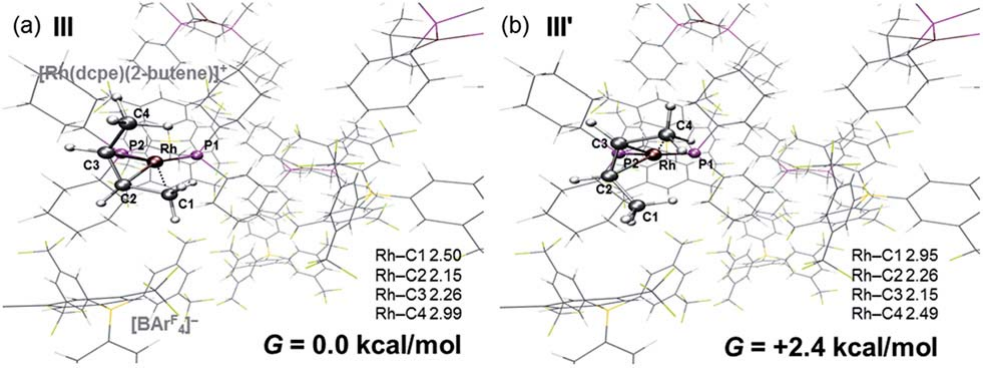
Computed structures of (a) **III** and (b) **III′** in the solid-state with selected distances in Å.

DFT calculations have explored the behavior of the **[1-(butene)]^+^** cation in the solid-state (see Scheme 12). Starting from the *cis*-2-butene isomer (**III**) oxidative cleavage of the agostic C1–H bond accesses an allyl-hydride species (**IV**) at +8.3 kcal mol^–1^*via* a transition state at 14.5 kcal mol^–1^. Both values are *ca.* 5 kcal mol^–1^ higher than the equivalent process with the propene analogue. Reductive coupling with the distal C3 carbon is no longer a near-degenerate process, but rather forms the 1-butene isomer (**V**) at +5.7 kcal mol^–1^. An adduct such as **V** is presumably initially formed in the reaction of **[1-NBA][BAr^F^_4_]** with 1-butene, however, the calculations suggest this would readily isomerize to the more stable *cis*-2-butene form with an overall barrier of only 8.8 kcal mol^–1^. The calculations also indicate that **V** should be kinetically accessible at room temperature. Assessment of the energy of the **[1-(*trans*-2-butene)]^+^** cation within the solid-state lattice indicates it would lie 11.4 kcal mol^–1^ above **III**. This large energy difference again reflects the environment imposed by the solid-state lattice, as calculations on the isolated cations indicate they lie within 0.3 kcal mol^–1^ of each other. The calculations also define a libration of the *cis*-2-butene ligand in **III** that serves to interchange the source of the agostic interaction *trans* to P2, from the C1–H1 bond in **III** to the C4–H4 bond in **III′** (see Fig. 7). This process occurs with a computed barrier of 3.0 kcal mol^–1^ and would account for the fluxionality observed in the SSNMR spectra, and the 193 K solution NMR spectra. Further rotation of the butene moiety produces a structure equivalent to the second component in the X-ray structure (*i.e.* based on positions C1′–C4′, Fig. 5). This second form has a computed energy of –0.4 kcal mol^–1^ and is accessible *via* an overall barrier of 22.7 kcal mol^–1^.

These combined experimental and computational data suggest a low temperature limiting structure for **[1-(butene)][BAr^F^_4_]** that has 2-butene bound in the *cis*-form with a supporting agostic interaction from the methyl group (Scheme 12). In solution at 193 K a low energy libration of the 2-butene ligand provides time-averaged *C*_s_ symmetry by exchanging the agostic methyl groups (*i.e.* C1 and C4, Fig. 7). This is slowed in the solid-state at 158 K. On warming in both solution and the solid-state there is evidence for further fluxional processes occurring. While the NMR data do not allow us to discriminate between a simple full rotation of the alkene fragment or a reversible C–H activation to give an allyl-hydride, the calculations suggest that the latter is more accessible with a barrier of 14.5 kcal mol^–1^ (Scheme 12) compared to 22.5 kcal mol^–1^ for *C*_2_ rotation.

**Scheme 13:**
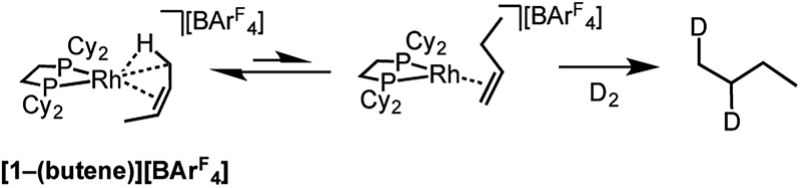
Addition of D_2_ to **[1-(butene)][BAr^F^_4_]**.

Further evidence for the isomerization process in Scheme 12 being accessible in the solid-state comes from addition of D_2_ to **[1-(butene)][BAr^F^_4_]**, which is shown to have *cis*-2-butene bound, but forms 1,2-d_2_-butane as the condensable volatile product: the product of D_2_ addition to 1-butene (Scheme 13). This suggests that isomerization from 2-butene to 1-butene is fast (*i.e.*Scheme 12) and that hydrogenation of the terminal alkene is significantly faster than the internal, a well-known observation for cationic Rh-based catalysts in solution.^112^

### 2.5 Catalytic 1-butene isomerization: solid-state molecular organometallic catalysis (SMOM-Cat)

**Fig. 8:**
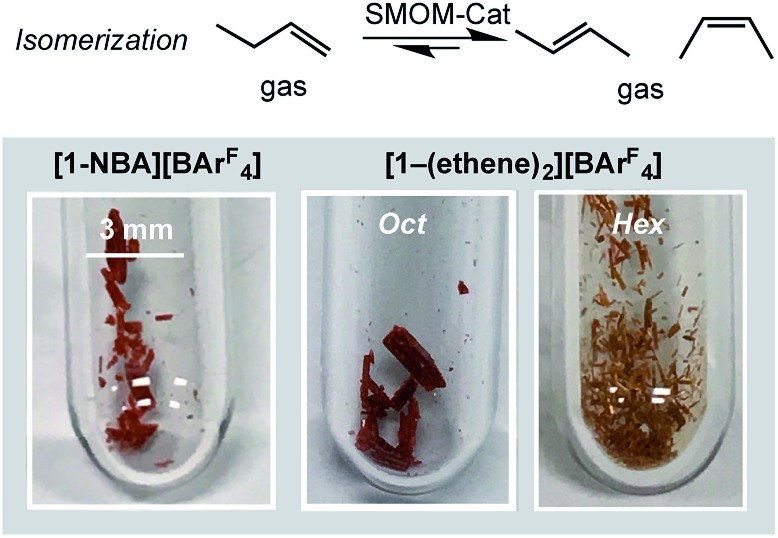
Representative examples of the physical forms of the various crystalline catalysts used for the gas/solid isomerization of 1-butene to *trans* and *cis*-2-butene. SMOM-Cat = solid-state molecular organometallic catalysis.

The fast H/D exchange observed for **[1-(propene)][BAr^F^_4_]**, and isomerization of 1-butene to 2-butene in **[1-(butene)][BAr^F^_4_]**, suggested that the systems described herein would make good alkene isomerization catalysts. The complexes **[1-NBA][BAr^F^_4_]**, **[1-(ethene)_2_][BAr^F^_4_]-Oct**, and **[1-(ethene)_2_][BAr^F^_4_]-Hex** were thus screened (but conditions not optimized) in the isomerization of 1-butene to 2-butene in solid/gas catalysis. Crystals of approximate edge length ∼0.2 mm were used for all (Fig. 8). We did not explicitly grade the samples, in the main due to the sensitivity of **[1-(ethene)_2_][BAr^F^_4_]-Hex**, and so the catalytic data presented should be viewed as indicative of the overall rate of isomerization rather than an absolute measure. This was performed on a small, but convenient, scale as we have described previously,^63^ by taking a thick-walled NMR tube of volume 2.05 cm^3^ fitted with Teflon stopcock that allows for the addition of gases, adding a crystalline sample of catalyst (2.5 mg, ∼1.7 μmol), brief evacuation, refilling with 1-butene gas (1 atm, 86 μmol^113^) and analysis by gas-phase ^1^H NMR spectroscopy. This loading, assuming all sites in the crystalline material have the same activity, gives TON_(bulk)_ of ∼51 for 100% conversion. This represents a minimum TON, as if only the most accessible sites, or those nearest to the surface, were kinetically competent then the actual number of active sites would be lower. The catalysts yield close to the thermodynamic equilibrium mixture of 1-butene : 2-butene of ∼3 : 97,^14^,^114^,^115^ in a *cis* : *trans* ratio of 1 : 2 as measured by gas-phase infra-red and ^1^H NMR spectroscopy (CDCl_3_) of the dissolved gas.^110^

**Fig. 9:**
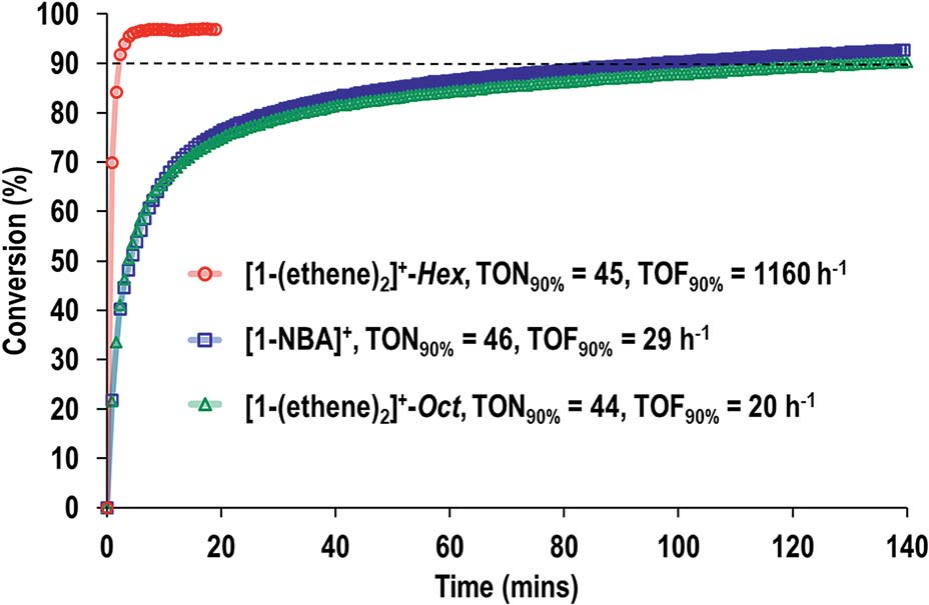
Comparison of SMOM-cat in the isomerization of 1-butene to 2-butene as measured by gas phase ^1^H NMR spectroscopy. All catalysts = ∼2.5 mg sample (∼1.7 μmol). 1-Butene = 1 atm (86 μmol at 298 K). Dashed line indicates 90% conversion.

Fig. 9 shows a time/conversion behavior for these three catalyst systems. **[1-(Ethene)_2_][BAr^F^_4_]-Hex** is by far the fastest catalyst, the system essentially reaching equilibrium (∼97% conversion) after 6 minutes. 90% conversion is reached after 2.3 min, TOF(90%) = 1160 h^–1^**[1-NBA][BAr^F^_4_]** and **[1-(ethene)_2_][BAr^F^_4_]-Oct**, are slower, TOF(90%) = 29, 20 h^–1^ respectively taking 1.5 and 2 hours to reach 90% conversion. This demonstrates a significant structure/activity relationship, with the porous **[1-(ethene)_2_][BAr^F^_4_]-Hex** operating as a much faster catalyst than its non-porous polymorph. To probe the influence of surface area finely crushed samples were prepared for which the surface area would be expected to be significantly greater.^116^ All of these crushed samples were significantly faster than for the larger crystalline samples, *e.g.* TOF(95%) = 3100 h^–1^ for **[1-(ethene)_2_][BAr^F^_4_]-Hex** (ESI†). The effects of the porous structure are not evident with the finely crushed samples, and for practical purposes the three catalysts operate with the same efficiency.

**Fig. 10:**
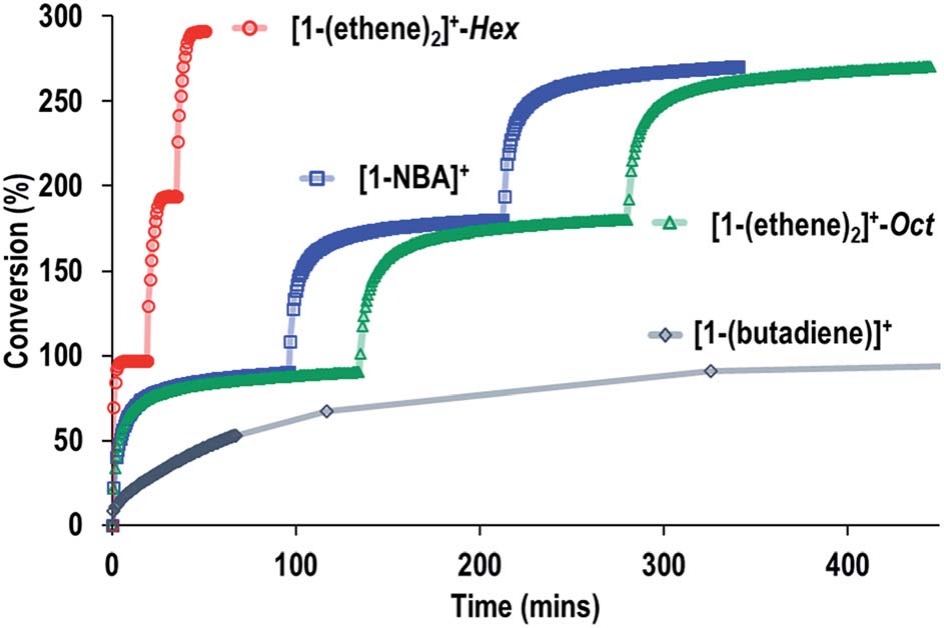
Comparison of recycling of the SMOM-cat in the isomerization of 1-butene to 2-butene as measured by gas phase NMR spectroscopy. Conditions as Fig. 9.

The SMOM-Cat can all be recycled, and Fig. 10 shows time/conversion plots for 3 recharge events, when fresh 1-butene is added immediately after greater than 90% conversion has been achieved by brief exposure to vacuum (30 s, 10^–3^ mbar) and refilling. For **[1-(ethene)_2_][BAr^F^_4_]-Oct** and **[1-NBA][BAr^F^_4_]** very similar overall temporal profiles were observed compared to the first addition of 1-butene (*cf.*Fig. 9). For **[1-(ethene)_2_][BAr^F^_4_]-Hex** some activity is lost, so that TOF is reduced to ∼450 h^–1^. We suggest this is due to the partial collapse of the lattice under vacuum during the recycling protocol. For both **[1-(ethene)_2_][BAr^F^_4_]-Oct** and **[1-(ethene)_2_][BAr^F^_4_]-Hex** ten charging cycles have been performed for 1-butene isomerization, with no appreciable drop in conversion between the first and last recharges. If samples are aged for 48 hours in the solid-state under 1-butene, conversion to **[1-(butadiene)][BAr^F^_4_]** occurs, as described in Section 2.1. This results in a significantly attenuated catalytic activity and only very slow conversion is subsequently obtained (90% conversion, 5.5 hours, TOF = 8 h^–1^), Fig. 10.

We have previously shown that addition of CO_(g)_ to crystalline samples of [Rh(^i^Bu_2_PCH_2_CH_2_P^i^Bu_2_)(η^2^η^2^-C_4_H_6_)][BAr^F^_4_] is slow enough (days) to form a catalytically inactive, passivated, layer of [Rh(^i^Bu_2_PCH_2_CH_2_P^i^Bu_2_)(CO)_2_][BAr^F^_4_] after a few hours in the resulting crystalline material.^63^ This allows for the activity of surface sites to be probed in the hydrogenation of ethene, which were suggested to be considerably more active compared to the bulk. This approach was inspired by the work of Brookhart on single-crystal solid/gas catalysis using Ir(POCOP)(C_2_H_4_).^51^ For **[1-NBA][BAr^F^_4_]** reaction with CO (1 atm) is much faster, forming [(Cy_2_PCH_2_CH_2_PCy_2_)Rh(CO)_2_][BAr^F^_4_] in 25% conversion after only 10 seconds as measured by ^31^P{^1^H} spectroscopy of the dissolved solid. At the same time considerable cracking of the crystals also occurred as indicated by optical microscopy, that likely exposes the interior of the crystals (see ESI† for full details).^117^ This means that passivation of just the surface sites is problematic and we have not pursued this approach further with these samples. However, that bulk crystalline samples show a significantly lower TOF compared to more finely-divided crushed samples, and that porous **[1-(ethene)_2_][BAr^F^_4_]-Hex** is particularly active, suggests that the most active catalyst sites sit at, or near, the surface or an open pore. On the basis of the synthetic studies (Section 2.4) we propose that **[1-(butene)][BAr^F^_4_]** is likely the resting state during catalysis.

Although catalyzed double bond isomerizations in alkenes are common, those involving 1-butene and well-defined transition metal catalysts are less well represented. A notable homogenous example is Ni(η^6^-C_6_H_5_CH_3_)(SiCl_3_)_2_ that rapidly isomerizes 1-butene to 2-butene at 0 °C in bromobenzene, at loadings as low as 0.1 mol% (TOF ∼8600 h^–1^).^13^ Although the long term stability and recyclability was not commented upon, in other solvents significant decomposition was noted. Other homogenous systems are known,^14^,^114^,^118^ as are heterogeneous systems that operate at room temperature.^119^,^120^ However, we believe that catalysts such as **[1-(ethene)_2_][BAr^F^_4_]-Hex** are the first well-defined molecular systems that operate at 298 K under, industrially appealing, solid/gas conditions. In addition, they offer fine control of the spatial environment in the solid-state (*i.e.* show structure/activity relationships), show TOF_(min)_ that are competitive with the fast homogenous systems, and, moreover are recyclable. Although the solid/gas catalysts Ir(PCP^iPr^)(C_2_H_4_)^45^,^46^ or [Rh(PPh_3_)_2_(CO)]_3_[PW_12_O_40_]^47^–^49^ promote the isomerization of alkenes, higher temperatures are reported for the former (125 °C) while the latter is ill-defined on the molecular level and also not particularly active. A MOF-supported {Ir(ethene)_2_} fragment has been reported to dimerize ethene to butene, for which a mixture of isomers is reported but no further details were given regarding the isomerization process.^82^

### 2.6 Transfer dehydrogenation of butane to 2-butene

**Scheme 14:**
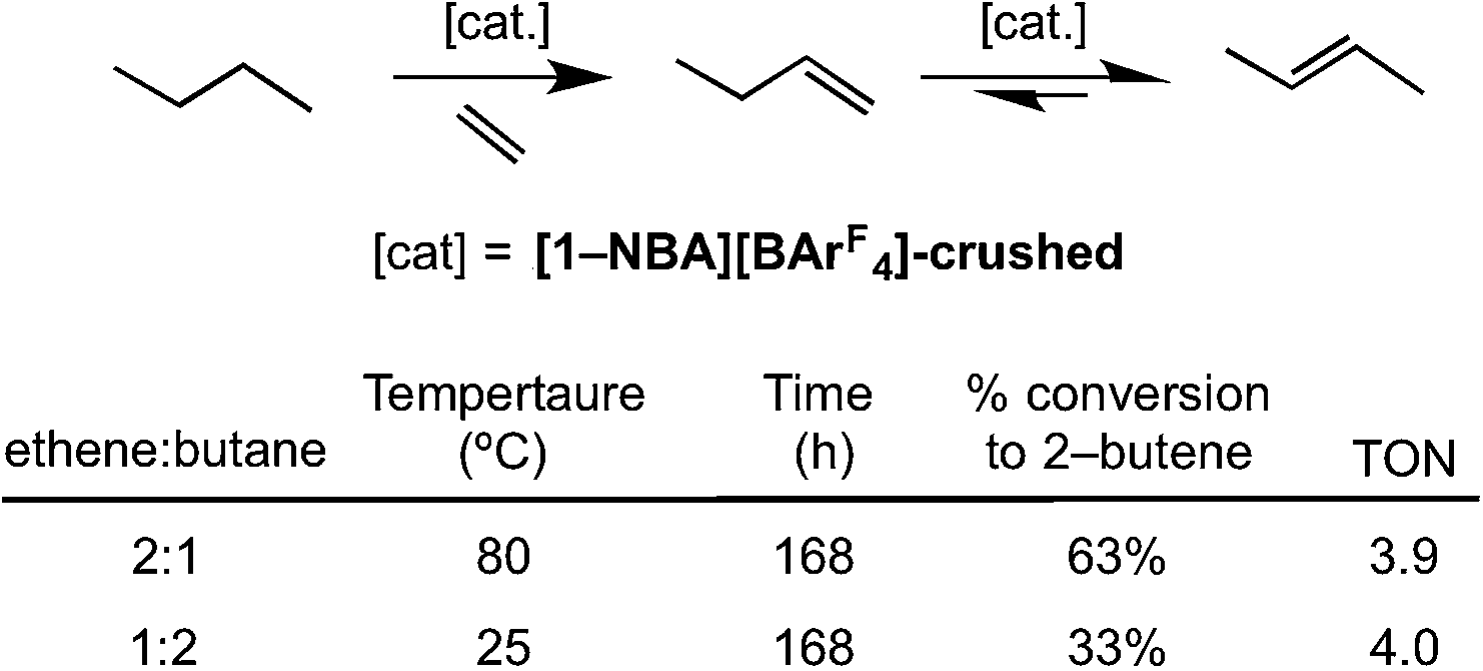
Transfer dehydrogenation of butene using sacrificial ethene. Conditions: total gas pressure butane 1 atm.

The ability of **[1-NBA][BAr^F^_4_]** to mediate the gas/solid transfer dehydrogenation of butane to butenes has been briefly explored (Scheme 14), monitored by gas-phase NMR spectroscopy as measured by the temporal profiles of butane and butene. As for the isomerization catalysis, a thick walled NMR tube was charged with finely-crushed crystalline **[1-NBA][BAr^F^_4_]** (∼6 mg, 4.1 μmol) and ethene and butane were admitted to the tube in a 1 : 2 ratio (total gas pressure 1 atm, 51 μmol butane). Periodic monitoring of the head space in the NMR tube showed that slow transfer dehydrogenation was occurring to form 2-butene, presumably by slow dehydrogenation to form 1-butene (not observed) and rapid isomerization. After 168 h at 298 K there was a 33% conversion, which equates to ∼4 turnovers. The catalysis was also shown to operate at 80 °C with an excess of ethene (2 : 1), under which conditions 68% conversion of butane to butenes is observed (TON = 4). Under these conditions the crystals lost definition and became wax-like. Although these turnover numbers are considerably smaller those reported for the best solid-phase molecular catalyst Ir(PCP^iPr^)(C_2_H_4_) in the pentane/propene system at 240 °C (*e.g.* TON greater than 1000), or related well-defined silica supported catalysts,^44^ the observation of any catalytic activity at 298 K for this challenging reaction is encouraging. As far as we are aware this is the first time solid/gas transfer dehydrogenation has been reported using a well-defined molecular catalyst at room temperature and low pressures. The resting state observed in the bulk during dehydrogenation catalysis is **[1-(ethene)_2_][BAr^F^_4_]**. We cannot discount that a parallel slow ethene dimerization reaction also occurs under these conditions (Section 2.1).

## 3. Conclusions

Solid/gas organometallic chemistry performed on well-defined molecular species offers opportunities in synthesis and catalysis that complement solution routes.^41^ We show here that the rhodium sigma-alkane complex **[1-NBA][BAr^F^_4_]** (that is prepared by solid/gas routes itself) acts as a synthetic starting point for a variety of reactive and unusual alkene complexes that are challenging to prepare pure by solution routes. The alkane thus acts as “token” ligand,^121^ and it is the unique environment provided by the solid-state packing of anions that allows for such single-crystal to single-crystal transformation to occur at such reactive but well-defined molecular cations. When experimental observations are combined with periodic DFT and chemical shift calculations a very complete picture of structure, mobility and reactivity in the solid-state can be formed, as we have also recently demonstrated in **[1-NBA][BAr^F^_4_]** and other systems.^58^,^59^,^62^ Such solid-state molecular organometallic systems thus provide a platform for synthesis and characterization equal in many ways to solution. Further demonstration of this comes from deployment of some of these systems in solid/gas butene isomerization and butane transfer dehydrogenation catalysis that shows catalysis in such an environment is certainly possible, and even rather efficient in some cases. Moreover, correlations between the solid-state structure (*i.e.* porosity) and high turnover frequency can be made. These results suggest that, more generally, solid-state molecular organometallic systems may offer opportunities and advantages in synthesis and catalysis and thus should be considered as a valuable additional approach to be used in the armoury of the organometallic chemist. That a relatively stable sigma-alkane complex (albeit not a methane, or related complex, which have been observed at very low temperatures in solution^122^,^123^) acts as a precursor to this chemistry in the solid-state evokes Chatts prediction in 1976: “I believe that in twenty-five years methane will be the most popular ligand in coordination chemistry”.^124^,^125^ It will be interesting to see if sigma-alkane complexes similar to those described here, more generally, are versatile enough to realize this prediction using solid/gas chemistry.

## Supplementary Material

Supplementary information

Supplementary information

Crystal structure data
