# Supplementary material for: Solid-state molecular organometallic chemistry. Single-crystal to single-crystal reactivity and catalysis with light hydrocarbon substrates
Source: Chem Sci. 2017 Jul 6;8(9):6014–29. doi: 10.1039/c7sc01491k (PMC5625289; doi:10.1039/c7sc01491k)
Supplement: Supplementary file 2 [file SC-008-C7SC01491K-s002.pdf]

Electronic Supplementary Material (ESI) for Chemical Science.
This journal is © The Royal Society of Chemistry 2017

Supporting Information

Solid-State Molecular Organometallic Chemistry. Single-Crystal to Single-Crystal Reactivity and Catalysis with Light Hydrocarbon Substrates.

F. Mark Chadwick, Alasdair I. McKay, Antonio J. Martinez-Martinez, Nicholas H. Rees, Tobias Krämer, Stuart A. Macgregor* and Andrew S. Weller*

Department of Chemistry, Chemistry Research Laboratories, University of Oxford, OX1 3TA, U.K. Email: Andrew.Weller@Chem.ox.ac.uk

Institute of Chemical Sciences, Heriot-Watt University, Edinburgh, EH14 4AS. UK. Email: S.A.Macgregor@hw.ac.uk

TABLE OF CONTENTS

S.1. EXPERIMENTAL DETAILS **4**
S.1.1 General methods 4

S.2. SYNTHETIC PROCEDURES **7**
S.2.1. Synthesis and characterization of [1-(ethene)$_2$][BAr$^F_4$] 7
S.2.1.1. Attempted solution phase synthesis from [1-F$_2$C$_6$H$_4$][BAr$^F_4$] *7*
S.2.1.2. Solid-state synthesis of [1-(ethene)$_2$][BAr$^F_4$]-Oct. *7*
S.2.1.3. Synthesis of [1-(ethene)$_2$][BAr$^F_4$]-Hex *8*
S.2.1.4. Characterizing data for [1-(ethene)$_2$][BAr$^F_4$]-Oct and [1-(ethene)$_2$][BAr$^F_4$]-Hex *8*
S.2.1.5. Spectra of [1-(ethene)$_2$][BAr$^F_4$]-Oct and [1-(ethene)$_2$][BAr$^F_4$]-Hex *10*
S.2.2. Synthesis and characterization of [1-(propene)][BAr$^F_4$] and [1-(propene)$_2$][BAr$^F_4$] 18
S.2.2.1. Attempted solution phase synthesis from [1-F$_2$C$_6$H$_4$][BAr$^F_4$] *18*
S.2.2.2. Solid state synthesis and characterization of [1-(propene)][BAr$^F_4$] *19*
S.2.2.3. Spectra of [1-(propene)][BAr$^F_4$] *21*
S.2.2.4. Solution synthesis and characterization of [1-(propene)$_2$][BAr$^F_4$] *30*
S.2.2.5. H/D scrambling studies for [1-(d$_3$-propene)][BAr$^F_4$] *31*
S.2.3. Synthesis and characterization of [1-(butene)][BAr$^F_4$] 34
S.2.3.1. Attempted solution phase synthesis from [1-F$_2$C$_6$H$_4$][BAr$^F_4$] *34*
S.2.3.2. Solid-state synthesis and characterization of [1-(butene)][BAr$^F_4$] *34*
S.2.3.3. Spectra of [1-(butene)][BAr$^F_4$] *36*
S.2.3.4. Addition of D$_2$ to [1-(butene)][BAr$^F_4$] *41*
S.2.3.5. Characterization of the non-volatile component [Rh(dcpe)(CD$_3$CN)$_2$][BAr$^F_4$] *42*
S.2.4. Synthesis and characterization of [1-(butadiene)][BAr$^F_4$] 43
S.2.4.1. Solution synthesis of [1-(butadiene)][BAr$^F_4$] *43*
S.2.4.2. Solid state synthesis and characterization of [1-(butadiene)][BAr$^F_4$] *43*
S.2.4.3. Spectra of [1-(butadiene)][BAr$^F_4$] *44*
S.2.5. Characterization of [1-(CO)$_2$][BAr$^F_4$] 47

S.3. CRYSTALLOGRAPHIC AND REFINEMENT DATA **48**
S.3.1. Crystal structure determinations 48
S.3.2. Selected crystallographic and refinement data 49
S.3.3. Crystal structure of [1-(propene)$_2$][BAr$^F_4$] 51
S.3.4. Additional details on the crystal structures 52

S.4. SOLID STATE MOLECULAR ORGANOMETALLIC CATALYST (SMOM-CAT) DATA **53**
S.4.1. Catalytic isomerization experiments of 1-butene to 2-butene 53
S.4.1.1. General experimental considerations for the gas phase catalytic isomerization of 1-butene to 2-butene *53*
S.4.1.2. Isomerization catalytic data for [1-NBA][BAr$^F_4$]-large *54*
S.4.1.3. Isomerization catalytic data for [1-(ethene)$_2$][BAr$^F_4$]-Oct-large *55*
S.4.1.4. Isomerization catalytic data for [1-(ethene)$_2$][BAr$^F_4$]-Hex-large *56*
S.4.1.5. Isomerization catalytic data for [1-NBA][BAr$^F_4$]-crushed *57*
S.4.1.6. Isomerization catalytic data for [1-(ethene)$_2$][BAr$^F_4$]-Oct-crushed *58*
S.4.1.7. Isomerization catalytic data for [1-(ethene)$_2$][BAr$^F_4$]-Hex-crushed *59*
S.4.2. Catalyst recyclability for the gas phase isomerization of 1-butene to 2-butene 60

S.4.2.1. General experimental considerations for the catalyst recycling studies 60
S.4.2.1.1. Catalyst recycling studies for [1-NBA][BAr$^F{}_4$]-large 60
S.4.2.1.2. Catalyst recycling studies for [1-(ethene)$_2$][BAr$^F{}_4$]-Oct-large 61
S.4.2.1.3. Catalyst recycling studies for [1-(ethene)$_2$][BAr$^F{}_4$]-Hex-large 62
S.4.2.1.4. Catalyst recycling studies for [1-NBA][BAr$^F{}_4$]-crushed 63
S.4.2.1.5. Catalyst recycling studies for [1-(ethene)$_2$][BAr$^F{}_4$]-Oct-crushed 64
S.4.2.1.6. Catalyst recycling studies for [1-(ethene)$_2$][BAr$^F{}_4$]-Hex-crushed 65
S.4.2.1.7. Comparison of TON and TOF for the various SMOM-catalysts 66
S.4.2.2. Surface *versus* bulk catalytic studies 68
S.4.2.2.1. Surface passivation studies 68
S.4.2.2.2. Solid state NMR study 69
S.4.3. Catalytic transfer dehydrogenation of butane to 2-butene 69
S.4.3.1. Experimental details for the catalytic transfer dehydrogenation 69
S.4.4. Oligomerization of ethene 70

S.5. COMPUTATIONAL STUDIES 72
S.5.1. Computational methods 72
S.5.2. Optimized geometries 75
S.5.3. Calculated ^{1}H/^{13}C NMR chemical shifts 77
S.5.4. Mechanisms 80

S.6. REFERENCES 90

S.1. Experimental Details

S.1.1 General methods

All manipulations (unless otherwise stated) were performed under an atmosphere of argon, using standard Schlenk techniques on a dual vacuum/inlet manifold or by employment of an MBraun glovebox. Glassware was dried in an oven at 130°C overnight prior to use. Pentane, hexane and CH_2Cl_2 were dried using an MBraun SPS-800 solvent purification system and degassed by three freeze-pump-thaw cycles. CD_2Cl_2 and was dried by stirring over CaH_2 overnight before being vacuum distilled and subsequently degassed by three freeze-pump-thaw cycles. 1,2-$F_2C_6H_4$ (abbreviated as $F_2C_6H_4$) was stirred over Al_2O_3 for two hours then over CaH_2 overnight before being vacuum distilled and subsequently degassed by three freeze-pump-thaw cycles. Ethene, propene, 1-butene and butane were all supplied by CK gases and were used as received. 3,3,3-d_3-propene was supplied by Cambridge Isotopes or QMX, and used as received.

Solution NMR data were collected on either a Bruker AVD 500 MHz or a Bruker Ascend 400 MHz spectrometer at room temperature unless otherwise started. Non-deuterated solvents were locked to standard external CD_2Cl_2 solutions. Residual protio solvent resonances were used as a reference for ^{1}H NMR spectra. A small amount of CD_2Cl_2 was added as a reference for ^{2}H{^{1}H} NMR spectra. ^{31}P{^{1}H} NMR spectra were referenced externally to 85% H_3PO_4 (D_2O). All chemical shifts (δ) are quoted in ppm and coupling constants in Hz.

Solid state NMR (SSNMR) samples were prepared by pre-loading in an argon-filled glove box either 70 or 35 mg of the corresponding microcrystalline sample into a 4 or 3.2 mm zirconia solid state rotor, respectively.

In-situ SSNMR samples were prepared as follows. A 4 mm zirconia rotor was filled with 70 mg of powdered microcrystalline sample of **[1-NBD][BAr^F$_4$]**,[S1] ground using the back of a spatula, in an argon-filled glovebox before sealing in a J. Young's flask. Outside the glovebox this sample was evacuated and subjected to react with H_2 (30 psi, 4 hours) to form **[1-NBA][BAr^F$_4$]** *in-situ*.[S1] This was then capped off and shown by ^{31}P{^{1}H} SSNMR to have gone to completion before taking the sample back into the glovebox, removing the cap, and loading into a custom built piece of glassware that allows capping off under a flush of reagent gas (Figure S1). The samples would then be exposed and reacted with the desired gas, capped off, and measured immediately. It was noted that the reaction times appear to take longer in the SSNMR rotor, either due to lower surface area or the highly compacted nature of the sample.

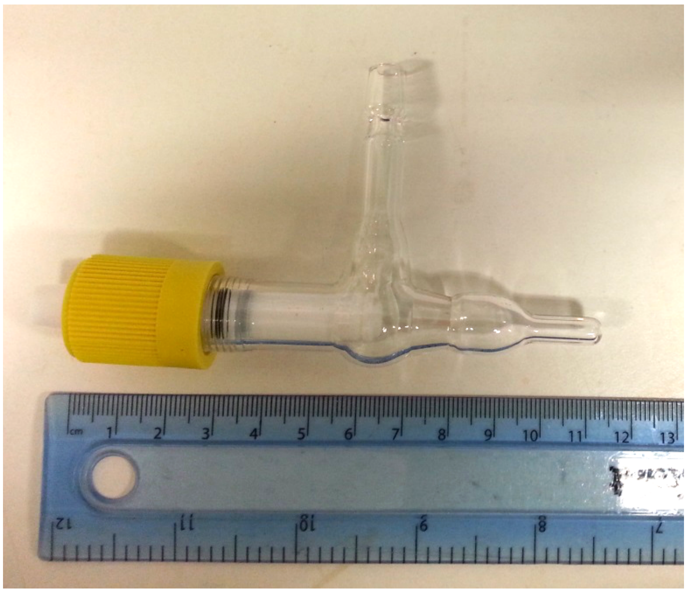

Figure S1: Custom built glassware for capping a solid state NMR rotor under a flush of reagent gas.

Ex-situ SSNMR samples were prepared by adding alkene (15 psi, 298 K) to a finely powdered *in-situ* prepared sample of **[1-NBA][BAr$^F_4$]**[S1] (c. 70 mg) in a J. Young's flask (c. 50 mL). After 2 hours at room temperature the flask was then evacuated (1 × 10^{-2} mbar). The sample was then loaded into a 4 mm rotor and capped off in an argon filled glovebox.

Solid state NMR spectra were obtained on a Bruker Avance III HD spectrometer equipped with a 9.4 Tesla magnet, operating at 100.6 MHz for ^{13}C and 162 MHz for ^{31}P using either a 4 or 3.2 mm zirconia rotors containing approximately 70 and 35 mg of sample, respectively, and a MAS rate of 10 kHz. For ^{13}C CP/MAS a sequence with a variable X-amplitude spin-lock pulse[S2] and spinal64 proton decoupling was used. 4500 transients were acquired using a contact time of 2.5 ms, an acquisition time of 25 ms (2048 data points zero filled to 32 K) and a recycle delay of 2 s. All ^{13}C spectra were referenced to adamantane (the upfield methine resonance was taken to be at δ = 29.5 ppm[S3] on a scale where δ(TMS) = 0 ppm as a secondary reference.

Gas phase ^{1}H NMR spectroscopy was carried out using a Bruker Ascend 400 MHz spectrometer. Chemical shifts are referenced relative to reported gas phase data.[S4] The T1 delay was set to 1 s, and this has been previously shown to allow for the accurate comparison of integrals.[S4] Samples were loaded into a high-pressure NMR tube sealed with a Teflon stopcock, before being transferred to a Schlenk vacuum line, evacuated and then loaded with the gaseous reagents (via a custom made glass T-piece adaptor, Figure S2). The spectrometer was locked and shimmed to a separate C_6D_6 sample in a similar bore tube, the sample was then replaced and spectra run. For isomerization catalytic runs the machine was locked and shimmed

before the gaseous reagents were added (full experimental details of isomerization catalysis are given in Section S.4.).

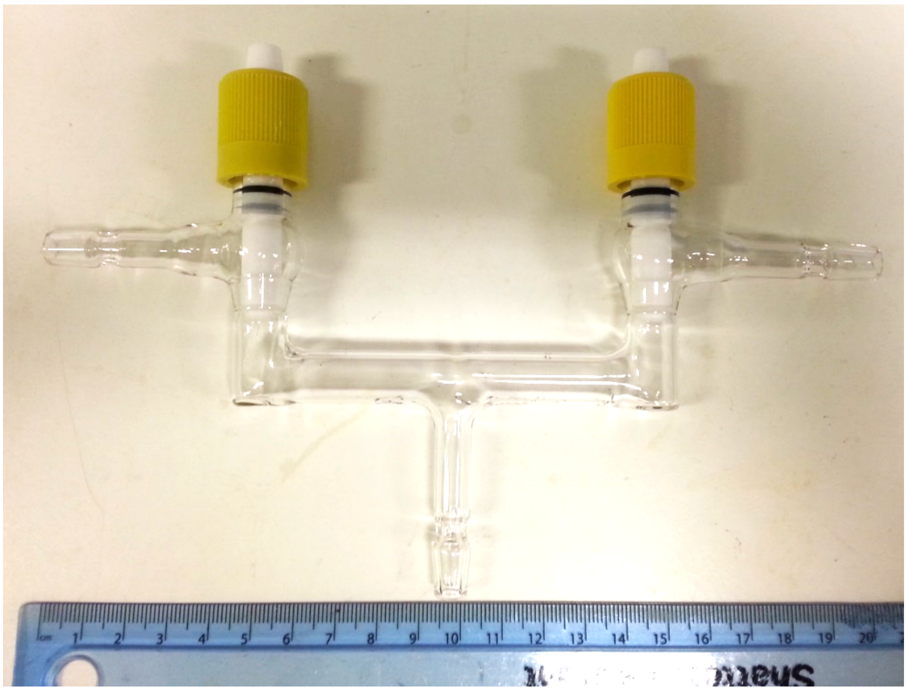

Figure S2: Custom made glass T-piece adaptor for manipulation of reagent gases.

Electrospray ionization mass spectrometry (ESI-MS) was carried out using a Bruker MicrOTOF instrument directly connected to a modified Innovative Technology glovebox.[S5] Typical acquisition parameters were used (sample flow rate: 4 µL min^{-1}, nebulizer gas pressure: 0.4 bar, drying gas: Argon at 333 K flowing at 4 L min^{-1}, capillary voltage: 4.5 kV, exit voltage: 60 V). The spectrometer was calibrated using a mixture of tetraalkyl ammonium bromides $[N(C_nH_{2n+1})_4]Br$ (n = 2-8, 12, 16 and 18). Samples were diluted to a concentration of 1×10^{-6} M in the appropriate solvent before sampling by ESI-MS.

Gaseous IR spectra were collected on a Bruker Vertex 80 spectrometer using a Specac gas sampling sample cell. Solid state and solution IR spectra were collected on a Bruker Tensor 27 spectrometer.

$[(Cy_2PCH_2CH_2PCy_2)Rh(\eta^2:\eta^2\text{-}C_7H_8)][BAr^F_4]$ (**[1-NBD][BAr$^F_4$]**),[S1] $[(Cy_2PCH_2CH_2PCy_2)Rh(\eta^2:\eta^2\text{-}C_7H_{12})][BAr^F_4]$ (**[1-NBA][BAr$^F_4$]**)[S1] and $[(Cy_2PCH_2CH_2PCy_2)Rh(\eta^6\text{-}F_2C_6H_4)][BAr^F_4]$ (**[1-F$_2$C$_6$H$_4$][BAr$^F_4$]**[S6] were prepared by literature procedures.

S.2. Synthetic procedures

S.2.1. Synthesis and characterization of [1-(ethene)$_2$][BAr$^F{}_4$]

S.2.1.1. Attempted solution phase synthesis from [1-$F_2C_6H_4$][BAr$^F{}_4$]

A crystalline sample of **[1-$F_2C_6H_4$][BAr$^F{}_4$]** (25 mg, 0.0166 mmol) was taken up in CD_2Cl_2 (0.5 mL) in a high pressure NMR tube. This was freeze-pump-thaw degassed three times (< 1 × 10^{-2} mbar) before ethene gas (15 psi, 298 K) was added. An immediate darkening of the yellow solution to orange occurred. $^{31}P\{^1H\}$ NMR spectroscopy indicated that near quantitative conversion to **[1-(ethene)$_2$][BAr$^F{}_4$]** had occurred after 15 minutes (Figure S3, bottom), however an amount of the starting **[1-$F_2C_6H_4$][BAr$^F{}_4$]** remains (labelled *), indicating it is in equilibrium with **[1-(ethene)$_2$][BAr$^F{}_4$]**. Any attempted work-up involving vacuum results in the complete decomposition of the species, to presumed solvent C-H or C-Cl bond activated products (indicated from ESI-MS showing the presence of chloride-bridged rhodium dimers, Figure S3, top). Furthermore leaving **[1-(ethene)$_2$][BAr$^F{}_4$]** complex in CH_2Cl_2 solution, at room temperature, also resulted in complete decomposition over a period of approximately an hour.

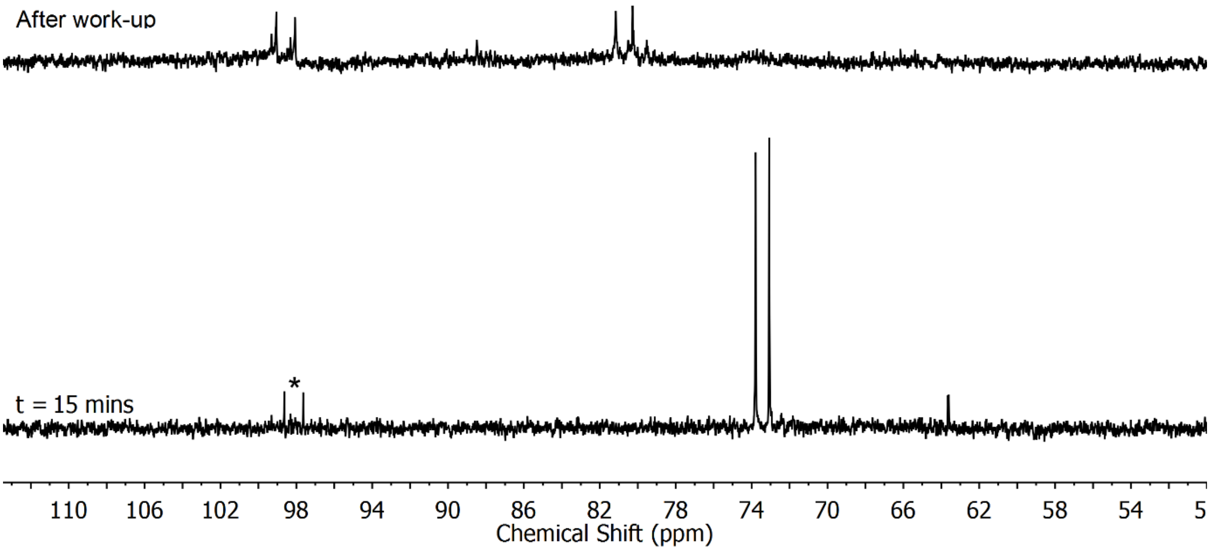

Figure S3: The $^{31}P\{^1H\}$ solution (CD_2Cl_2, 298 K, 202 MHz) NMR spectrum of the attempted solution phase synthesis of **[1-(ethene)$_2$][BAr$^F{}_4$]**. The bottom spectrum is measured after 15 min of reaction, the top spectrum is after attempted work up. The primary resonance in the bottom spectrum corresponds to **[1-(ethene)$_2$][BAr$^F{}_4$]** (*vide infra*). Both spectra were measured at 253 K to ensure sharp resonances, see Figures S6 and S7.

S.2.1.2. Solid-state synthesis of [1-(ethene)$_2$][BAr$^F{}_4$]-*Oct*.

To an orange sample of crystalline **[1-NBA][BAr$^F{}_4$]** (25 mg, 0.0168 mmol) in an evacuated (< 1 × 10^{-2} mbar) J. Young's flask (c. 50 mL) ethene gas (15 psi, 298 K) is added and left standing for 2 hours. Little color change is observed, though the

crystals take on the appearance of liquid on the surface assumed to be norbornane. It is not possible to remove the residual norbornane (attempts to do so by washing with pentane did not work), however the synthesis goes in > 95% yield by $^{31}P\{^{1}H\}$ SSNMR spectroscopy and $^{31}P\{^{1}H\}$ solution NMR spectroscopy when dissolved up in CD_2Cl_2 (the only other signal being due to an uncharacterized decomposition product, which mass spectroscopic evidence suggests a product resulting from CH_2Cl_2 activation). After 16 hours in CD_2Cl_2 the compound decomposes to a range of uncharacterized products. Dissolving the product in 1,2-$F_2C_6H_4$ results in the formation of **[1-$F_2C_6H_4$][BAr$^F_4$]**.[S6]

S.2.1.3. Synthesis of [1-(ethene)$_2$][BAr$^F_4$]-*Hex*

To an orange sample of crystalline **[1-NBA][BAr$^F_4$]** (120 mg, 80.8 µmol) in an evacuated (< 1 × 10^{-2} mbar) J Young's flask (c. 50 mL) ethene gas (15 psi, 298 K) is added and left standing for 2 hours, to form **[1-(ethene)$_2$][BAr$^F_4$]-*Oct***. After this time and working under an atmosphere of ethene (15 psi) and at -78°C in an acetone/dry ice bath, the sample is dissolved in CH_2Cl_2 (1.5 mL), quickly filtered *via* cannula and layered with pentane (10 mL) at the same temperature. Storing at -80°C for a week yields orange needle like crystals of **[1-(ethene)$_2$][BAr$^F_4$]-*Hex***. Single crystals, directly selected from the mother liquor at -78°C, are suitable for an X-ray diffraction study. The crystalline material is quickly separated from the mother liquor working under an atmosphere of ethene (15 psi) at -78°C in an acetone/dry ice bath and then dried under a flow of ethene for 5 mins at room temperature. The crystalline material is stored in a glove box in a freezer operating at -25°C. Isolated crystalline yield: 72 mg (49.8 µmol, 62%).

Note: CH_2Cl_2 and pentane used in this preparation were freeze-pump-thawed degassed three times and then saturated and stored with ethene gas (15 psi) prior to use.

S.2.1.4. Characterizing data for [1-(ethene)$_2$][BAr$^F_4$]-*Oct* and [1-(ethene)$_2$][BAr$^F_4$]-*Hex*

Whilst solution NMR data (CD_2Cl_2) corresponding to **[1-(ethene)$_2$][BAr$^F_4$]-*Oct*** and **[1-(ethene)$_2$][BAr$^F_4$]-*Hex*** are identical, their SSNMR data differs in agreement with their distinct crystallographic environments.

^{1}H solution NMR (CD_2Cl_2, 298 K, 400 MHz) δ: 7.72 (8H, s, o-BAr$^F_4$), 7.56 (4H, s, p-BAr$^F_4$), 4.43 (8H, v br, $v_{1/2}$ = 94 Hz, ethene), 2.0-1.0 ppm (52H, multiple overlapping aliphatic resonances). It is noted the overall integral of the aliphatic region is high, this is attributed to recalcitrant NBA in the lattice.

$^{31}P\{^{1}H\}$ solution NMR (CD_2Cl_2, 298 K, 162 MHz) δ: 73.7 (v. br, $v_{½}$ ≈ 500 Hz).

^{1}H solution NMR (CD_2Cl_2, 193 K, 400 MHz) δ: 7.71 (8H, s, o-BAr^F_4), 7.54 (4H, s, p-BAr^F_4), 4.15 (8H, s, ethene), 2.0-1.0 ppm (52H, multiple overlapping aliphatic resonances). It is noted the overall integral of the aliphatic region is high, this is attributed to recalcitrant NBA in the lattice.

^{31}P{^{1}H} solution NMR (CD_2Cl_2, 193 K, 162 MHz) δ: 73.6 (d, J_{RhP} = 145 Hz).

^{13}C{^{1}H} solution NMR (CD_2Cl_2, 193 K, 126 MHz) δ: 161.4 (1:1:1:1 q, J_{BC} = 50 Hz, ipso-ArC), 134.3 (ortho-ArCH), 128.3 (q, J_{CF} = 32 Hz, meta-ArC), 124.1 (q, J_{CF} = 272 Hz, CF_3), 117.2 (para-ArCH), 82.4 (s, ethene C=C), 35.7 (m, Cy-CH), 30.3 (s, Cy-CH_2), 29.2 (s, Cy-CH_2), 28.3 (s, Cy-CH_2), 28.1 (s, Cy-CH_2), 27.0 (m, PCH_2), 26.3 (s, Cy-CH_2), 25.4 (s, Cy-CH_2), 25.3 (s, Cy-CH_2), 19.8 (m, Cy-CH_2).

^{31}P{^{1}H} SSNMR (for [1-(ethene)$_2$][BAr^F_4]-*Oct*; 162 MHz, 298 K, 10 kHz spin rate) δ: 73.7 (br, $v_{½}$ ≈ 410 Hz).

^{13}C{^{1}H} SSNMR (for [1-(ethene)$_2$][BAr^F_4]-*Oct*; 101 MHz, 298 K, 10 kHz spin rate) δ: 164.0 (BAr^F_4), 134.7 (BAr^F_4), 130.4 (BAr^F_4), 125.3 (BAr^F_4), 117.2 (BAr^F_4), 82.3 (ethene), 40-15 (multiple overlapping aliphatic resonances).

^{31}P{^{1}H} SSNMR (for [1-(ethene)$_2$][BAr^F_4]-*Hex*; 162 MHz, 298 K, 10 kHz spin rate) δ: 74.8 (v br, $v_{½}$ ≈ 1.3 kHz).

^{13}C{^{1}H} SSNMR (for [1-(ethene)$_2$][BAr^F_4]-*Hex*; 101 MHz, 298 K, 10 kHz spin rate) δ: 162.8 (BAr^F_4), 133.4 (BAr^F_4), 129.3 (BAr^F_4), 123.7 (BAr^F_4), 116.0 (BAr^F_4), 75.1 (ethene), 40-15 (multiple overlapping aliphatic broad resonances).

Mass Spec found (calc.): 581.2189 (581.2907) – note: there is considerable presence of **[1-(butadiene)][BAr^F_4]** and decomposition product of formula *m/z* = $[\{(Cy_2PCH_2CH_2PCy_2)Rh\}Cl_2]^{2+}-H_2$. There is no evidence for **[1-(butadiene)][BAr^F_4]** in bulk samples so it is assumed to form *via* an *in-situ* ESI-MS process.

Elemental analysis found (calc.) (carried out with a sample of [1-(ethene)$_2$][BAr^F_4]-*Hex*): C 51.37% (51.51%), H 4.74% (4.63%). Repeated attempts to obtain satisfactory elemental analysis for **[1-(ethene)$_2$][BAr^F_4]-*Oct*** have not been successful. Analyses obtained are typically low in carbon and hydrogen and generally consistent with the loss of the coordinated ethene molecules. Representative elemental analysis: C 49.37%, H 4.56%. We attribute this to the high air and moisture sensitivity of the complex.

S.2.1.5. Spectra of [1-(ethene)$_2$][BAr$^F_4$]-*Oct* and [1-(ethene)$_2$][BAr$^F_4$]-*Hex*

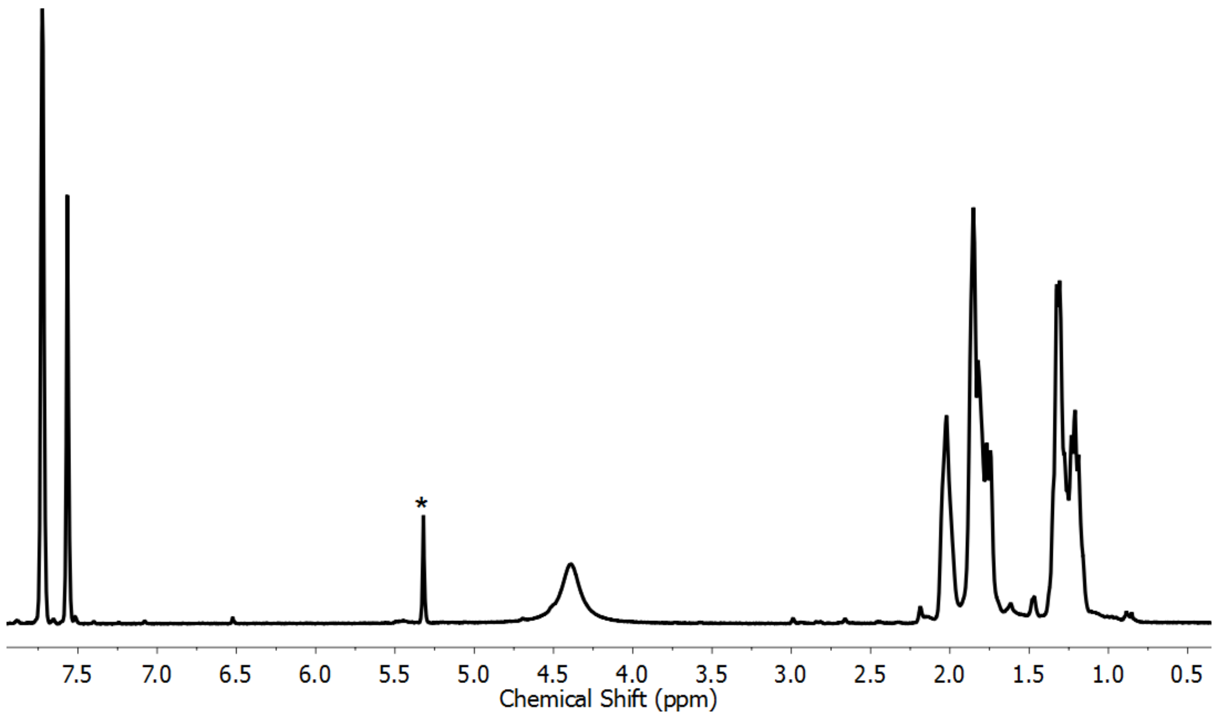

Figure S4: The solution ^{1}H NMR spectrum (CD_2Cl_2, 298 K, 400 MHz) of **[1-(ethene)$_2$][BAr$^F_4$]**, prepared by dissolving a crystalline sample of **[1-(ethene)$_2$][BAr$^F_4$]-*Oct*** which was synthesized by the solid/gas route. The resonance marked * is due to residual protio solvent.

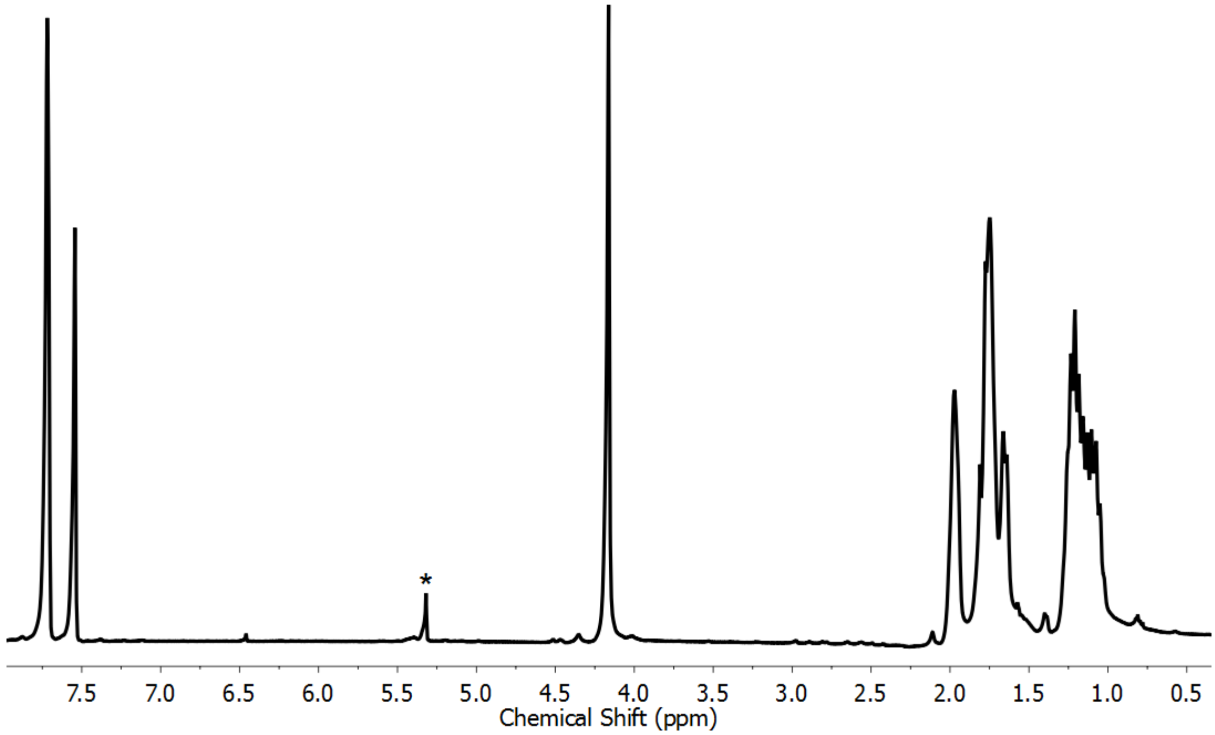

Figure S5: The solution ^{1}H NMR spectrum (CD_2Cl_2, 193 K, 400 MHz) of **[1-(ethene)$_2$][BAr$^F_4$]**, prepared by dissolving a crystalline sample of **[1-**

(ethene)$_2$][BAr^F$_4$]-*Oct* which was synthesized by the solid/gas route. The resonance marked * is due to residual protio solvent. The spectrum was meaured on the the same sample as figure S4.

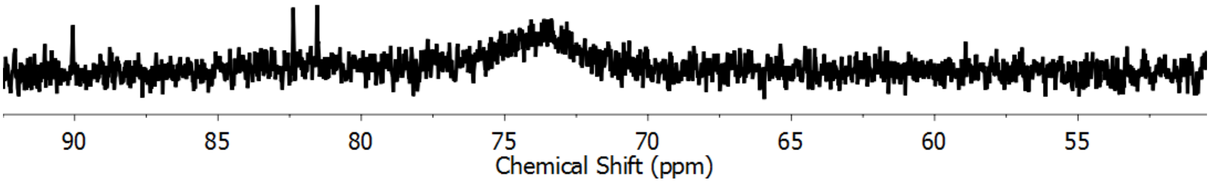

Figure S6: The solution ^{31}P{^{1}H} NMR spectrum (CD_2Cl_2, 298 K, 162 MHz) of **[1-(ethene)$_2$][BAr^F$_4$]** prepared by dissolving a crystalline sample of **[1-(ethene)$_2$][BAr^F$_4$]-*Oct*** which was synthesized by the solid/gas route. The resonance at approximately 82 ppm is due to the presumed solvent induced decomposition product.

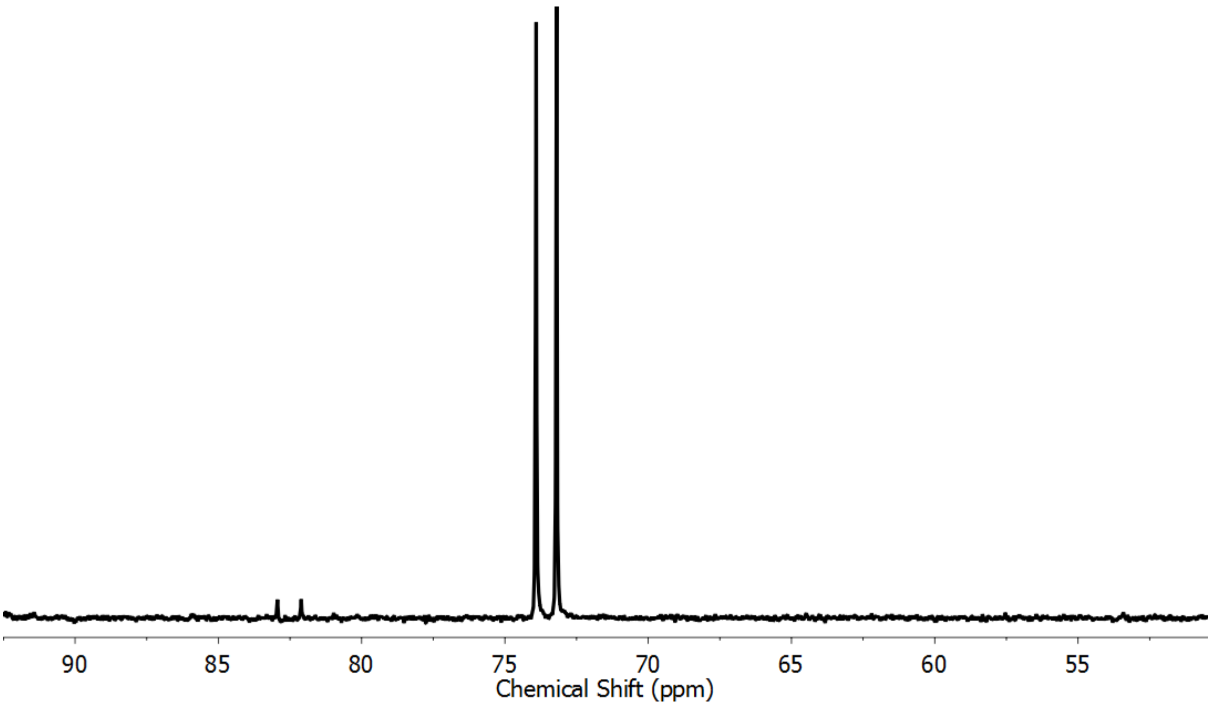

Figure S7: The solution ^{31}P{^{1}H} NMR spectrum (CD_2Cl_2, 193 K, 162 MHz) of **[1-(ethene)$_2$][BAr^F$_4$]** prepared by dissolving a crystalline sample of **[1-(ethene)$_2$][BAr^F$_4$]-*Oct*** which was synthesized by the solid/gas route. The spectrum was meaured on the the same sample as figure S6, and the solvent induced decomposition product is still present at 82 ppm.

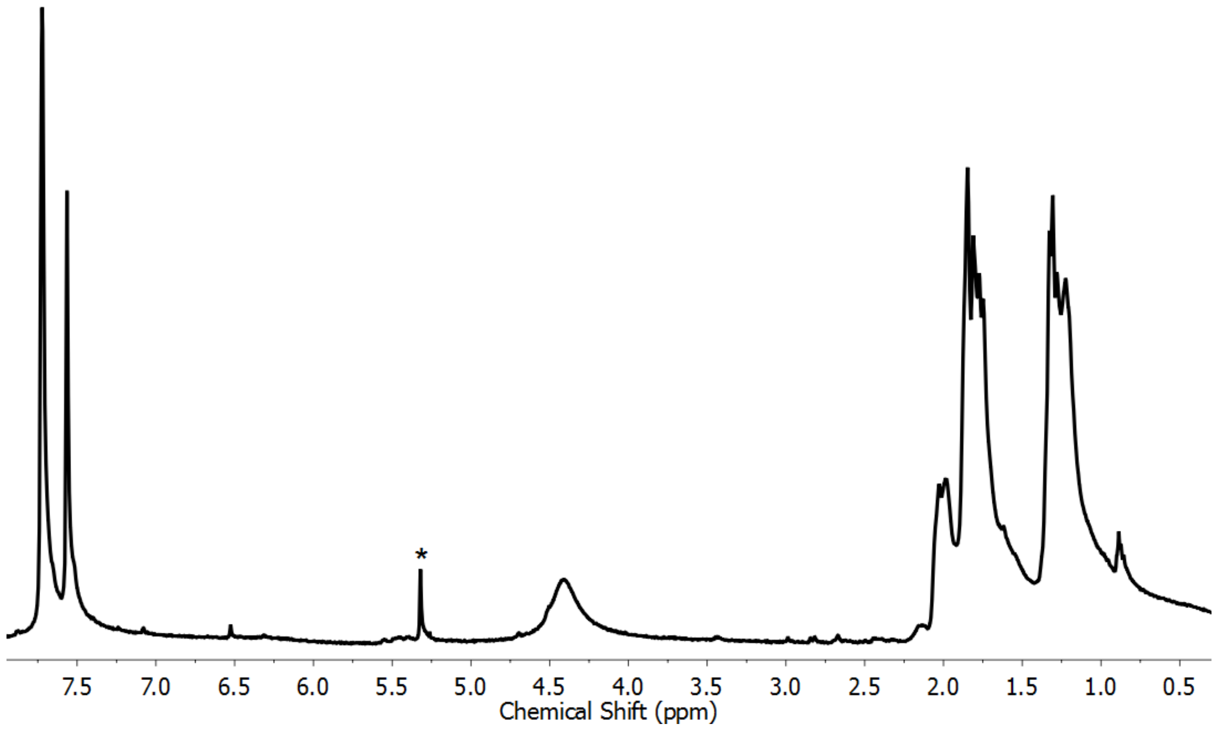

Figure S8: The solution ^{1}H NMR spectrum (CD_2Cl_2, 298 K, 400 MHz) of **[1-(ethene)$_2$][BAr$^F_4$]** prepared by dissolving a crystalline sample of **[1-(ethene)$_2$][BAr$^F_4$]-*Hex***) which was synthesized by the solid/gas route. The resonance marked * is due to residual protio solvent. This spectrum is identical in all the key features to that in figure S4.

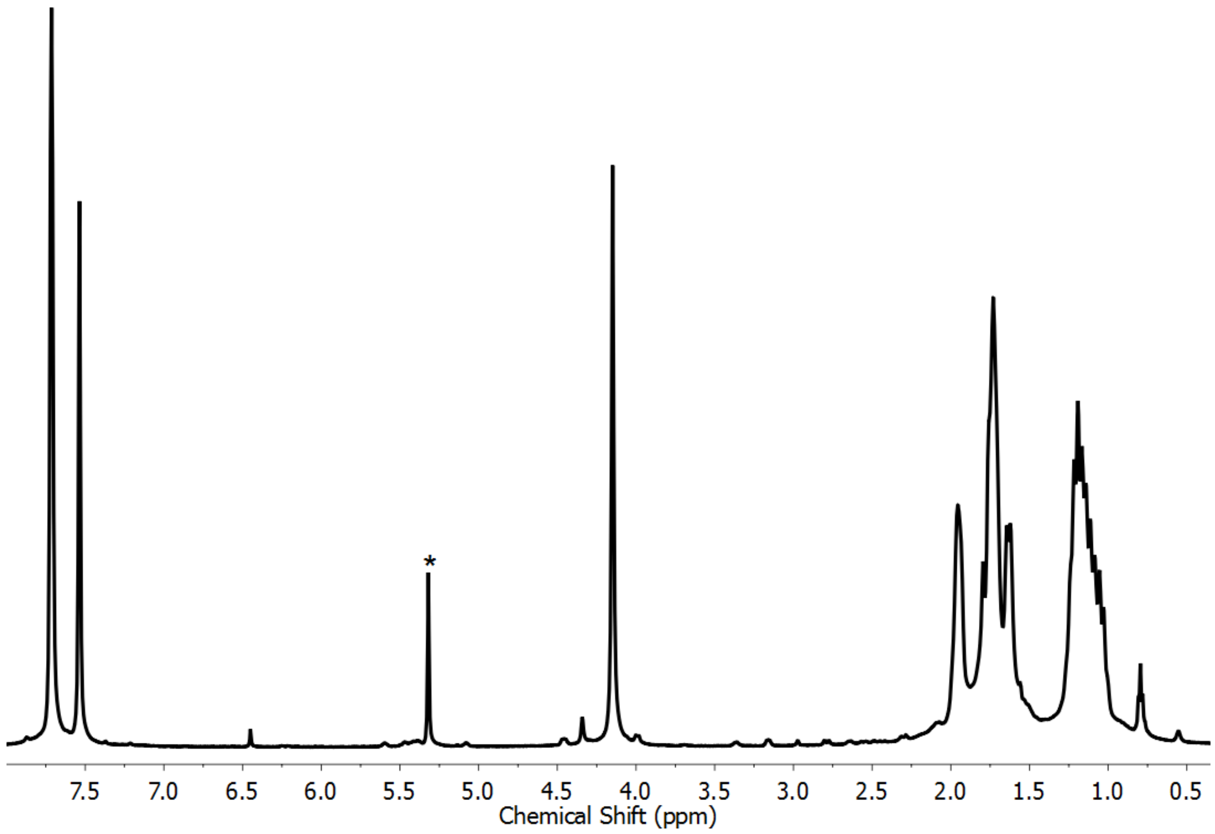

Figure S9: The solution ^{1}H NMR spectrum (CD_2Cl_2, 193 K, 500 MHz) of **[1-(ethene)$_2$][BAr^F$_4$]** prepared by dissolving a crystalline sample of **[1-(ethene)$_2$][BAr^F$_4$]-*Hex*)** which was obtained by the solid/gas route. The resonance marked * is due to residual protio-solvent. This spectrum is identical in all the key features to that in figure S5.

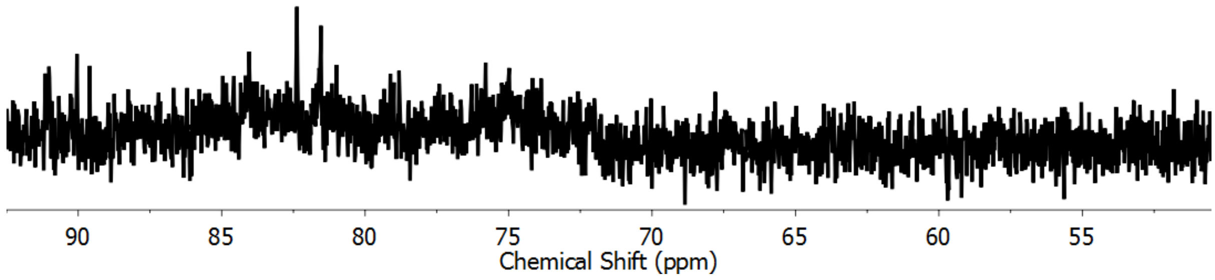

Figure S10: The solution $^{31}P\{^1H\}$ NMR spectrum (CD_2Cl_2, 298 K, 162 MHz) of **[1-(ethene)$_2$][BAr^F$_4$]** (from dissolved **[1-(ethene)$_2$][BAr^F$_4$]-*Hex*)** measured at room temperature. The resonance at approximately 82 ppm is due to the presumed solvent induced decomposition product.

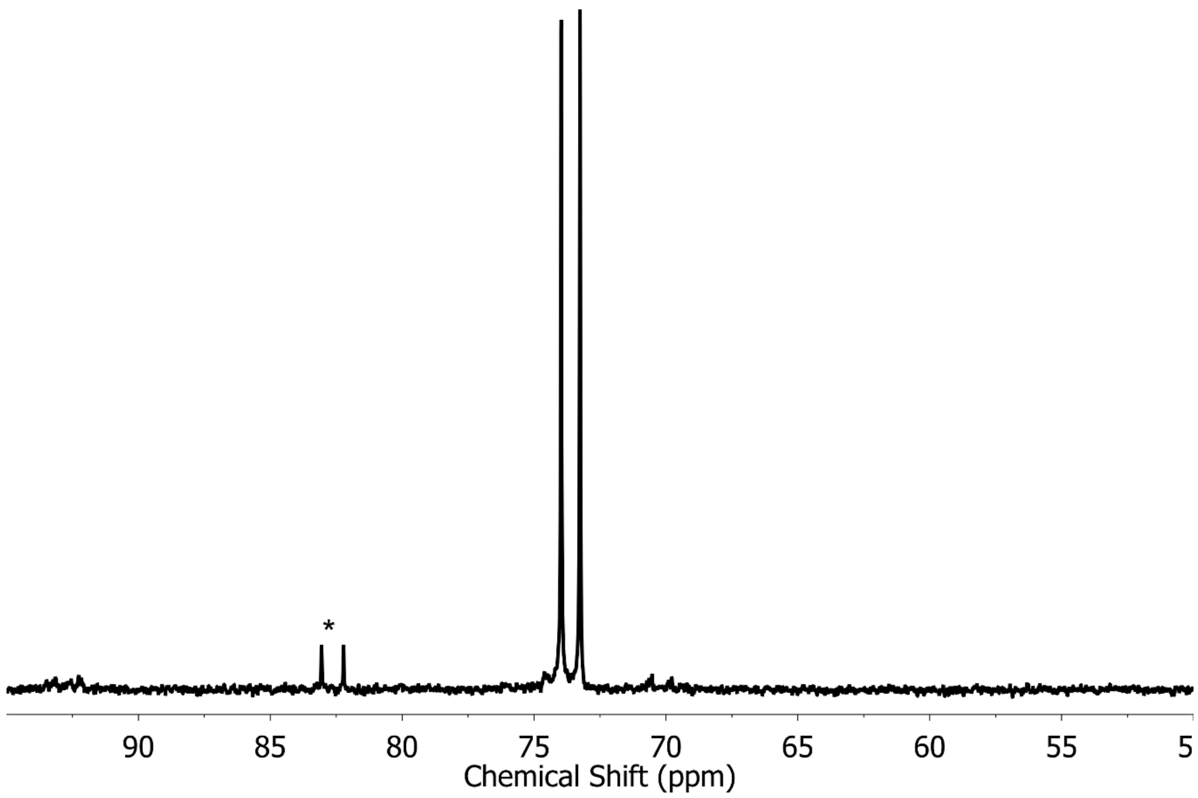

Figure S11: The solution $^{31}P\{^{1}H\}$ NMR spectrum (CD_2Cl_2, 193 K, 162 MHz) of **[1-(ethene)$_2$][BAr$^F_4$]** prepared by dissolving **[1-(ethene)$_2$][BAr$^F_4$]-*Hex*** which was synthesized by the solid/gas route. This spectrum is effectively identicle to that in figure S7. The resonance marked * is due to the decomposition of the sample in the solvent (to unidentified products, *vide supra*).

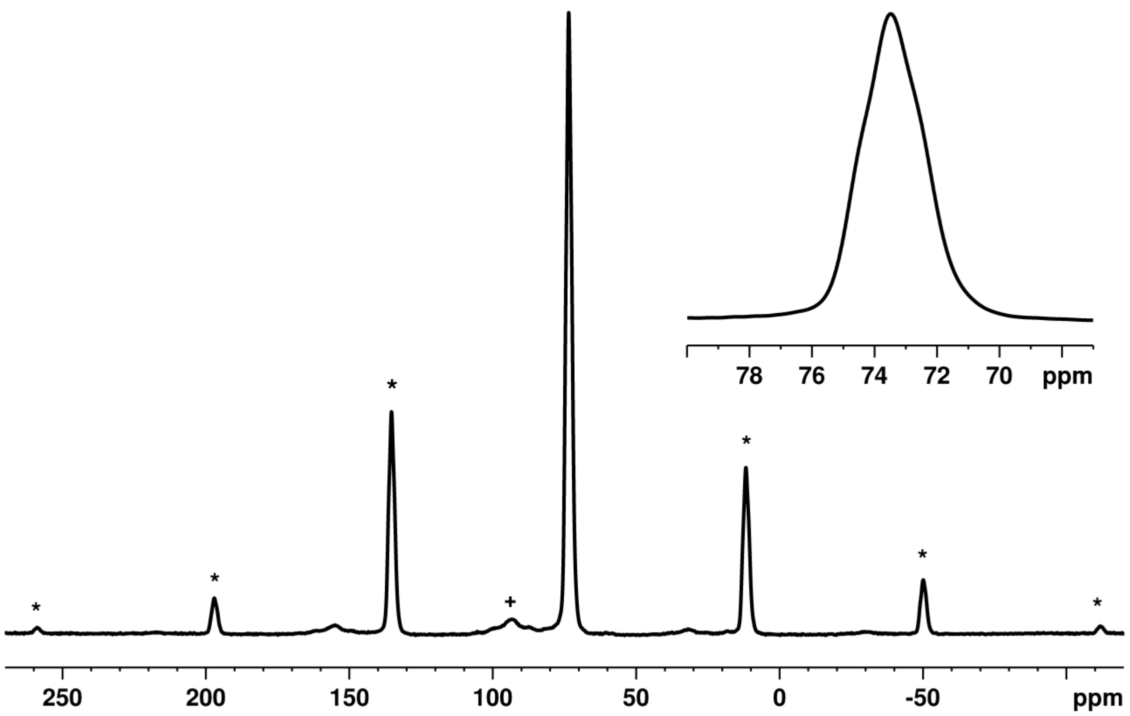

Figure S12: The $^{31}P\{^{1}H\}$ SSNMR spectrum (162 MHz, 298 K, 10 kHz spin rate) of **[1-(ethene)][BAr$^{F}_{4}$]-*Oct*** complex prepared *ex-situ*. The resonance marked + is due to unknown impurities. The resonances marked * are due to spinning sidebands. The inset is a zoom of the central resonances.

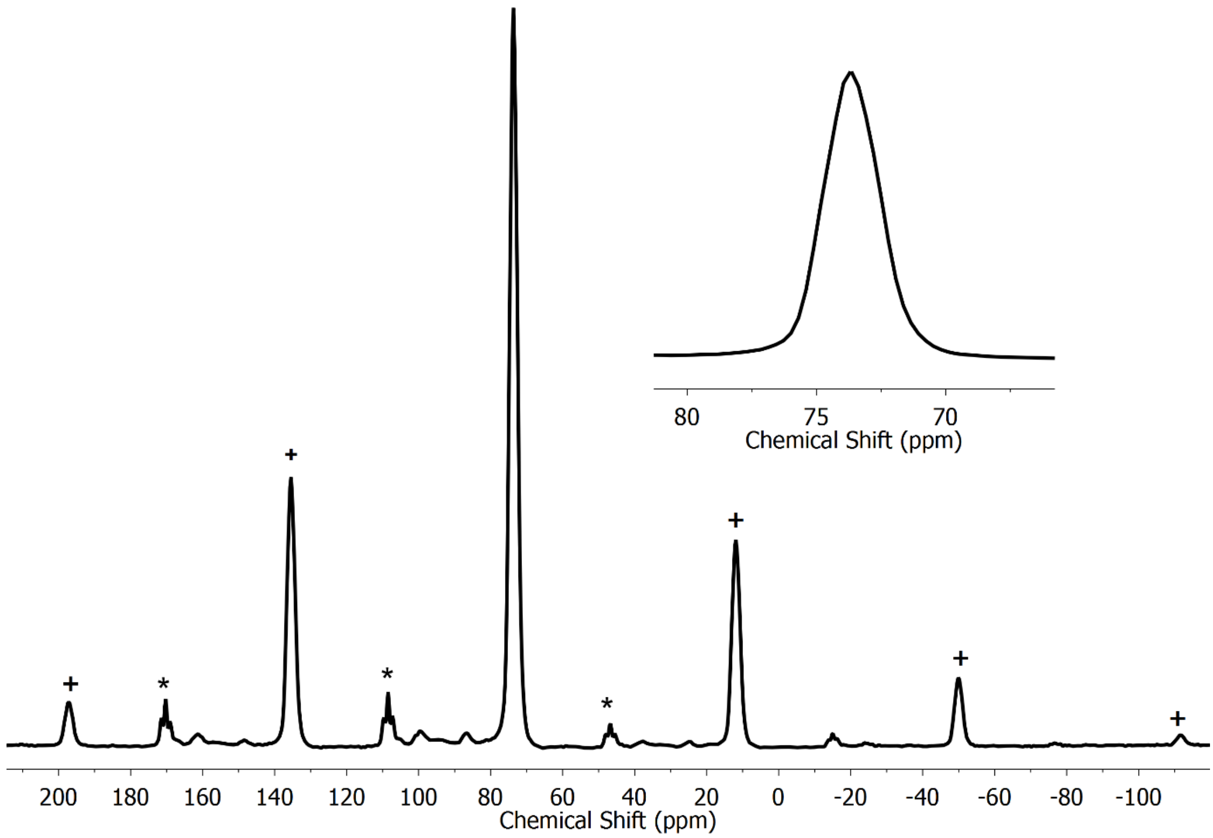

Figure S13: The $^{31}P\{^{1}H\}$ SSNMR spectrum (162 MHz, 298 K, 10 kHz spin rate) of **[1-(ethene)$_2$][BAr$^F{}_4$]-*Oct*** prepared by reacting a crystalline sample **[1-NBA][BAr$^F{}_4$]** with ethene (15 psi) in a SSNMR rotor for 2 hours. The inset is a zoom of the central resonance. Resonances marked + are spinning sidebands, those marked * correspond to residual starting material (and respective spinning sidebands). Due to the experimental set up of SSNMR and the reaction taking place in the rotor reaction rates are considerably slower, and in this case, did not go to completion.

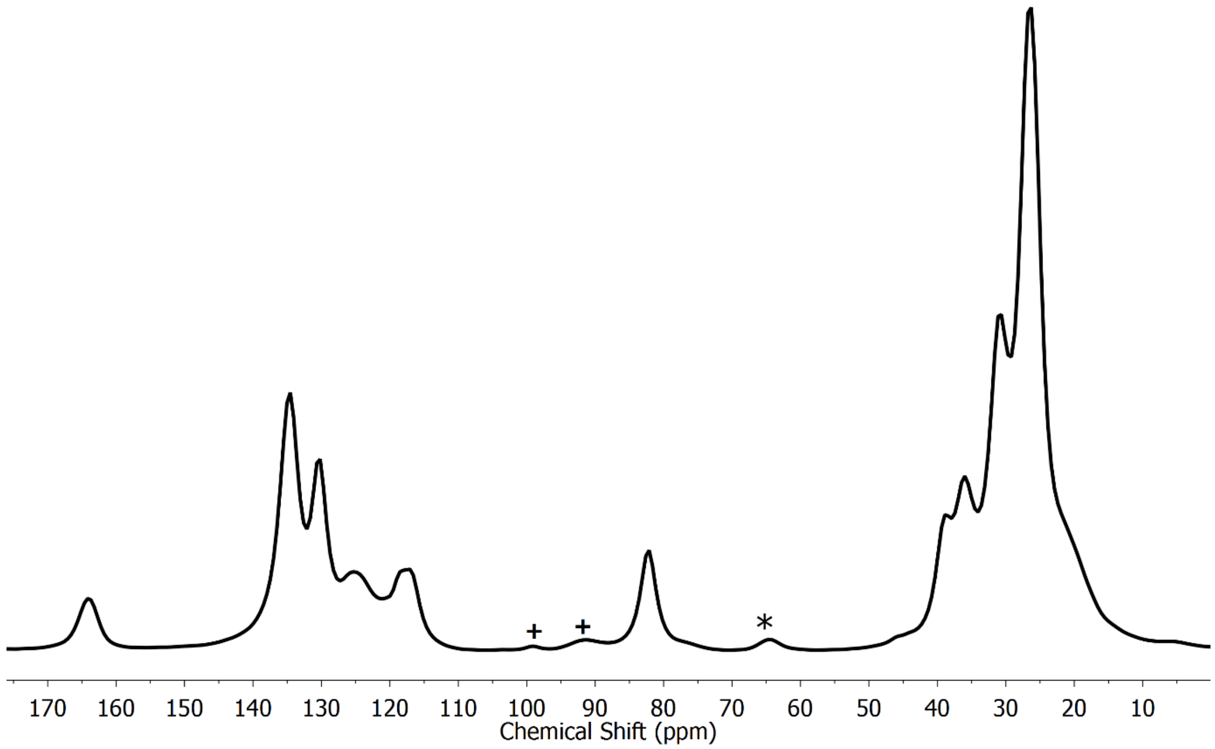

Figure S14: The $^{13}C\{^{1}H\}$ SSNMR spectrum (101 MHz, 298 K, 10 kHz spin rate) of **[1-(ethene)$_2$][BAr^F$_4$]-*Oct*** prepared by reacting a crystalline sample of **[1-NBA][BAr^F$_4$]** with ethene (15 psi) in a SSNMR rotor for 2 hours. The resonance marked * is a spinning sideband, those marked + are due to a small amount of **[1-(butadiene)][BAr^F$_4$]**, which comes from the dehydrogenative coupling of ethene (*vide infra*). Due to the experimental set up of SSNMR and the reaction taking place in the rotor reaction rates are considerably slower.

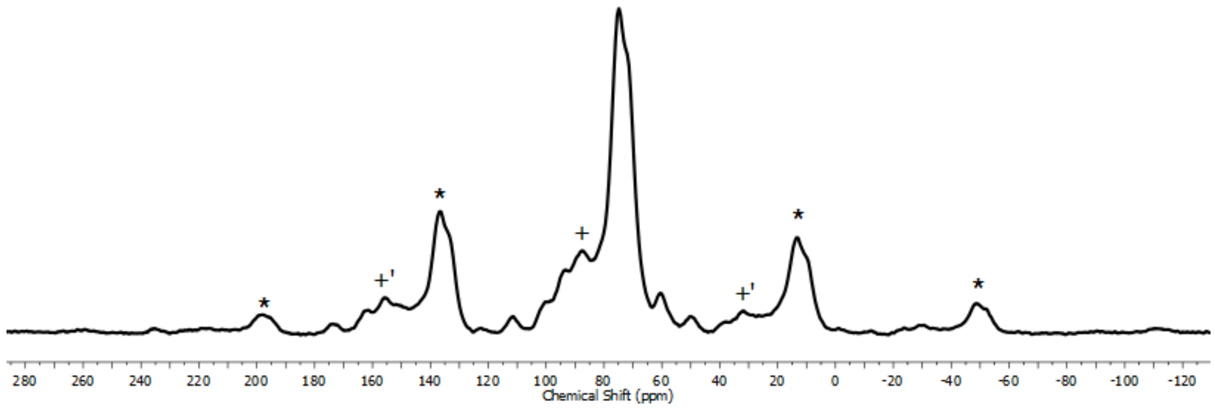

Figure S15: The $^{31}P\{^{1}H\}$ SSNMR spectrum (162 MHz, 298 K, 10 kHz spin rate) of isolated crystalline **[1-(ethene)][BAr^F$_4$]-*Hex***. The resonances marked with + and its associated +' sidebands are presumably due to the formation of **[1-(butadiene)][BAr^F$_4$]** as a result from the dehydrogenative coupling of ethene (*vide*

infra) and other unknown compounds result of unavoidable decomposition of the sample at room temperature during the acquisition time of this experiment. The resonances marked * are due to spinning sidebands. The inset is a zoom of the central resonances.

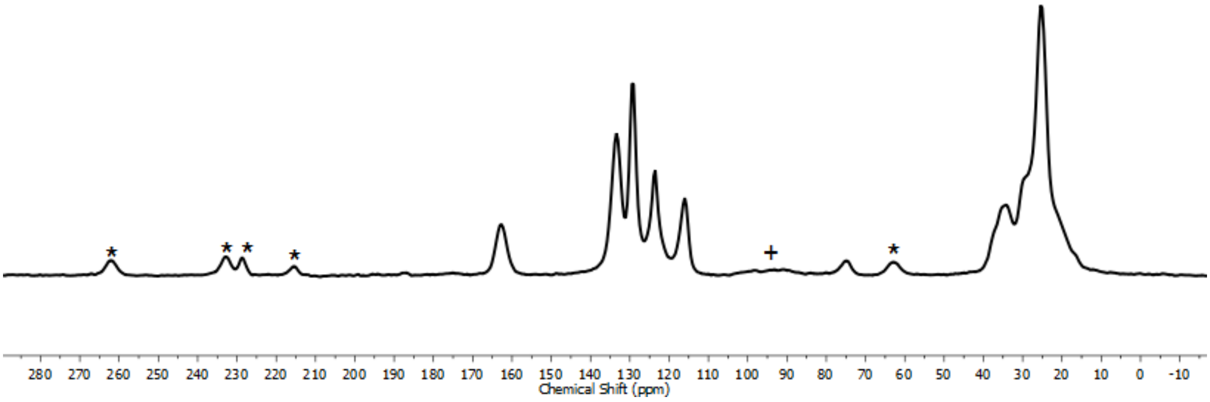

Figure S16: The $^{13}C\{^{1}H\}$ SSNMR spectrum (101 MHz, 298 K, 10 kHz spin rate) of isolated crystalline **[1-(ethene)$_2$][BAr$^F{}_4$]-*Hex***. The resonances marked * are spinning sideband. The broad resonance marked + corresponds to a small amount of **[1-(butadiene)][BAr$^F{}_4$]** formed as a result from the dehydrogenative coupling of ethene (see S.4.4.) at room temperature during the acquisition time of this experiment.

S.2.2. Synthesis and characterization of [1-(propene)][BAr$^F{}_4$] and [1-(propene)$_2$][BAr$^F{}_4$]

S.2.2.1. Attempted solution phase synthesis from [1-F$_2$C$_6$H$_4$][BAr$^F{}_4$]

A crystalline sample of **[1-F$_2$C$_6$H$_4$][BAr$^F{}_4$]** (25 mg, 0.0166 mmol) was taken up in CD_2Cl_2 (0.5 mL) in a high pressure NMR tube. This was freeze-pump-thaw degassed (< 1 × 10^{-2} mbar) three times before propene gas (15 psi) was added. No discernible (by eye) color change occurred. $^{31}P\{^{1}H\}$ NMR spectroscopy indicated that initially significant conversion to a complex assigned as **[1-(propene)$_x$][BAr$^F{}_4$]** had occurred (c. 70%), with the remaining 30% being the starting **[1-F$_2$C$_6$H$_4$][BAr$^F{}_4$]** (see figure S17). However, any attempted work-up involving vacuum results in either starting material being recovered or the complete decomposition of the species, to presumed solvent C-H or C-Cl bond activated products (indicated from mass spectroscopy showing the presence of chloride-bridged rhodium dimers). Furthermore leaving **[1-(propene)][BAr$^F{}_4$]** as prepared by solid/gas routes (see S.2.2.2.) in CH_2Cl_2 solution, at room temperature, resulted in similar decomposition over a period of approximately half an hour.

$^{31}P\{^{1}H\}$ solution NMR (CD_2Cl_2, 298 K, 202 MHz) ***of an in situ reaction mixture* δ: [1-F$_2$C$_6$H$_4$][BAr$^F{}_4$]**, 98.6 (d, J_{RhP} = 201 Hz; *c.f. previously reported*, 98.6, J_{RhP} = 199 Hz);[S6] **[1-(propene)$_x$][BAr$^F{}_4$]**, 91.0 (br d, J_{RhP} = 176 Hz).

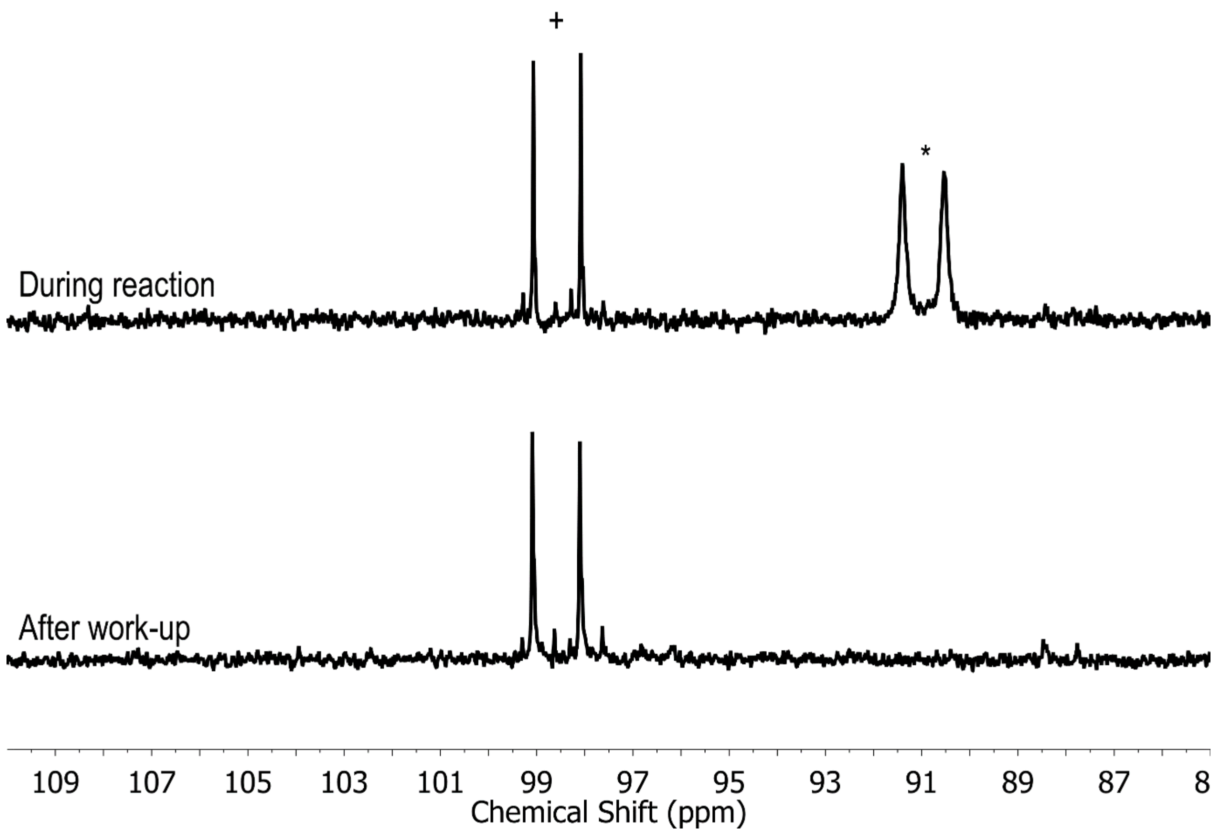

Figure S17: Solution $^{31}P\{^{1}H\}$ NMR (CD_2Cl_2 298 K, 202 MHz) of the attempted solution-phase synthesis of **[1-(propene)$_x$][BAr$^F_4$]** from **[1-$F_2C_6H_4$][BAr$^F_4$]**. The top spectrum is taken during the reaction, the resonance marked + is due to **[1-$F_2C_6H_4$][BAr$^F_4$]** and the resonance marked * is due to a complex assigned to **[1-(propene)$_x$][BAr$^F_4$]**. The bottom spectrum is after the attempted work-up (removing solvents in vacuo, washing with pentane), and there is no resonance attributable to **[1-(propene)$_x$][BAr$^F_4$]**.

S.2.2.2. Solid state synthesis and characterization of [1-(propene)][BAr$^F_4$]

To an orange sample of crystalline **[1-NBA][BAr$^F_4$]** (25 mg, 0.0168 mmol) in an evacuated (< 1 × 10^{-2} mbar) J. Young's flask (c. 50 mL) propene gas (15 psi, 298 K) is added and left standing for 2 hours. Little color change is observed, but evidence of a colorless liquid-oil is sometimes observed on the sides of the flask (assumed to be liberated NBA) – it is this sample that was used for spectroscopic analysis. Yield: Quantitative (> 95%) by $^{31}P\{^{1}H\}$ solution and SSNMR (no other signals observed).

Notes: This compound appears stable for at least 72 hours at room temperature under a propene atmosphere, as shown by $^{31}P\{^{1}H\}$ SSNMR spectroscopy. The longer-term stability under an argon atmosphere has not been investigated. Dissolving the material in 1,2-$F_2C_6H_4$ resulted in the formation of **[1-$F_2C_6H_4$][BAr$^F_4$]**.[S6]

^{1}H solution NMR (CD_2Cl_2, 500 MHz, 298 K) δ: 7.72 (8H, s o-BAr$^F_4$), 7.56 (4H, s, p-BAr$^F_4$), 5.07 (v br, propene), 2.10-1.00 (60H, multiple overlapping aliphatic

resonance). It is noted the overall integral of the aliphatic region is high, this is attributed to recalcitrant NBA in the lattice.

$^{31}P\{^1H\}$ solution NMR (CD_2Cl_2, 202 MHz, 298 K) δ: 95.2 (br d, J_{RhP} = 181 Hz).

^1H solution NMR (CD_2Cl_2, 500 MHz, 193 K) δ: 7.71 (8H, s, o-$BAr^F{}_4$), 7.54 (4H, s, p-$BAr^F{}_4$), 4.84 (1H, br, propene), 4.54 (1H, br, propene), 3.55 (1H, br, propene), 2.02-0.94 (57H, multiple overlapping aliphatic resonances), -0.02 (3H, br, propene agostic CH_3). It is noted the overall integral of the aliphatic region is high, this is attributed to recalcitrant NBA in the lattice.

$^{31}P\{^1H\}$ solution NMR (CD_2Cl_2, 202 MHz, 193 K) δ: 100.4 (br, J_{RhP} = 200 Hz), 89.9 (br, J_{RhP} = 161 Hz).

$^{13}C\{^1H\}$ solution NMR (CD_2Cl_2, 126 MHz, 193 K) δ: 161.4 (1:1:1:1 q, J_{BC} = 50 Hz, ipso-ArC), 134.3 (ortho-ArCH), 128.3 (q, J_{CF} = 32 Hz, meta-ArC), 124.1 (q, J_{CF} = 273 Hz, CF_3), 117.2 (para-ArCH), 95.2 (br s, propene C=C), 77.2 (br s, propene C=C), 36.3 (br m, Cy-CH), 29.8 (br s, Cy-CH_2), 29.2 (s, Cy-CH_2), 28.5 (br s, Cy-CH_2), 26.2 (br s, PCH_2), 25.3 (br s, Cy-CH_2), 23.7 (br s, Cy-CH_2), 19.3 (br s, Cy-CH_2), 11.6 (br s, propene CH_3).

$^{31}P\{^1H\}$ SSNMR (162 MHz, 298 K, 10 kHz spin rate) δ: 95.6 (asym. br. s, $v_{1/2}$ = 503 Hz).

$^{31}P\{^1H\}$ SSNMR (162 MHz, 158 K, 10 kHz spin rate) δ: 101.3 (br, $v_{1/2}$ = 510 Hz), 90.4 (br, $v_{1/2}$ = 463 Hz).

$^{13}C\{^1H\}$ SSNMR (101 MHz, 298 K, 10 kHz spin rate) δ: 164.0 ($BAr^F{}_4$), 134.4 ($BAr^F{}_4$), 130.4 ($BAr^F{}_4$), 124.5 ($BAr^F{}_4$), 118.4 ($BAr^F{}_4$), 116.9 ($BAr^F{}_4$), 93.7 (v. br, $v_{1/2}$ = 582 Hz), 46-15 (multiple overlapping aliphatic resonances).

$^{13}C\{^1H\}$ SSNMR (158 K, 10 kHz spin rate) δ: 163.7 ($BAr^F{}_4$), 133.8 ($BAr^F{}_4$), 130.1 ($BAr^F{}_4$), 124.6 ($BAr^F{}_4$), 118.4 ($BAr^F{}_4$), 116.1 ($BAr^F{}_4$), 94.2 (Propene C=C), 78.8 (Propene C=C), 46-15 (multiple aliphatic resonances), 6.5 (Propene agostic CH_3).

Mass Spec: Not stable under mass spectrometric conditions. Species observed (with appropriate isotopic distributions) at m/z = $[\{(Cy_2PCH_2CH_2PCy_2)Rh\}_2CH_4Cl_2]^{2+}$; $[(Cy_2PCH_2CH_2PCy_2)Rh(C_4H_8)]^+$; $[(Cy_2PCH_2CH_2PCy_2)Rh(C_5H_6)]^+$; $[(Cy_2PCH_2CH_2PCy_2)Rh(C_6H_6)]^+$.

Elemental analysis found (calc.): C 51.33% (51.21%), H 4.70% (4.56%).

S.2.2.3. Spectra of [1-(propene)][BAr^F$_4$]

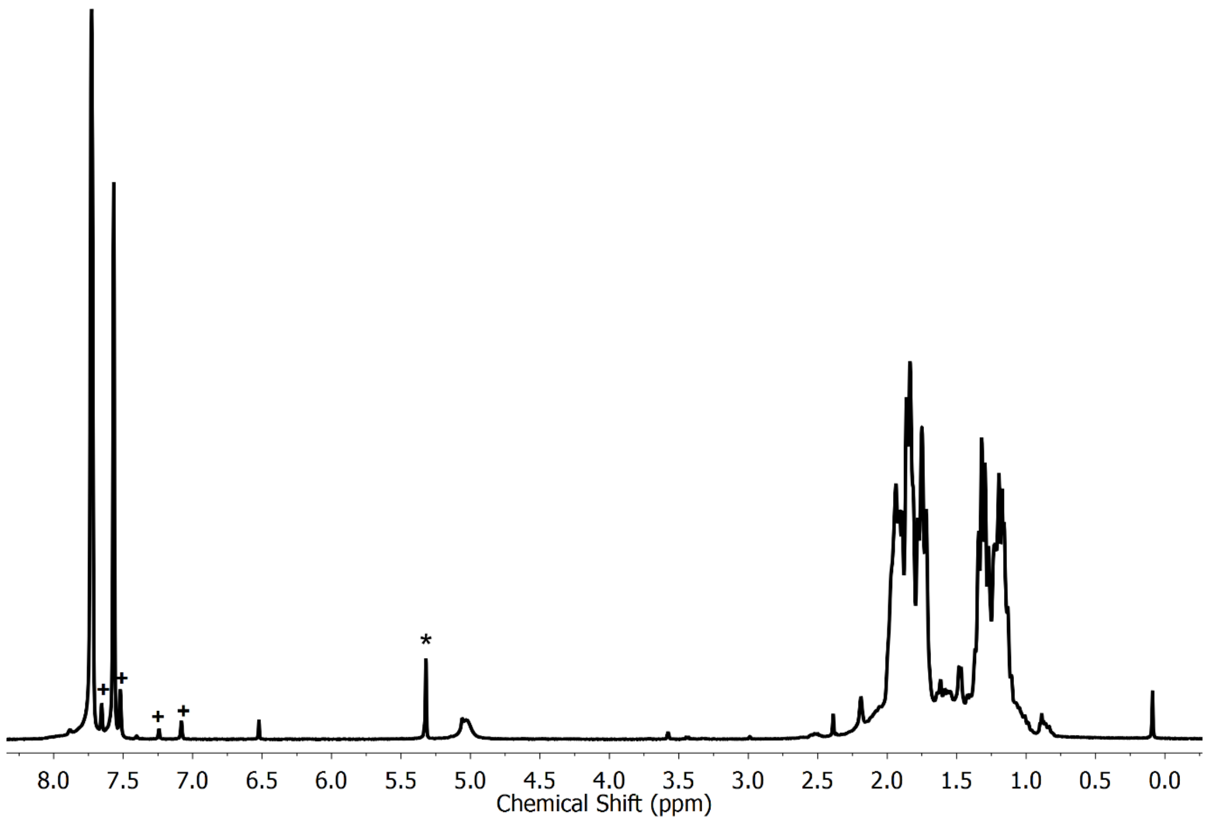

Figure S18: The solution ^{1}H NMR spectrum (CD_2Cl_2, 500 MHz, 298 K) of **[1-(propene)][BAr^F$_4$]**, prepared by the solid/gas route, measured immediately upon dissolution at room temperature. The resonances labelled * and + correspond to residual protio solvent and the previously synthesized zwitterionic BAr^F$_4$ complex **[1-BAr^F$_4$]**,[S1] respectively.

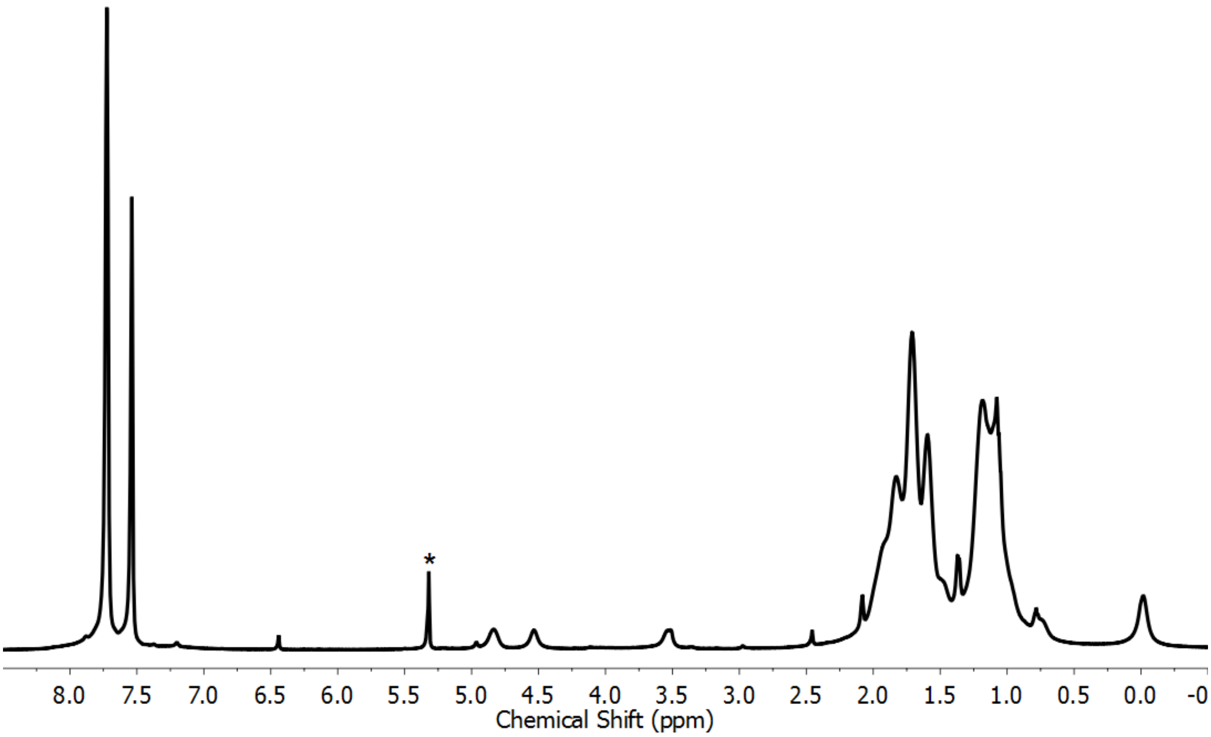

Figure S19: The solution ^{1}H NMR spectrum (CD_2Cl_2, 500 MHz, 193 K) of **[1-(propene)][BAr^F$_4$]**, prepared by the solid/gas route, measured immediately upon dissolution of the sample at 193 K. The resonance marked * is due to residual protio solvent.

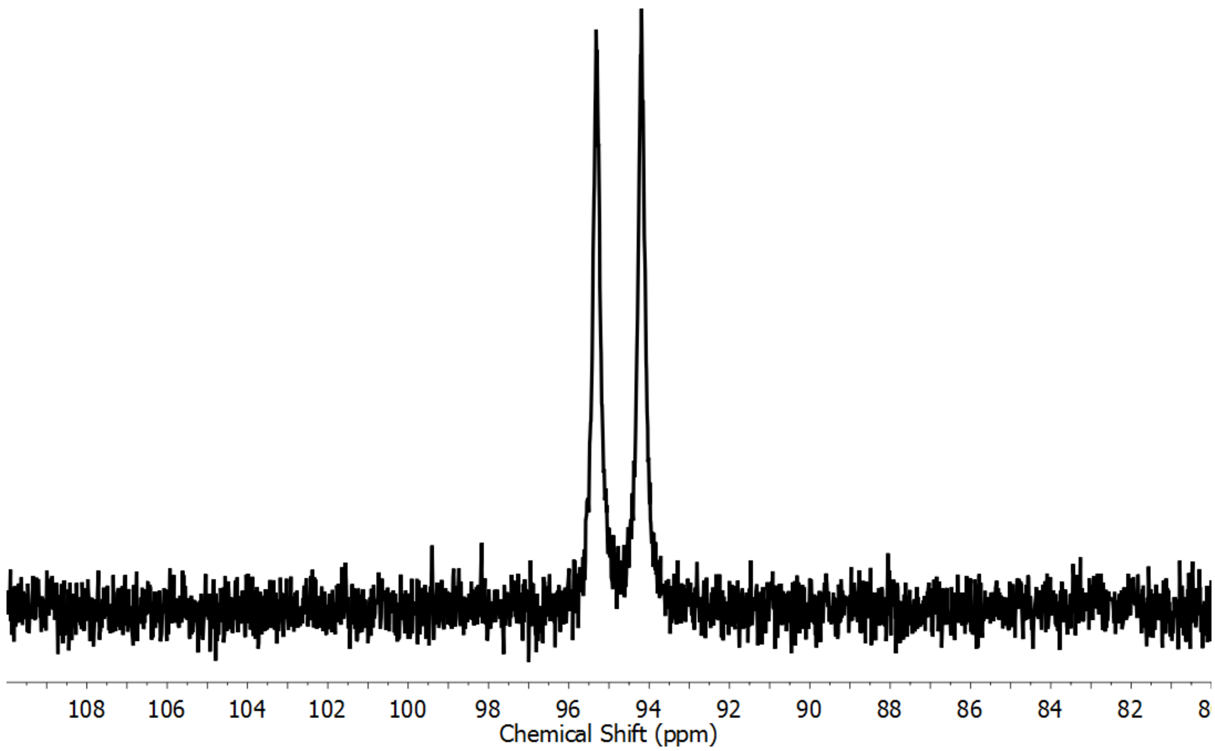

Figure S20: The solution $^{31}P\{^{1}H\}$ NMR spectrum (CD_2Cl_2, 202 MHz, 298 K) of **[1-(propene)][BAr$^F_4$]**, prepared by the solid/gas route, measured immediately upon dissolution at room temperature.

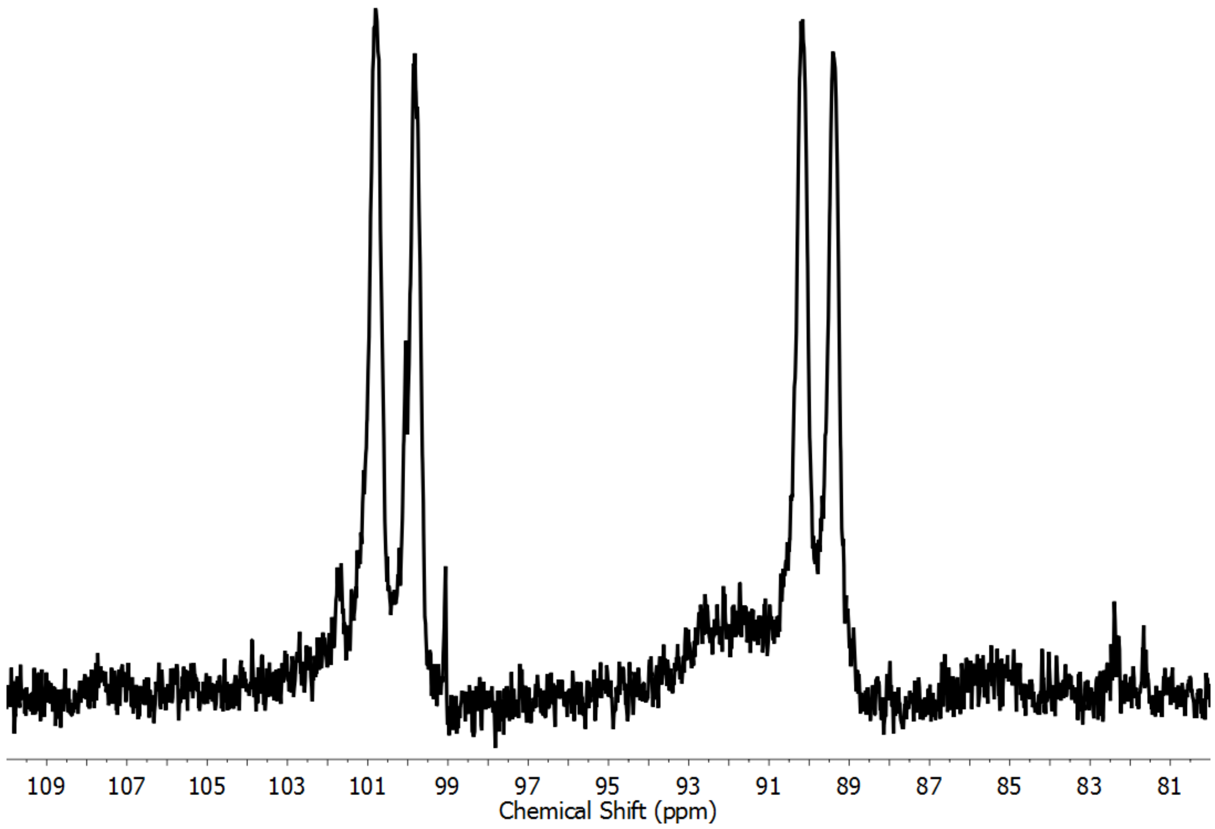

Figure S21: The solution $^{31}P\{^{1}H\}$ NMR spectrum (CD_2Cl_2, 202 MHz, 193 K) of **[1-(propene)][BAr$^F_4$]**, prepared by the solid/gas route, measured at immediately upon dissolution at 193 K.

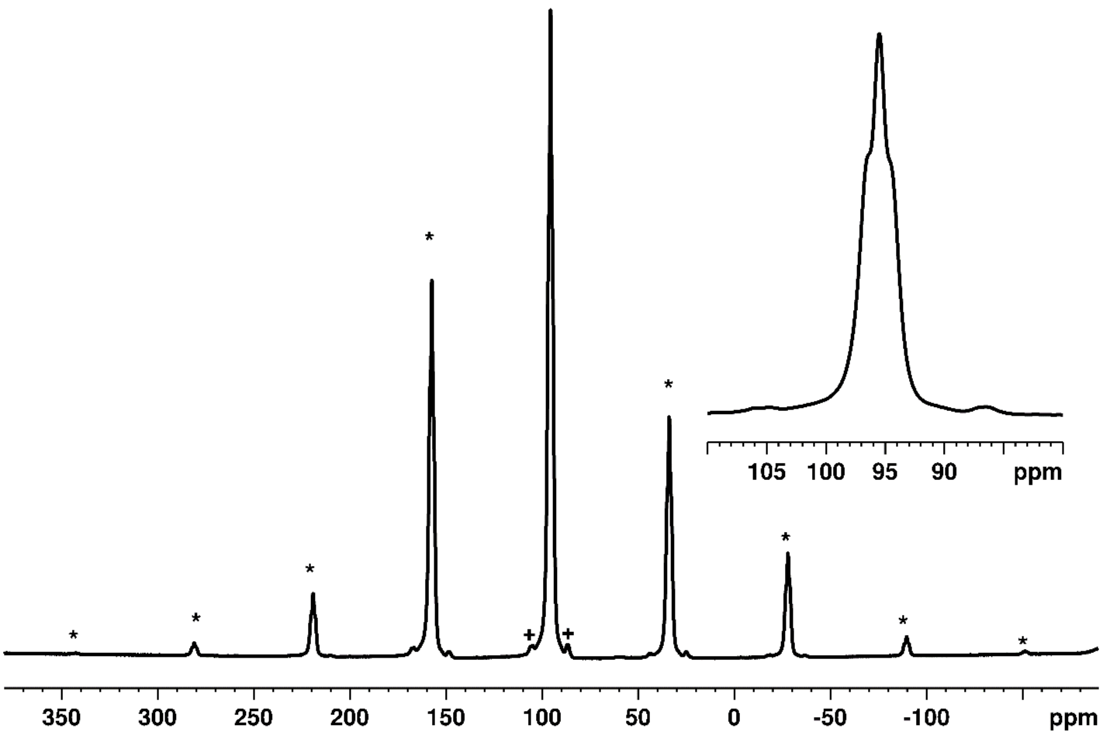

Figure S22: The $^{31}P\{^{1}H\}$ SSNMR spectrum (162 MHz, 298 K, 10 kHz spin rate) of **[1-(propene)][BAr$^{F}_{4}$]** complex prepared *ex-situ* and measured at room temperature. The resonances marked + are attributed to small amounts of unidentified compounds presumably due to unavoidable degradation of the sample during the SSNMR sample preparation and acquisition. The resonances marked * are due to spinning sidebands. The inset is a zoom of the central resonances.

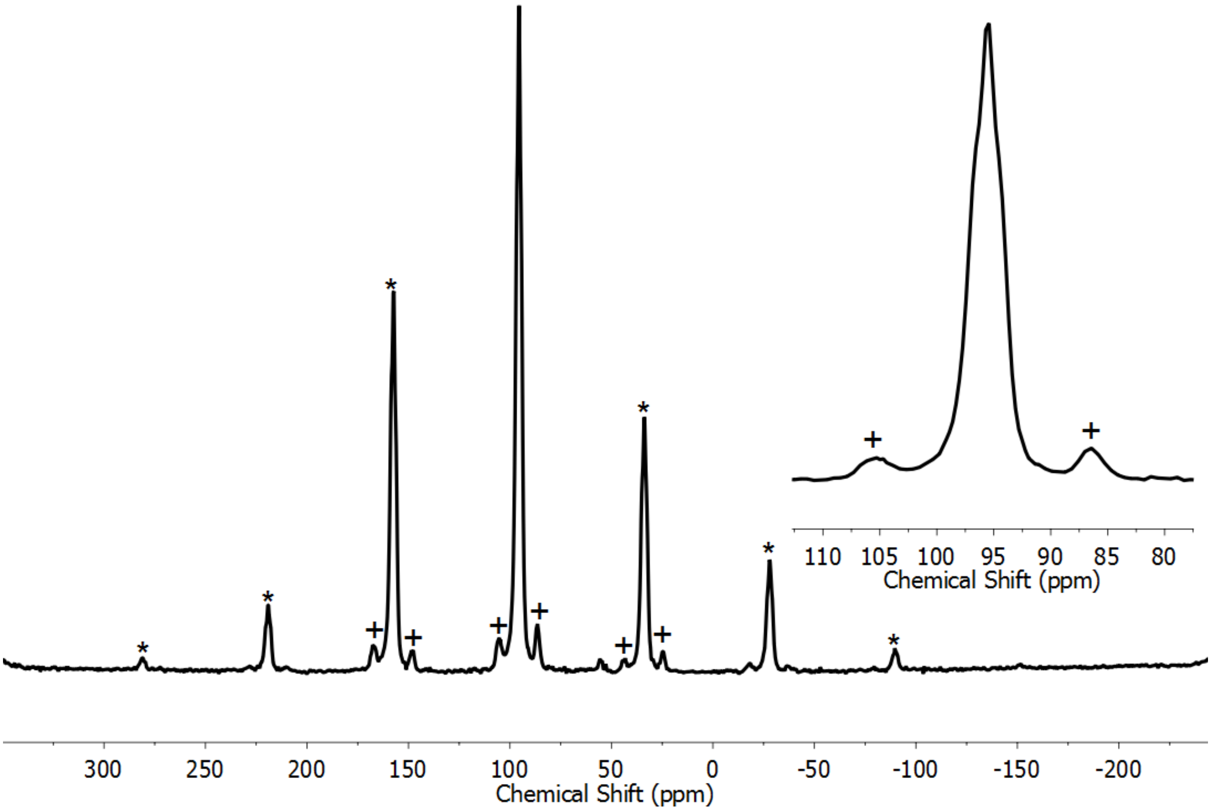

Figure S23: The $^{31}P\{^{1}H\}$ SSNMR spectrum (162 MHz, 298 K, 10 kHz spin rate) of **[1-(propene)][BAr$^F_4$]** complex, measured at room temperature and prepared *in-situ* by direct addition of propene to a pre-loaded crystalline sample of **[1-NBA][BAr$^F_4$]** into a SSNMR rotor. The resonances marked + are attributed to small amounts of unidentified compounds presumably results from unavoidable degradation of the sample during the SSNMR acquisition process. The resonances marked * are due to spinning sidebands. The inset is a zoom of the central resonances.

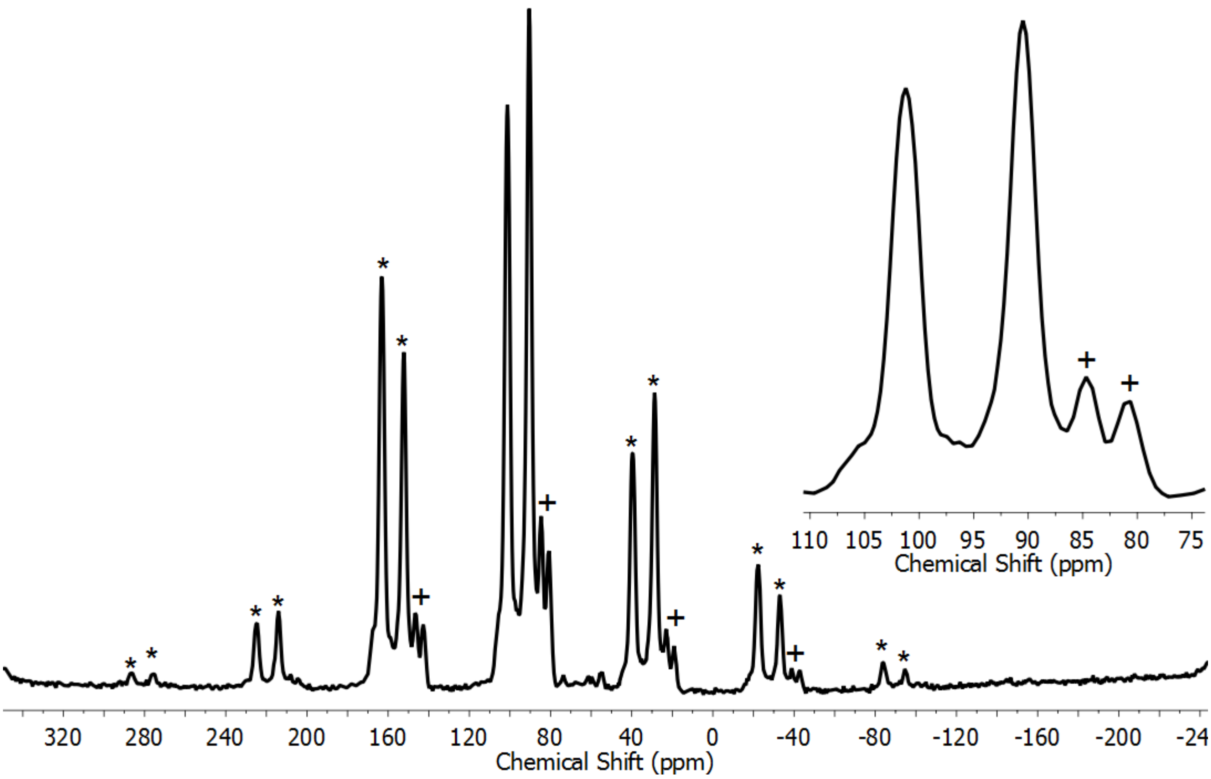

Figure S24: The $^{31}P\{^{1}H\}$ SSNMR spectrum (162 MHz, 158 K, 10 kHz spin rate) of **[1-(propene)][BAr$^{F}{}_{4}$]** *in-situ* prepared by direct addition of propene to a pre-loaded sample of **[1-NBA][BAr$^{F}{}_{4}$]** into a SSNMR rotor. The resonances marked + are attributed to small amounts of unidentified compounds presumably results from unavoidable degradation of the sample during the SSNMR acquisition process. The resonances marked * are due to spinning sidebands. The inset is a zoom of the central resonances.

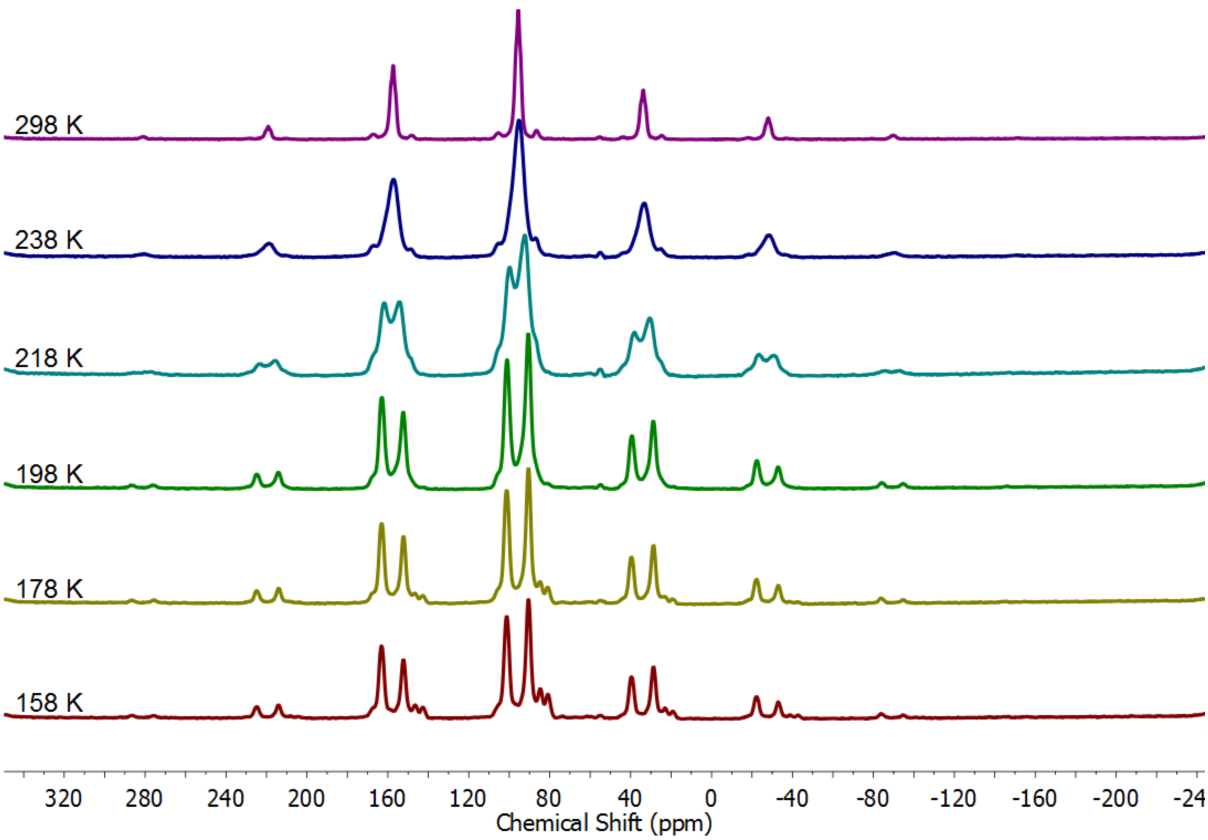

Figure S25: A stack plot of the variable temperature $^{31}P\{^{1}H\}$ SSNMR spectra (162 MHz, 158 K, 10 kHz spin rate) of **[1-(propene)][BAr$^{F}{}_{4}$]**, demonstrating the coalescence of central resonance. The sample was *in-situ* prepared by direct addition of propene to a pre-loaded crystalline sample of **[1-NBA][BAr$^{F}{}_{4}$]** into a SSNMR rotor.

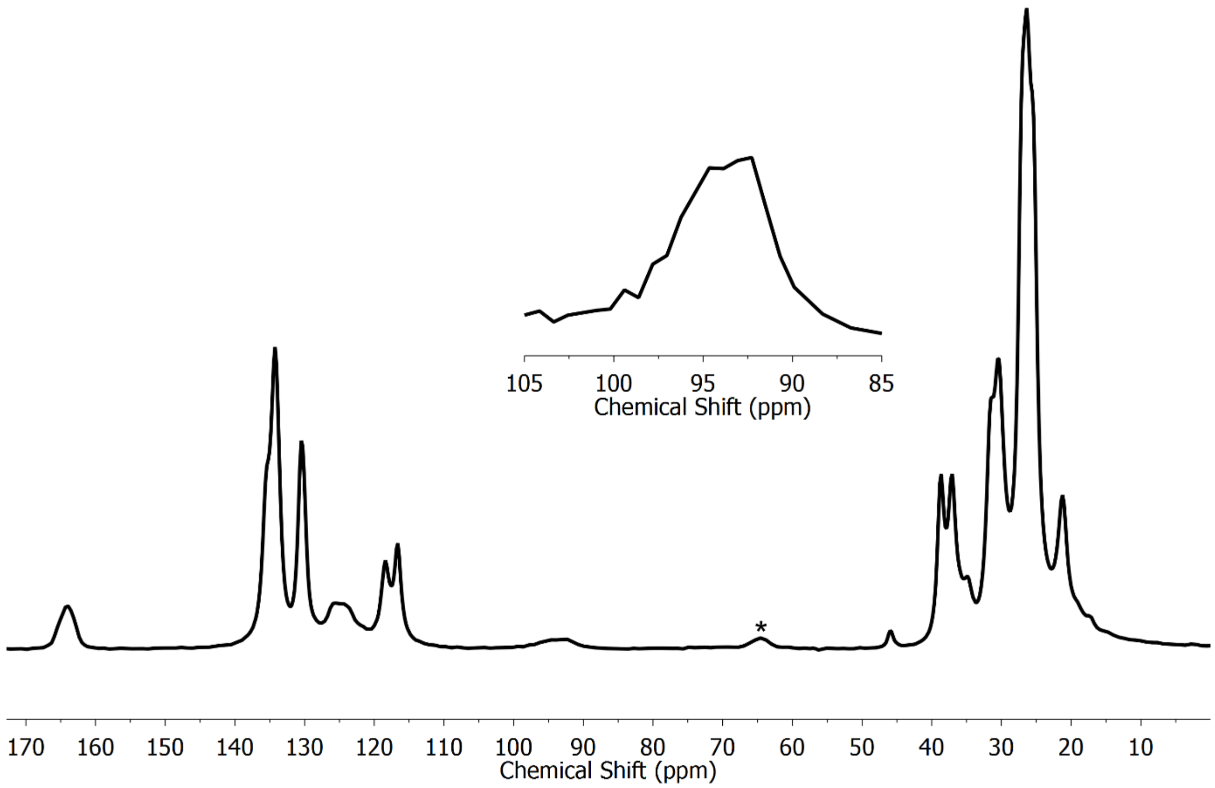

Figure S26: The $^{13}C\{^{1}H\}$ SSNMR spectrum (101 MHz, 298 K, 10 kHz spin rate) of **[1-(propene)][BAr$^F_4$]**, *in-situ* prepared by direct addition of propene to a pre-loaded sample of **[1-NBA][BAr$^F_4$]** into a SSNMR rotor. The resonance marked * is due to a spinning sideband. The inset is a zoom if the broad resonances between 90-100 ppm.

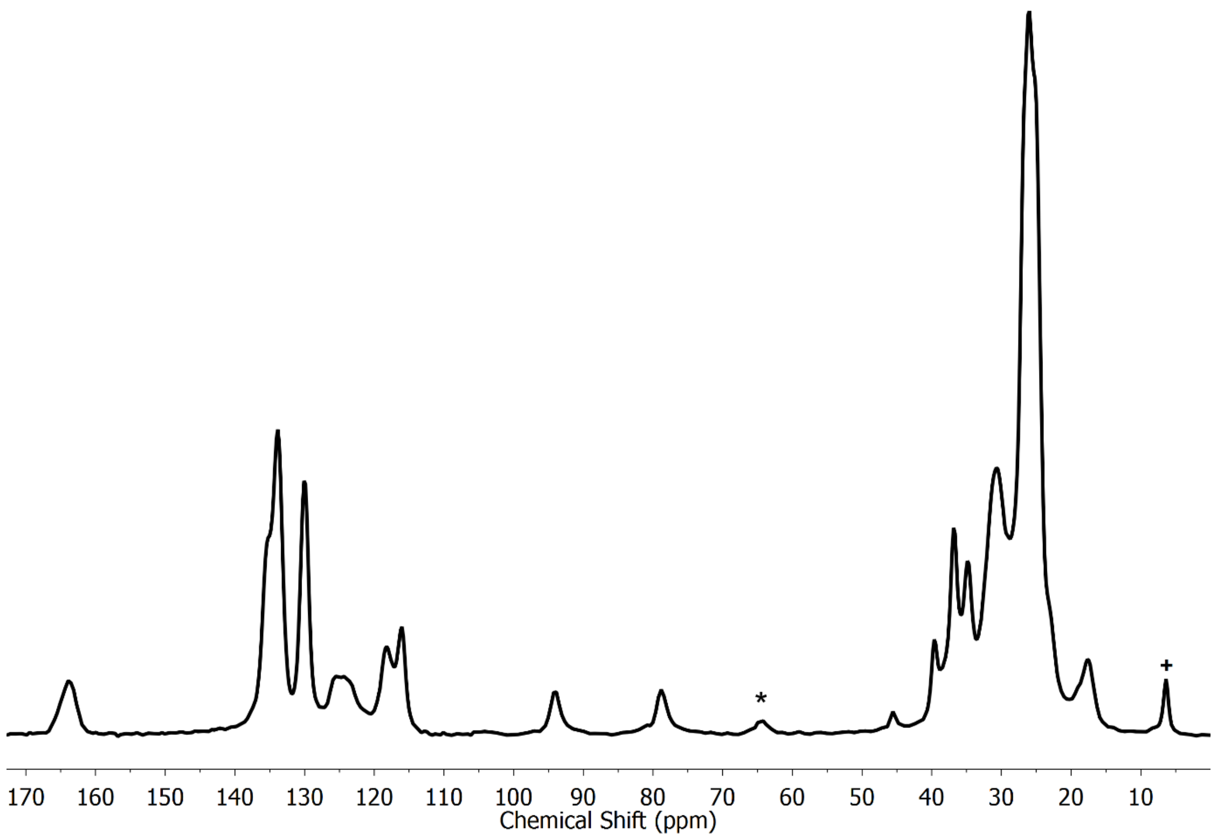

Figure S27: The $^{13}C\{^1H\}$ SSNMR spectrum (101 MHz, 158 K, 10 kHz spin rate) of **[1-(propene)][BAr$^F{}_4$]**, *in-situ* prepared by direct addition of propene to a pre-loaded sample of **[1-NBA][BAr$^F{}_4$]** into a SSNMR rotor. The resonance marked * is due to a spinning sideband. The resonance marked + (~ 6 ppm) is the carbon involved in the C-H agostic interaction.

S.2.2.4. Solution synthesis and characterization of [1-(propene)$_2$][BAr$^F{}_4$]

A crystalline of **[1-NBD][BAr$^F{}_4$]** (120 mg, 0.081 mmol) in an evacuated (< 1 × 10^{-2} mbar) J. Young's flask is reacted with H_2 (15 psi, 298 K) for 6 h to give **[1-NBA][BAr$^F{}_4$]**. After this time, H_2 is removed under vacuum (< 1 × 10^{-2} mbar), then propene (15 psi, 298 K) is added and the sample was left standing for 2 hours to give an orange crystalline sample of **[1-(propene)][BAr$^F{}_4$]** (see S.2.2.2). The crystalline material is then taken up in CH_2Cl_2 (1.5 mL) in a crystallization flask (10 mL) at -78°C under an atmosphere of propene (15 psi, 298 K). Working under an atmosphere of propene (15 psi, 298 K), the solution is quickly filtered *via* cannula and layered with pentane (10 mL) at the same temperature. Storing at -20°C under an atmosphere of propene for a week yields a small crop of orange block like crystals of **[1-(propene)$_2$][BAr$^F{}_4$]** which only allowed characterization by single crystal X-ray diffraction crystallography. Single crystals, directly selected from the mother liquor at -20°C, were suitable for an X-ray diffraction study.

Attempts to recrystallize and isolate **[1-(propene)$_2$][BAr$^F{}_4$]** from **[1-(propene)][BAr$^F{}_4$]**, obtained from the solid-state route, by dissolving the crystalline

material in CH_2Cl_2 at room temperature led to (presumably solvent induced) decomposition over the period of 30 mins.

Notes: CH_2Cl_2 and pentane used in this preparation were freeze-pump-thawed degassed three times and then saturated and stored with propene gas (15 psi, 298 K) prior to use.

S.2.2.5. H/D scrambling studies for [1-(d_3-propene)][$BAr^F{}_4$]

In an effort to elucidate the precise mechanism of isomerization of 1-butene, model experiments were carried out using 3,3,3-d_3-propene. These are discussed more in the main text.

A crushed sample of **[1-NBA][$BAr^F{}_4$]** (20 mg, 0.0135 mmol) was loaded into a high pressure NMR tube in an argon-filled glovebox. This was then sealed using a Teflon stop-cock, before transferring to a Schlenk line and evacuated. The tube was refilled with 3,3,3-d_3-propene (15 psi, 0.075 mmol, 298 K). The head space was then monitored using gas-phase $^2H\{^1H\}$ NMR.

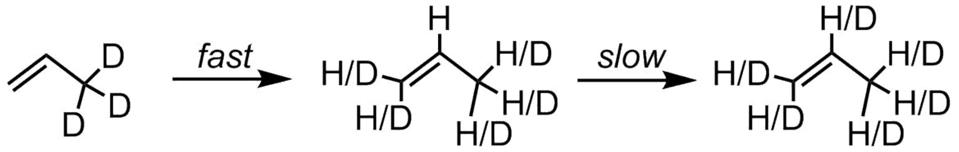

Scheme S1: A summary of the gas phase H/D scrambling of 3,3,3-d_3-propene as catalyzed by **[1-(propene)][$BAr^F{}_4$]**.

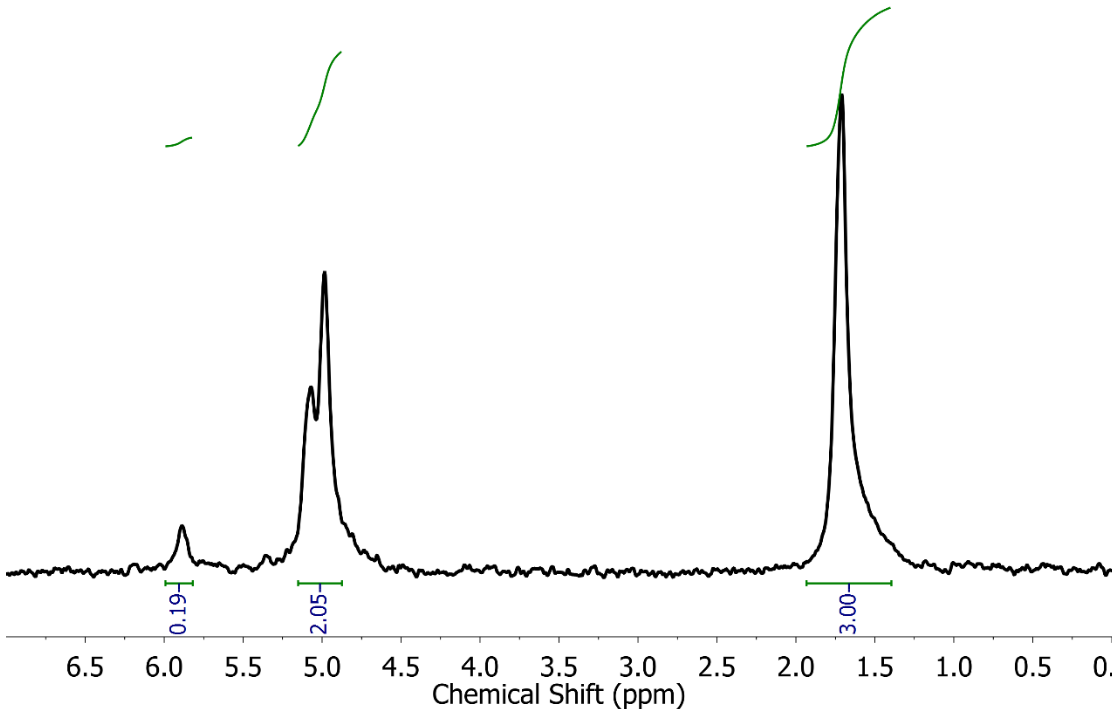

Figure S28: The gas phase $^{2}H\{^{1}H\}$ NMR spectrum (61 MHz, 298 K) of the headspace of the reaction of **[1-NBA][BAr$^{F}_{4}$]** with 3,3,3-d_3-propene after 1 hour. The integrals show that scrambling between the terminal positions (CD_3, δ 1.7 and CD_2, δ 5.1 and 5.0) has effectively gone to completion, whereas the central, C2, position (CD, δ 5.9) is still primarily hydrogen. This spectrum is internally referenced to the CD_3 deuteriums, set to 1.7 ppm.[S7] In propene the *cis*–alkene resonance is observed at δ 4.9 while the *trans* is observed at δ 5.0. The *cis*:*trans* ratio was reliably measured from the associated gas phase ^{1}H NMR spectrum (Figure S29).

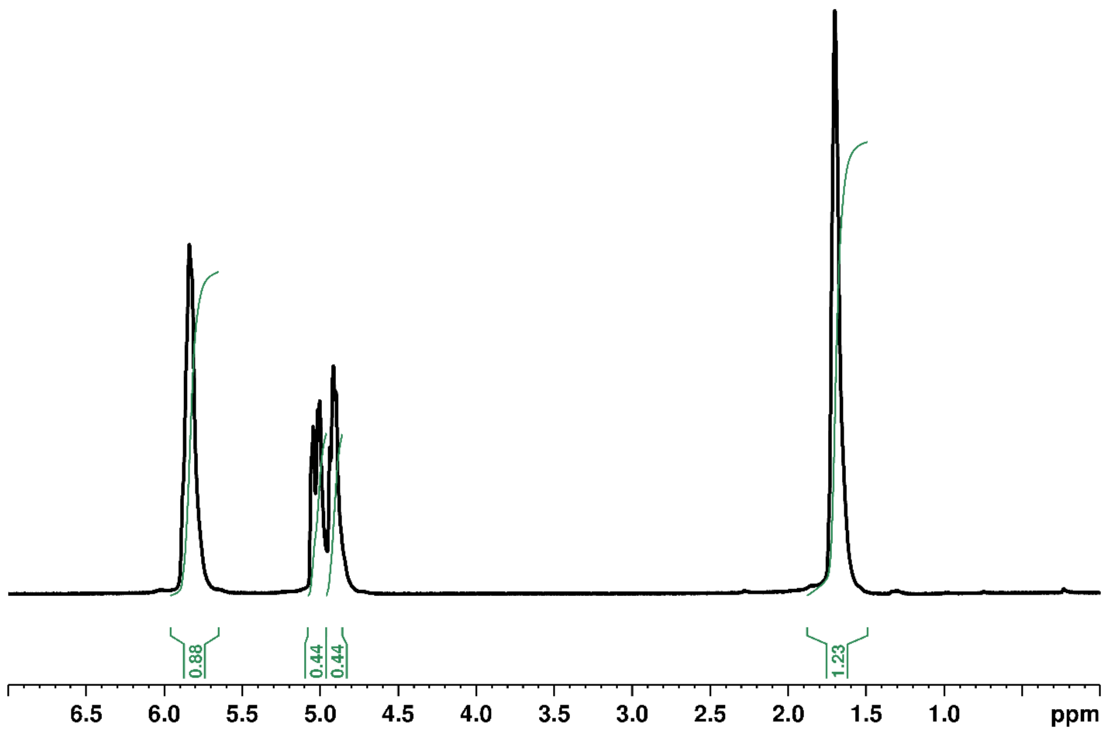

Figure S29: The gas phase ^{1}H NMR spectrum (400 MHz, 298 K) of the headspace of the reaction of **[1-NBA][BAr$^{F}_{4}$]** with 3,3,3-d$_3$-propene after 1 hour. This spectrum is internally referenced to the CH_3 protons, set to 1.7 ppm. Sum of integrals normalized to 3H.

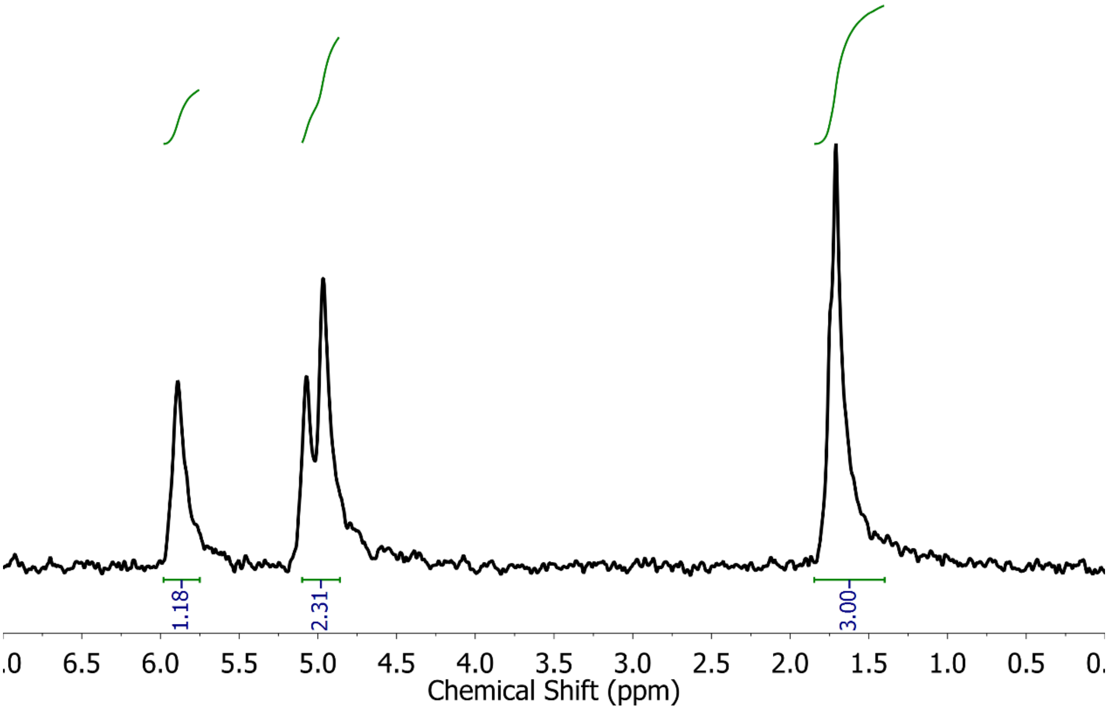

Figure S30: The gas phase $^{2}H\{^{1}H\}$ NMR spectrum (61 MHz, 298 K) of the headspace of the reaction of **[1-NBA][BAr$^{F}_{4}$]** with 3,3,3-d$_3$-propene after 16 hours.

The integrals show that scrambling between all positions has effectively gone to completion (CD_3, δ 1.7; CD_2, δ 5.1 and 4.0; CD δ 5.9). This spectrum is internally referenced to the CD_3 deuteriums, set to 1.7 ppm.[S7] In propene the *cis*–alkene resonance is observed at δ 4.9 while the *trans* is observed at δ 5.0.

S.2.3. Synthesis and characterization of [1-(butene)][BAr$^F_4$]

S.2.3.1. Attempted solution phase synthesis from [1-$F_2C_6H_4$][BAr$^F_4$]

A sample of **[1-$F_2C_6H_4$][BAr$^F_4$]** (20 mg, 0.0133 mmol) in a J. Young's tube (c. 50 mL) was taken up in CH_2Cl_2 before being freeze-pump-thawed degassed three times and 1-butene (15 psi, 298 K) was added. The yellow solution immediately turned orange, and continued to go deeper in color. It was shown (via $^{31}P\{^1H\}$ solution NMR spectroscopy), conversion to **[1-(butadiene)][BAr$^F_4$]** (see S.2.4.2.) would occur over the period of one hour in solution.

S.2.3.2. Solid-state synthesis and characterization of [1-(butene)][BAr$^F_4$]

To an orange crystalline sample of **[1-NBA][BAr$^F_4$]** (25 mg, 0.0168 mmol) in an evacuated (< 1 × 10^{-2} mbar) J. Young's flask (c. 50 mL), 1-butene gas (15 psi, 298 K) was added and left to stand for 2 hours at room temperature. Little color change was observed; however, the crystals stick to the walls of the flask and a colorless oil is sometimes observed on the sides of the flask (assumed to be liberated NBA). The flask was then exposed to dynamic vacuum for 10 minutes (< 1 × 10^{-2} mbar), the crystalline material was taken into an argon glove box. Quantitative consumption of **[1-NBA][BAr$^F_4$]** is observed to give **[1-(butene)][BAr$^F_4$]** (typically contaminated with approximately 10% of **[1-(butadiene)][BAr$^F_4$]** (see S.2.4.), as evidenced by $^{31}P\{^1H\}$ SSNMR spectroscopy).

<u>Notes:</u> To-date we have been unable to prepare **[1-(butene)][BAr$^F_4$]** free of **[1-(butadiene)][BAr$^F_4$]** contamination. Samples typically consist of **[1-(butene)][BAr$^F_4$]** (~90%) and **[1-(butadiene)][BAr$^F_4$]** (~10%) as evidence by $^{31}P\{^1H\}$ SSNMR spectroscopy (see Figure S33). In the presence of excess 1-butene (15 psi, 298 K), powdered **[1-(butene)][BAr$^F_4$]** forms **[1-(butadiene)][BAr$^F_4$]** (~90%, 48 h, 298 K) as evidence by $^{31}P\{^1H\}$ solution and SSNMR spectroscopy . In the absence of 1-butene, **[1-(butene)][BAr$^F_4$]** decomposes slowly in the solid state at room temperature to afford **[1-(butadiene)][BAr$^F_4$]** (~50%) and other unidentified decomposition products (~50%). When dissolved in CD_2Cl_2 at room temperature, **[1-(butene)][BAr$^F_4$]** rapidly decomposes to **[1-(butadiene)][BAr$^F_4$]** (~50%) and **[1-BAr$^F_4$]**[S1] (50%) observed by $^{31}P\{^1H\}$ NMR spectroscopy, the latter decomposing slowly at room temperature in CD_2Cl_2 to afford unidentified products. Decomposition in CD_2Cl_2 is effectively halted at low temperatures. Samples of **[1-(butene)][BAr$^F_4$]** used for solution state NMR characterization were prepared as follows. To a high pressure NMR tube containing a crystalline sample of **[1-(butene)][BAr$^F_4$]** (15 mg), CD_2Cl_2 (0.4 mL) was added by condensation. The CD_2Cl_2 was rapidly thawed and the NMR tube was then briefly

shaken (20 s) whilst still cold to dissolve the crystals, affording an orange solution. The NMR tube was quickly transferred to a precooled NMR spectrometer and spectra were immediately acquired. Carefully prepared solution NMR samples of **[1-(butene)][BAr$^F_4$]** are typically contaminated with **[1-(butadiene)][BAr$^F_4$]** (~25%) based on ^{1}H and ^{31}P{^{1}H} NMR spectroscopy.

^{1}H solution NMR (CD_2Cl_2, 500 MHz, 193 K) δ: 7.73 (11H, s, o-BAr$^F_4$), 7.55 (5.5H, s, p-BAr$^F_4$), 5.08 (2H, s, br, butene C=CH), 2.10-0.90 (68H, multiple overlapping aliphatic resonances), 0.56 (6H, s, br, butene CH_3). It is noted the integrals of the BAr$^F_4$ and aliphatic protons are high, this is attributed to the presence of the decomposition product [1-(butadiene)][BAr$^F_4$] and recalcitrant NBA in the lattice.

^{31}P{^{1}H} solution NMR (CD_2Cl_2, 202 MHz, 193 K) δ: 97.5 (dd, J_{RhP} = 211 Hz, J_{PP} = 24 Hz), 89.9 (dd, J_{RhP} = 159 Hz, J_{PP} = 24 Hz).

^{13}C{^{1}H} solution NMR (CD_2Cl_2, 126 MHz, 193 K) δ: 161.4 (1:1:1:1 q, J_{BC} = 50 Hz, ipso-ArC), 134.3 (ortho-ArCH), 128.3 (q, J_{CF} = 32 Hz, meta-ArC), 124.1 (q, J_{CF} = 272 Hz, CF_3), 117.2 (para-ArCH), 90.2 (s, butene C=C), 36.8 (d, J_{PC} = 29 Hz, Cy-CH), 35.5 (d, J_{PC} = 25 Hz, Cy-CH), 29.9 (s, Cy-CH_2), 28.9 (s, Cy-CH_2), 28.7 (s, Cy-CH_2), 28.1 (s, Cy-CH_2), 26.1 (m, PCH_2), 25.5 (s, Cy-CH_2), 25.3 (s, Cy-CH_2), 10.9 (s, butene CH_3).

^{31}P{^{1}H} SSNMR (162 MHz, 298 K, 10 kHz spin rate) δ: 98.4 (br), 95.1 (br).

^{31}P{^{1}H} SSNMR (162 MHz, 158 K, 10 kHz spin rate) δ: 100.0 (br, $v_{1/2}$ = 512 Hz), 93.3 (br, $v_{1/2}$ = 418 Hz).

^{13}C{^{1}H} SSNMR (101 MHz, 298 K, 10 kHz spin rate) δ: 164.3 (BAr$^F_4$), 134.9 (BAr$^F_4$), 130.3 (BAr$^F_4$), 125.1 (BAr$^F_4$), 120.6 (BAr$^F_4$), 118.6 (BAr$^F_4$), 116.7 (BAr$^F_4$), 91.8 (br, butene C=C), 42-15 (multiple overlapping aliphatic resonances), 6.3 (br, butene agostic CH_3).

^{13}C{^{1}H} SSNMR (101 MHz, 158 K, 10 kHz spin rate) δ: 163.8 (BAr$^F_4$), 133.8 (BAr$^F_4$), 129.9 (BAr$^F_4$), 124.6 (BAr$^F_4$), 118.2 (BAr$^F_4$), 115.9 (BAr$^F_4$), 92.1 (butene C=C), 89.3 (butene C=C), 40-17 (multiple overlapping aliphatic resonances), 14.3 (butene non-agostic CH_3), 3.4 (butene agostic CH_3).

Mass Spec found: Under mass spectral conditions the only identifiable signal corresponds to **[1-(butadiene)][BAr$^F_4$]** (see S.2.4).

Elemental Analyses: The rate of decomposition of **[1-(butene)][BAr$^F_4$]** under an Ar atmosphere at room temperature prevented meaningful elemental analysis data being obtained.

S.2.3.3. Spectra of [1-(butene)][BAr$^F_4$]

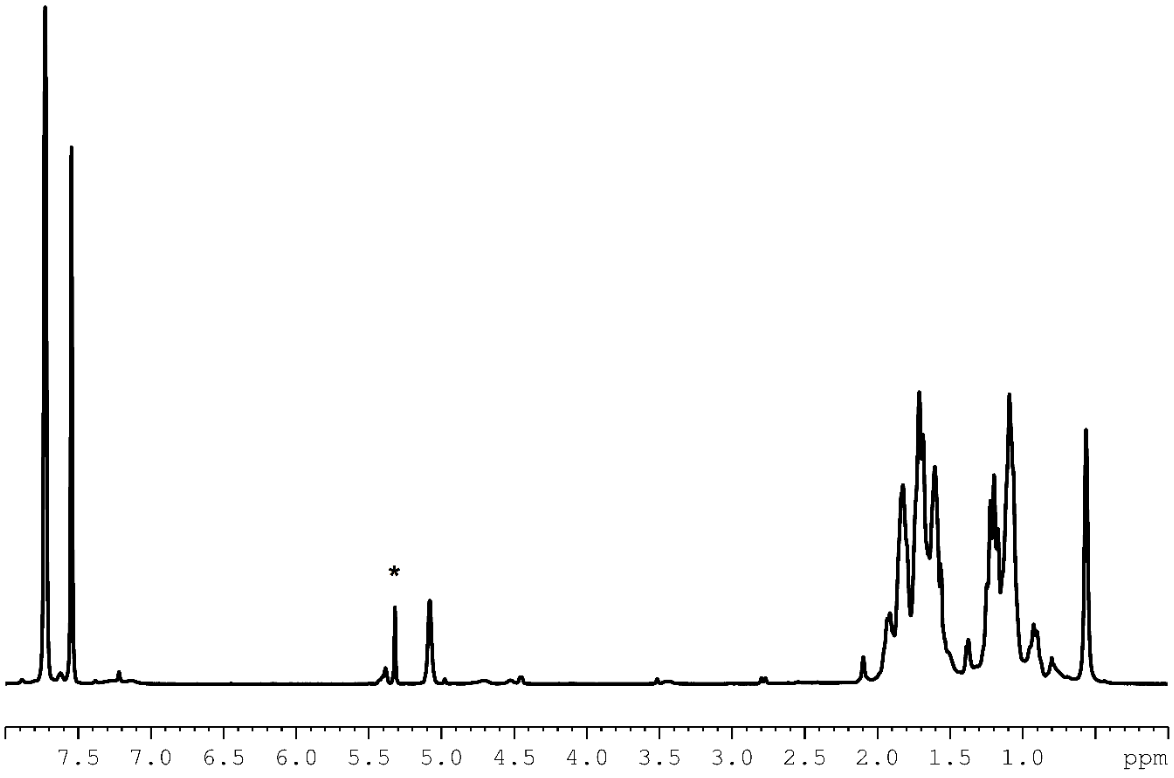

Figure S31: The solution ^{1}H NMR spectrum (CD_2Cl_2, 500 MHz, 193 K) of **[1-(butene)][BAr$^F_4$]**. The resonance marked * is due to residual protio solvent.

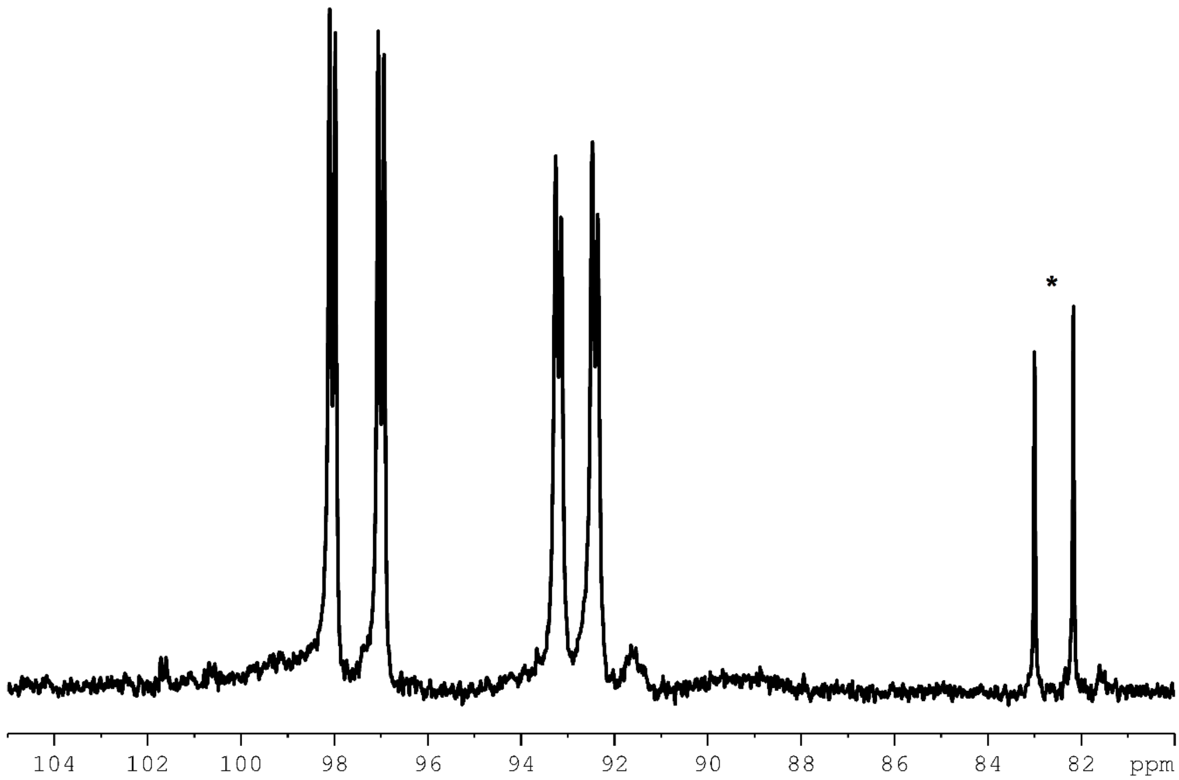

Figure S32: The solution $^{31}P\{^1H\}$ NMR spectrum (CD_2Cl_2, 202 MHz, 193 K) of **[1-(butene)][BAr$^F_4$]**. The resonance marked * is due to **[1-(butadiene)][BAr$^F_4$]**.

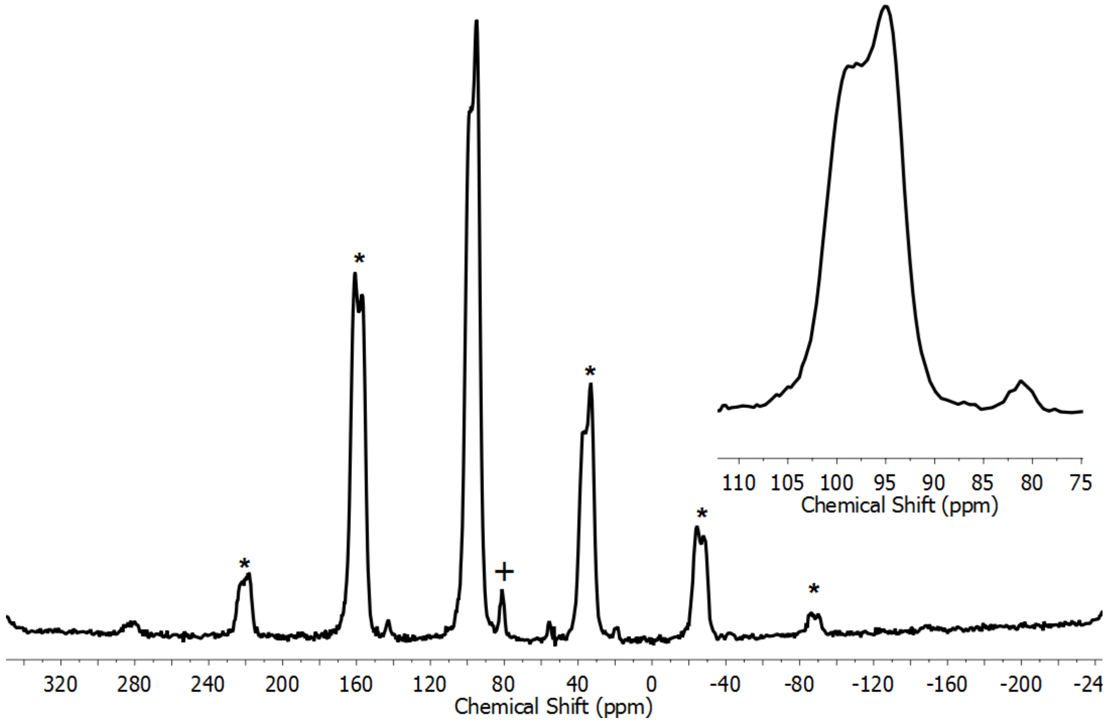

Figure S33: The $^{31}P\{^1H\}$ SSNMR spectrum (162 MHz, 298 K, 10 kHz spin rate) of **[1-(butene)][BAr$^F_4$]** prepared *in-situ*. The resonance marked + is due to **[1-(butadiene)][BAr$^F_4$]** growing in. The resonances marked * are due to spinning sidebands. The inset is a zoom of the central resonances.

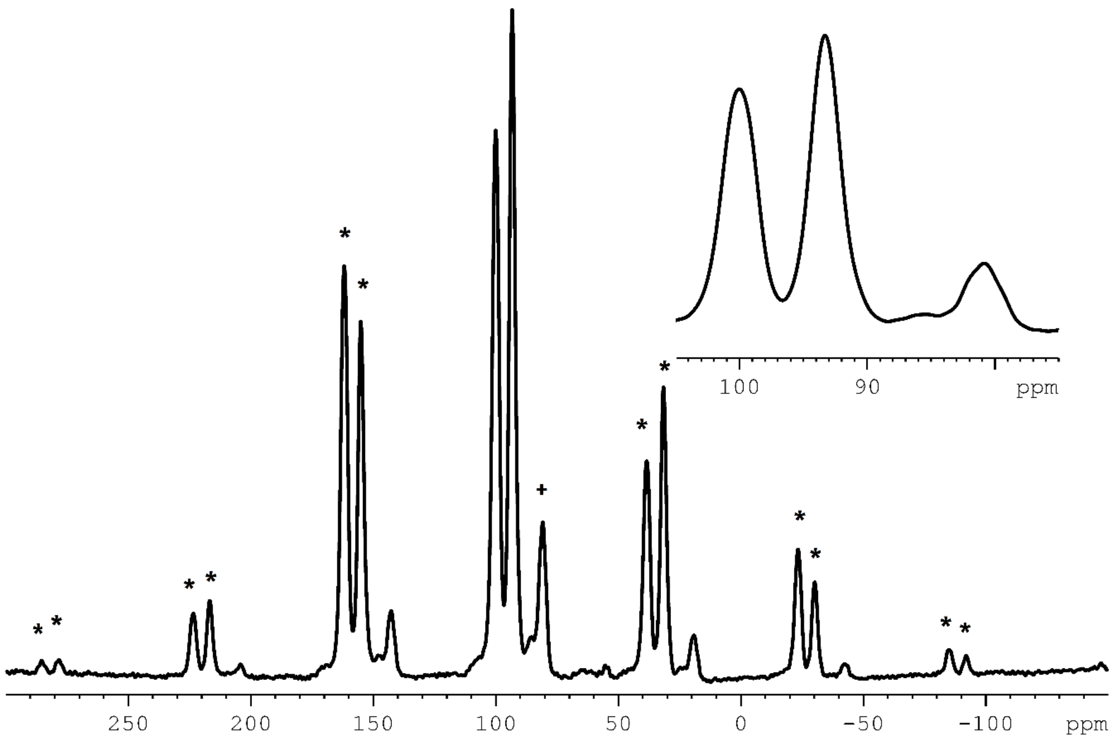

Figure S34: The $^{31}P\{^{1}H\}$ SSNMR spectrum (162 MHz, 158 K, 10 kHz spin rate) of **[1-(butene)][BAr$^{F}_{4}$]** prepared *ex-situ*. The resonances marked + are due to **[1-(butadiene)][BAr$^{F}_{4}$]**. The resonances marked * are due to spinning sidebands. The inset is a zoom of the central resonances.

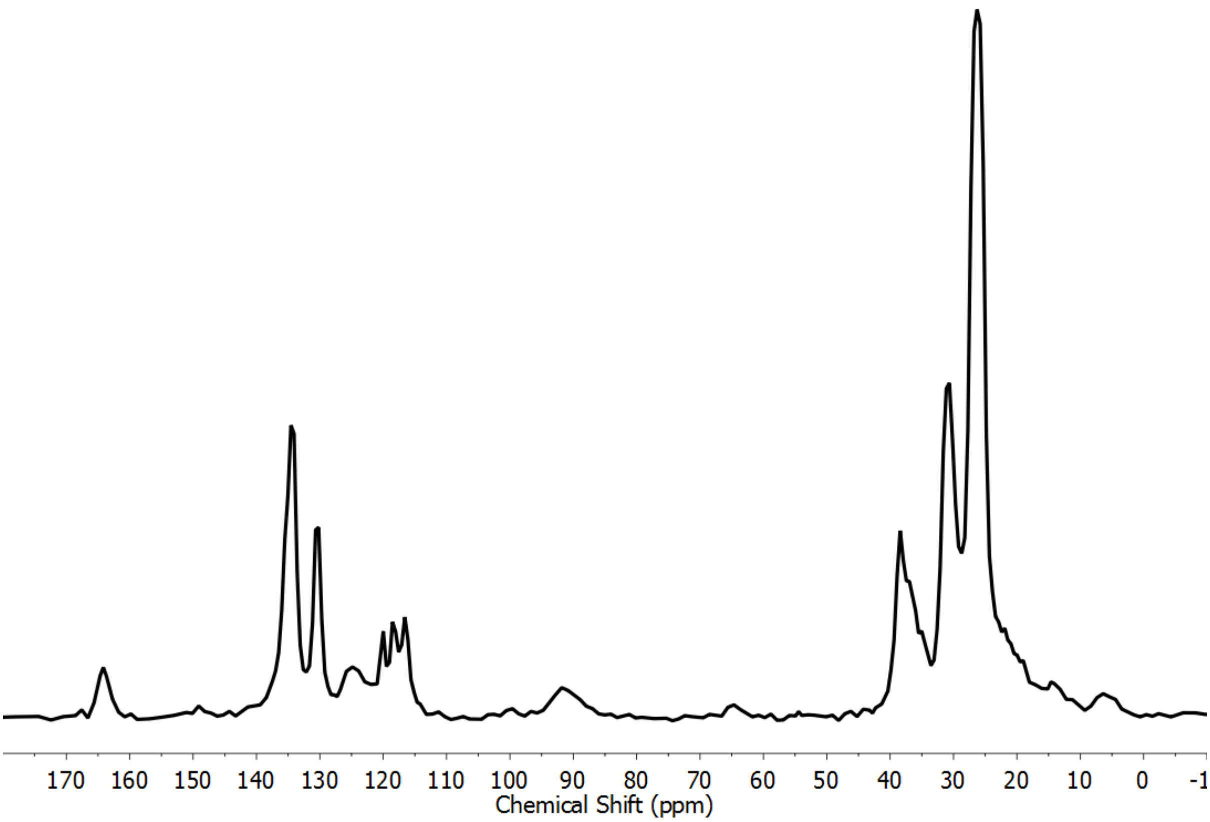

Figure S35: The $^{13}C\{^{1}H\}$ SSNMR spectrum (101 MHz, 298 K, 10 kHz spin rate) of **[1-(butene)][BAr$^{F}_{4}$]** prepared *in-situ*.

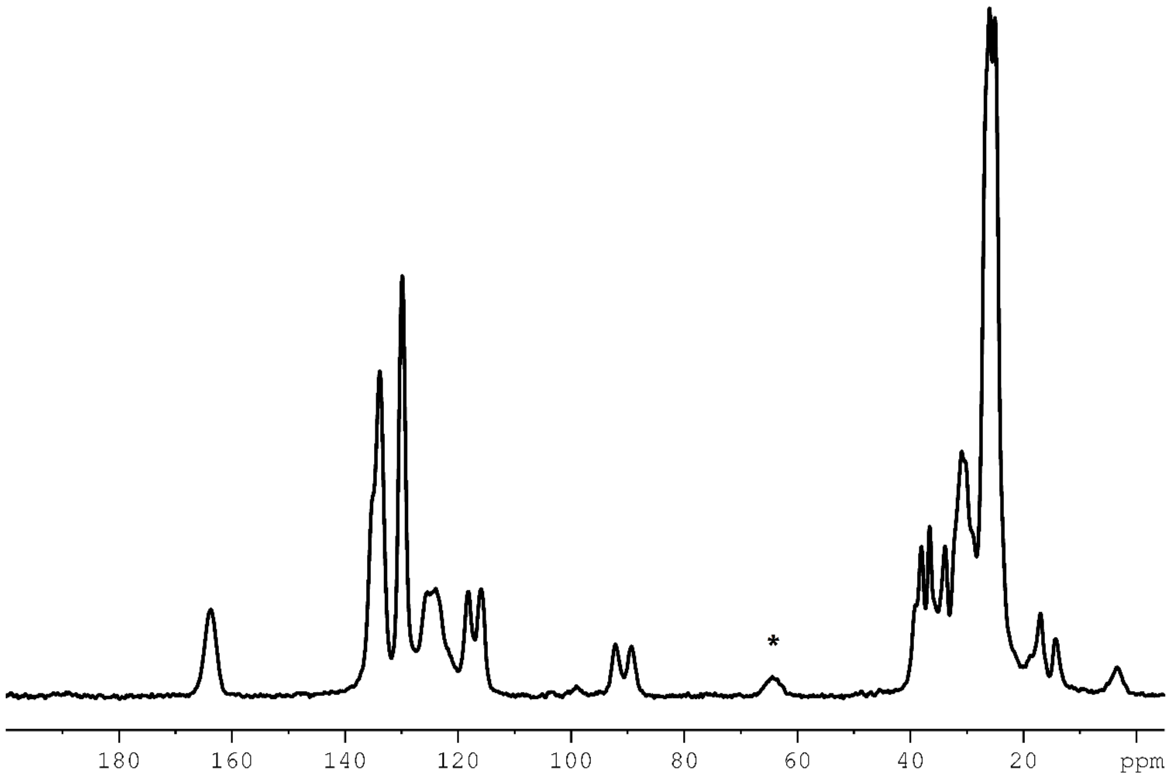

Figure S36: The $^{13}C\{^{1}H\}$ SSNMR spectrum (101 MHz, 158 K, 10 kHz spin rate) of **[1-(butene)][BAr$^{F}_{4}$]** prepared *ex-situ*. The resonance marked * is due to a spinning sideband.

S.2.3.4. Addition of D_2 to [1-(butene)][BAr$^F{}_4$]

[1-(butene)][BAr$^F{}_4$] was made *in-situ* by addition of 1-butene (15 psi, 298 K) to **[1-NBA][BAr$^F{}_4$]** (30 mg, 0.0202 mmol) in a high pressure NMR tube. This was allowed to stand for 5 min before subjection to vacuum to remove excess 1-butene gas and liberated NBA (cycled three time). D_2 gas was then added (15 psi, to form d_2-butane). The deuterated material was dissolved up in CH_2Cl_2 and $^2H\{^1H\}$ solution NMR was used to identify the locations of the deuterium atoms. This showed them to be in the 1,2-positions, implying the **[1-(1-butene)][BAr$^F{}_4$]**, although not observed to any significant amount spectroscopically, must deuterate quicker than **[1-(2-butene)][BAr$^F{}_4$]** as enabled by isomerization. Repeating the experiment but exposing the sample to 1-butene (15 psi, 298 K) for 2 h afforded the same result.

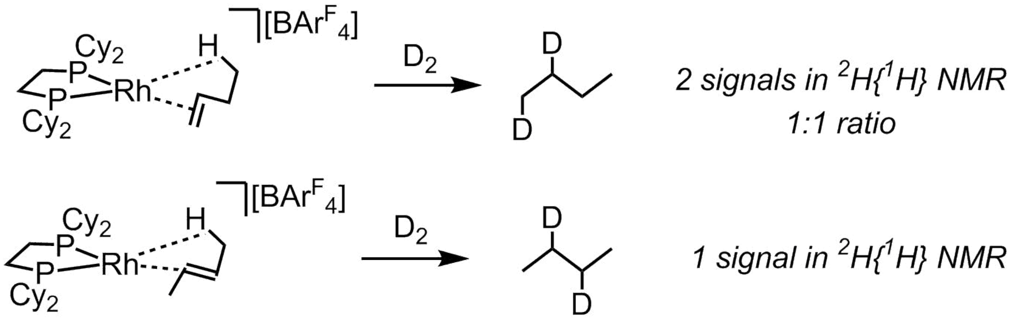

Scheme S2: The two options for the deuteration of butene from **[1-(butene)][BAr$^F{}_4$]**.

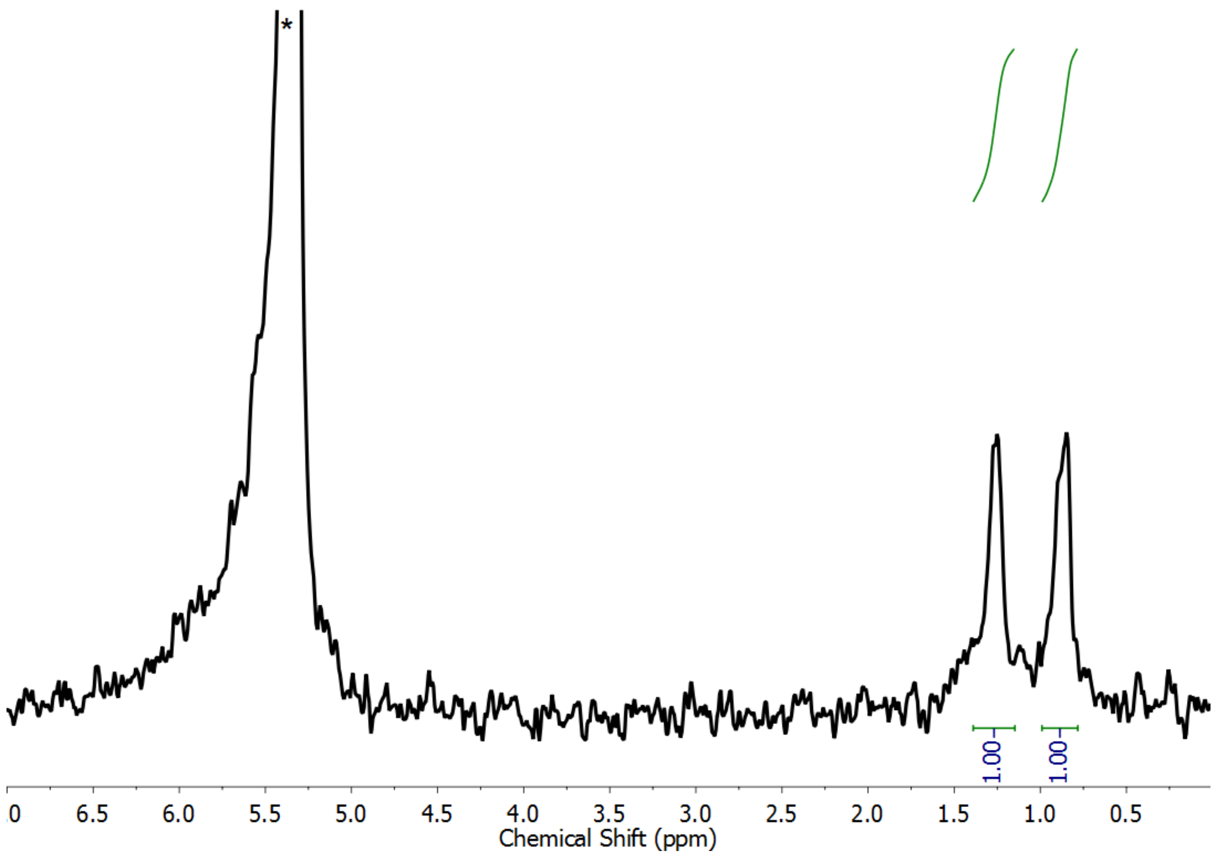

Figure S37: The solution $^{2}H\{^{1}H\}$ NMR spectrum (CH_2Cl_2, 61 MHz, 298 K) of the product of D_2 addition to the *in-situ* formed **[1-(butene)][BAr$^{F}_{4}$]** complex. The signal marked * is due to CD_2Cl_2 added for reference.

S.2.3.5. Characterization of the non-volatile component [Rh(dcpe)(CD_3CN)$_2$][BAr$^{F}_{4}$]

The yellow non-volatile component, presumably [Rh(dcpe)(CD_3CN)$_2$][BAr$^{F}_{4}$], was dissolved in CD_2Cl_2 and characterized by NMR spectroscopy.

^{1}H solution NMR (CD_2Cl_2, 500 MHz) δ: 7.72 (8H, s, o-BAr$^{F}_{4}$), 7.56 (4H, s, p-BAr$^{F}_{4}$), 2.09 (4H, br s, Cy-CH), 1.89-1.76 (20H, multiple overlapping aliphatic resonances, Cy-CH_2), 1.52 (4H, br d, $J_{PH} \approx 12$ Hz, PCH_2), 1.40-1.15 (20H, multiple overlapping aliphatic resonances, Cy-CH_2).

$^{31}P\{^{1}H\}$ solution NMR (CD_2Cl_2, 162 MHz) δ: 91.0 (d, J_{RhP} = 175 Hz).

Elemental analysis found (calc.): C 50.37% (50.42%), H 4.72% (4.91%), N 1.91% (1.90%).

S.2.4. Synthesis and characterization of [1-(butadiene)][BAr$^F_4$]

S.2.4.1. Solution synthesis of [1-(butadiene)][BAr$^F_4$]

A sample of **[1-$F_2C_6H_4$][BAr$^F_4$]** (50 mg, 0.0333 mmol) was dissolved up in CH_2Cl_2 (25 mL) in a J. Young's flask (c. 100 mL). The solution was freeze-pump-thawed degassed three times before 1-butene gas (15 psi, 298 K) was added. The solution was allowed to stir for 16 hours, before volatiles were removed *in vacuo*. The resultant burgundy solid was washed with pentane, before being re-dissolved in the minimum volume of CH_2Cl_2 and filtered *via* cannula before being layered with pentane. Though this yielded crystalline material they were found to not be suitable for analysis by single crystal X-ray diffraction (all crystals mounted were found to be severely twinned or not-single). However, the material was shown to be pure by solution ^1H and $^{31}P\{^1H\}$ NMR spectroscopy. Isolated yield: 42 mg (0.0291 mmol, 87%).

S.2.4.2. Solid state synthesis and characterization of [1-(butadiene)][BAr$^F_4$]

To an orange sample of crystalline **[1-NBA][BAr$^F_4$]** (50 mg, 0.0333 mmol) in a J. Young's flask (c. 100 mL), 1-butene gas (15 psi, 298 K) is added and left standing for 48 hours. Over this time the sample goes a deep burgundy color. Though crystallinity appears to be retained considerable data loss occurs (for single crystal X-ray diffraction), especially at high angle, and even getting absolute connectivity is not possible. $^{31}P\{^1H\}$ solution NMR (CD_2Cl_2) on the dissolved sample showed the product to be formed quantitatively and to be chemically identical to that produced by solution route. Gas phase ^1H NMR spectroscopic analyses of the reaction head space (see figure S43) showed concomitant formation butane and 2-butene as a result of the formation of **[1-(butadiene)][BAr$^F_4$]** (as discussed in detail in section S4).

^1H solution NMR (CD_2Cl_2, 500 MHz, 298 K) δ: 7.72 (8H, s, o-BAr$^F_4$), 7.56 (4H, s, p-BAr$^F_4$), 5.47 (2H, br t, C2/C3, J_{HH} ≈ 9 Hz), 4.51 (2H, br d, C1/C4, J_{HH} = 6 Hz), 2.83 (2H, d, C1/C4, J_{HH} = 14 Hz), 2.25-0.90 (48H, multiple overlapping aliphatic resonances).

$^{31}P\{^1H\}$ solution NMR (CD_2Cl_2, 202 MHz, 298 K) δ: 82.0 (d, J_{RhP} = 169 Hz).

$^{31}P\{^1H\}$ SSNMR (162 MHz, 298 K, 10 kHz spin rate) δ: 81.0 (asym. br.).

$^{13}C\{^1H\}$ SSNMR (101 MHz, 298 K, 10 kHz spin rate) δ: 164.3 (BAr$^F_4$), 134.4 (BAr$^F_4$), 130.3 (BAr$^F_4$), 125.1 (BAr$^F_4$), 118.6 (BAr$^F_4$), 116.7 (BAr$^F_4$), 103.5 (butadiene), 99.6 (butadiene), 87.8 (butadiene), 63.2 (butadiene), 42-15 (multiple overlapping aliphatic resonances).

Mass Spec found (calc.): 579.2733 (579.2750). Note – considerable signal (with appropriate isotopic distribution) at m/z = $[(Cy_2PCH_2CH_2PCy_2)Rh(C_2H_4)]^+$; $[(Cy_2PCH_2CH_2PCy_2)Rh(C_6H_{10})]^+$; $[(Cy_2PCH_2CH_2PCy_2)Rh(C_7H_{12})]^+$.

Elemental analysis found (calc.): C 51.50% (51.61%), H 4.53% (4.61%).

S.2.4.3. Spectra of [1-(butadiene)][BAr^F$_4$]

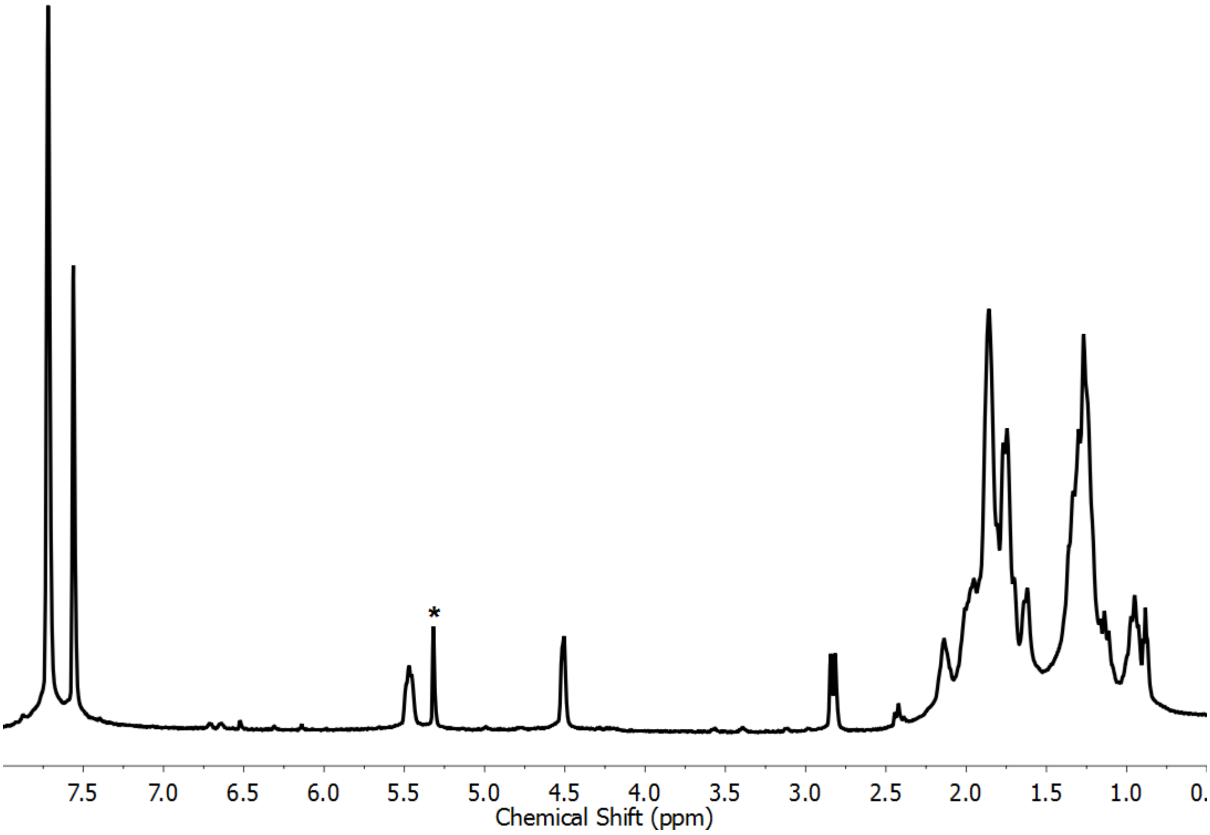

Figure S38: The solution ^{1}H NMR spectrum (CD_2Cl_2, 500 MHz, 298 K) of **[1-(butadiene)][BAr^F$_4$]**. The peak labelled * is residual protio solvent. This sample was prepared by the solid state route.

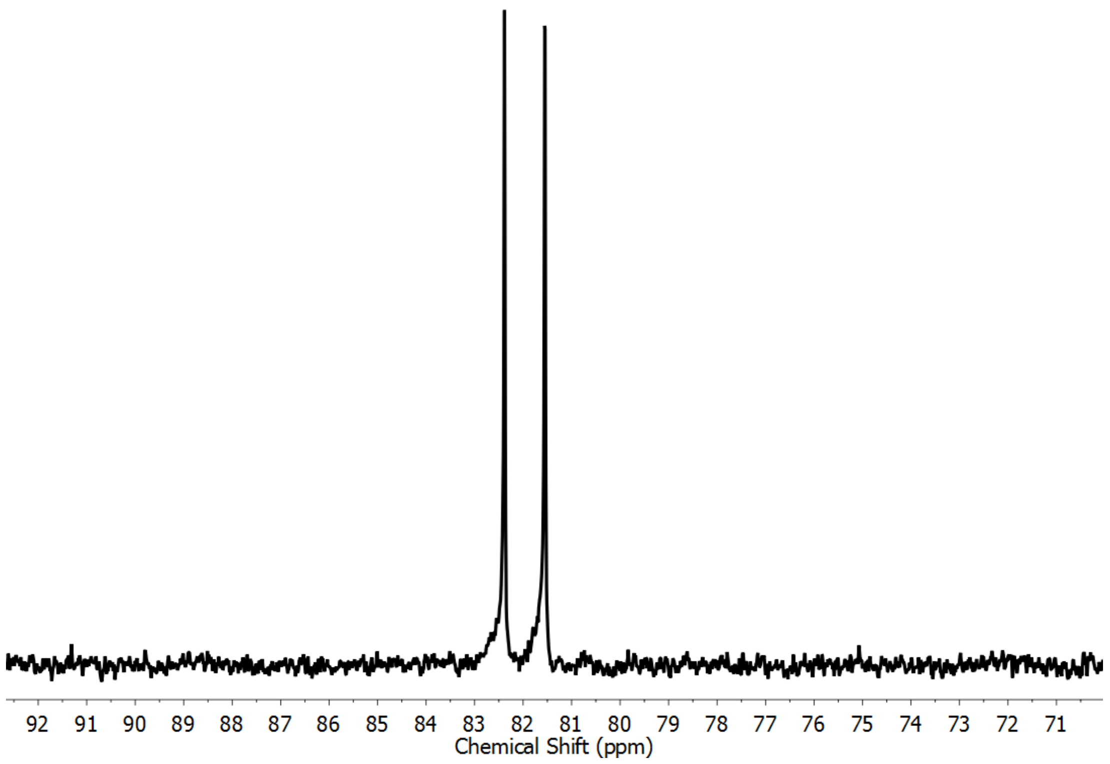

Figure S39: The solution $^{31}P\{^{1}H\}$ NMR spectrum (CD_2Cl_2, 202 MHz, 298 K) of **[1-(butadiene)][BAr$^F_4$]**. This sample was prepared by solid state route.

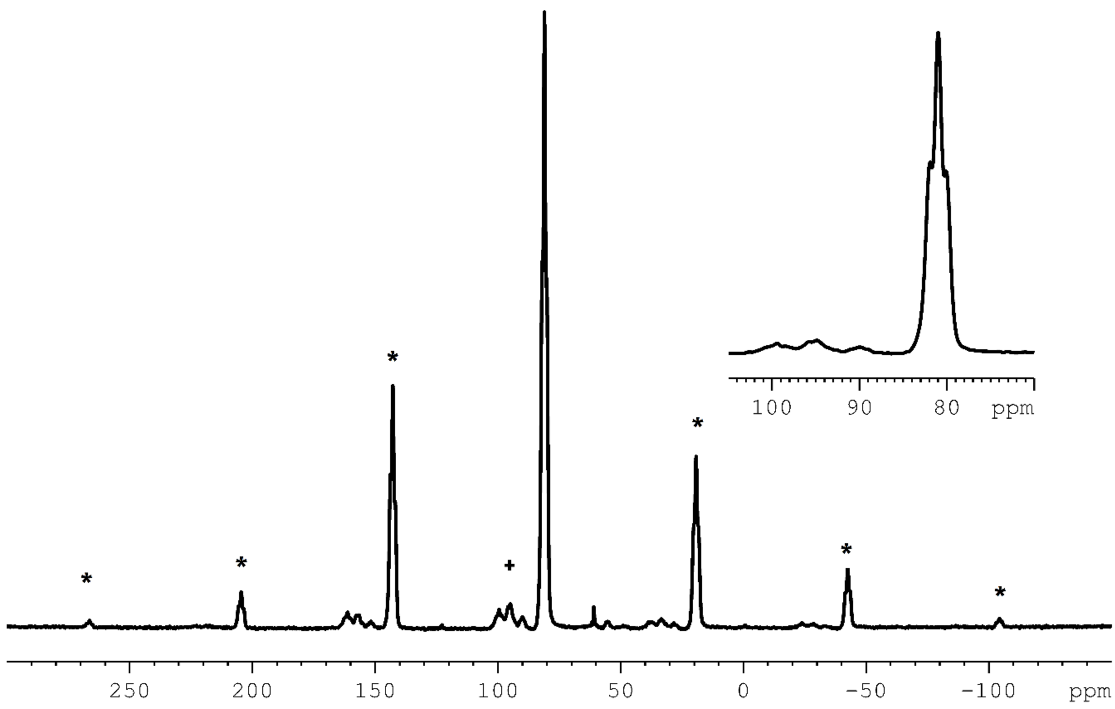

Figure S40: The $^{31}P\{^{1}H\}$ SSNMR spectrum (162 MHz, 298 K, 10 kHz spin rate) of **[1-(butadiene)][BAr$^F_4$]** prepared by the *ex-situ* solid state route. The resonances marked * are due to spinning side-bands. The resonances marked + are due to impurities, either **[1-(butene)][BAr$^F_4$]** (the two downfield resonances at δ c. 100 ppm and 95 ppm) or **[1-BAr$^F_4$]** (δ c. 90 ppm).

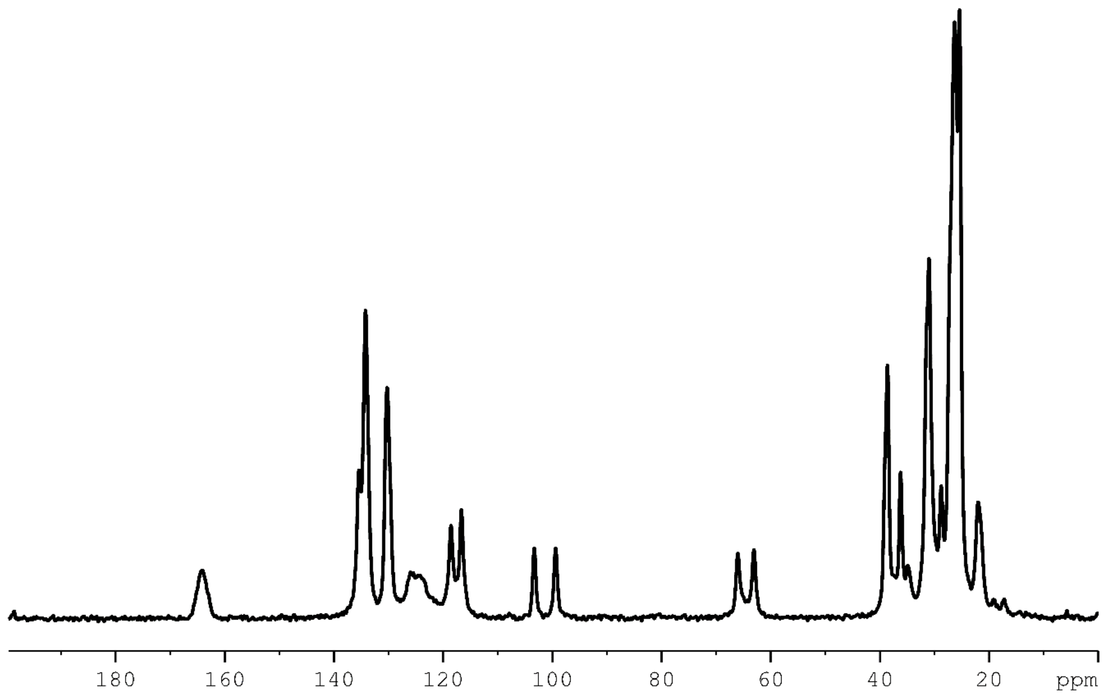

Figure S41: The $^{13}C\{^1H\}$ SSNMR spectrum (101 MHz, 298 K, 10 kHz spin rate) of **[1-(butadiene)][BAr$^F{}_4$]** prepared by the *ex-situ* solid state route.

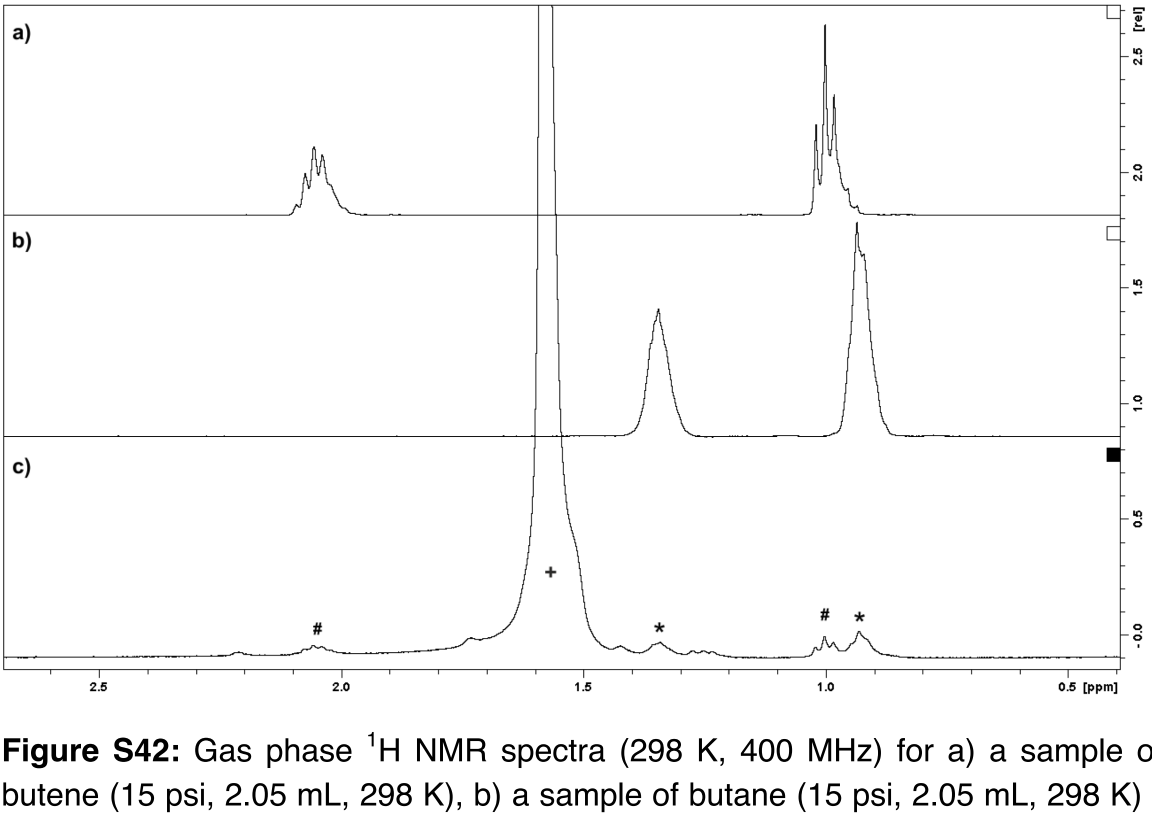

Figure S42: Gas phase ^1H NMR spectra (298 K, 400 MHz) for a) a sample of 1-butene (15 psi, 2.05 mL, 298 K), b) a sample of butane (15 psi, 2.05 mL, 298 K) and c) head space mixture for the reaction of **[1-NBA][BAr$^F{}_4$]-large** (10 mg) with 1-butene (15 psi, 2.05 mL, 298 K) quantitatively forming **[1-(butadiene)][BAr$^F{}_4$]** after 48 h. The resonances marked $^{\#}$, * and $^{+}$ are due to remaining 1-butene, and newly

formed butane and 2-butene, respectively. Spectra are internally referenced relative to reported gas phase data.[S4]

S.2.5. Characterization of [1-$(CO)_2$][BAr^F_4]

^{1}H solution NMR (CD_2Cl_2, 500 MHz, 298 K) δ: 7.72 (8H, s, o-BAr^F_4), 7.56 (4H, s, p-BAr^F_4), 2.17-2.07 (8H, multiple overlapping aliphatic resonances), 1.97-1.73 (20H, multiple overlapping aliphatic resonances), 1.41-1.15 (20H, multiple overlapping aliphatic resonances).

^{31}P{^{1}H} solution NMR (CD_2Cl_2, 202 MHz, 298 K) δ: 85.1 (d, J_{RhP} = 117 Hz).

Mass Spec found (calc.): 581.2215 (581.2179).

Elemental analysis found (calc.): C 49.97% (49.88%), H 4.15% (4.19%).

IR Spectroscopy (CD_2Cl_2 solution): CO stretches 2094.3, 2049.8 cm^{-1}.

IR Spectroscopy (ATR): CO stretches 2093.6, 2049.3 cm^{-1}.

S.3. Crystallographic and refinement data

S.3.1. Crystal structure determinations

Single crystal X-ray diffraction data for all samples were collected as follows: a typical crystal was mounted on a MiTeGen Micromounts using perfluoropolyether oil and cooled rapidly to 150 K in a stream of nitrogen gas using an Oxford Cryosystems Cryostream unit.[S8] Data were collected with an Agilent SuperNova diffractometer (Cu K_α radiation, λ = 1.54180 Å). Raw frame data were reduced using CrysAlisPro.[S9-10] The structures were solved using SuperFlip[S11] or SHELXT[S12] and refined using full-matrix least squares refinement on all F^2 data using the CRYSTALS program suite[S13-14] or with SHELXL-14[S15] using the GUI OLEX2[S16]. In general distances and angles were calculated using the full covariance matrix. Dihedral angles were calculated using PLATON.[S17] In general some CF_3 groups in the $[BAr^F{}_4]^-$ anions refined to give highly prolate displacement ellipsoids. For each structure a split site model was used. Restraints were used to maintain sensible geometries and displacement parameters. Further comments regarding individual crystal structures can be found in Section S.3.4.

S.3.2. Selected crystallographic and refinement data

Table S1: Selected crystallographic and refinement data for **[1-(ethene)$_2$][BAr^F$_4$]-*Oct*** and **[1-(ethene)$_2$][BAr^F$_4$]-*Hex***

	**[1-(ethene)$_2$][BAr^F$_4$]-*Oct***	**[1-(ethene)$_2$][BAr^F$_4$]-*Hex***
Chemical formula	$C_{62}H_{68}BF_{24}P_2Rh$	$C_{62}H_{68}BF_{24}P_2Rh$
Formula weight	1444.84	1444.84
Temperature (K)	150	150
Crystal system	monoclinic	hexagonal
Space group	*C*2/*c*	$P6_322$
a (Å)	19.2187(18)	25.8617(3)
b (Å)	17.1982(18)	25.8617(3)
c (Å)	19.999(3)	20.3020(3)
α (deg)	90	90
β (deg)	90.738(12)	90
γ (deg)	90	120
V (Å^3)	6609.7(14)	11759.4(3)
Z	4	6
ρ(calcd) (g cm^{-3})	1.452	1.224
μ (mm^{-1})	3.490	2.943
Reflections collected	9108	83387
Unique reflections	5540	8209
Restraints / Parameters	102 / 481	456 / 464
R_{int}	0.042	0.121
R_1 [$I > 2\sigma(I)$]	0.1072	0.0666
wR_2 [$I > 2\sigma(I)$]	0.2509	0.1776
GooF	0.9981	0.9722
Residual electron density (e Å^{-3})	1.69 / -1.04	1.05 / -0.77
CCDC no.	1539832	1539833

Table S2: Selected crystallographic and refinement data for **[1-(propene)$_2$][BAr$^F_4$]**, **[1-(propene)][BAr$^F_4$]** and **[1-(butene)][BAr$^F_4$]**.

	**[1-(propene)$_2$][BAr$^F_4$]**	**[1-(propene)][BAr$^F_4$]**	**[1-(butene)][BAr$^F_4$]**
Chemical formula	$C_{64}H_{72}BF_{24}P_2Rh$	$C_{61}H_{66}BF_{24}P_2Rh$	$C_{62}H_{68}BF_{24}P_2Rh$
Formula weight	1472.87	1430.81	1444.82
Temperature (K)	150	150	150
Crystal system	triclinic	triclinic	monoclinic
Space group	$P\bar{1}$	$P\bar{1}$	*C*2/*c*
a (Å)	13.4908(4)	12.7894(7)	19.1979(10)
b (Å)	14.2693(4)	12.8961(7)	17.1549(15)
c (Å)	18.1051(4)	19.8711(8)	19.9732(12)
α (deg)	98.549(2)	91.256(4)	90
β (deg)	102.097(2)	90.745(4)	90.310(6)
γ (deg)	94.411(2)	96.863(4)	90
V (Å^3)	3348.94(16)	3252.74(13)	6577.8(8)
Z	2	2	4
ρ(calcd) (g cm^{-3})	1.461	1.461	1.459
μ (mm^{-1})	3.455	3.540	3.507
Reflections collected	36956	27129	18456
Unique reflections	13869	12893	6753
Restraints / Parameters	612 / 1028	1978 / 1052	237 / 483
R_{int}	0.0305	0.054	0.0290
R_1 [$I > 2\sigma(I)$]	0.0552	0.1277	0.1019
wR_2 [$I > 2\sigma(I)$]	0.1417	0.3264	0.2487
GooF	1.043	0.9616	1.104
Residual electron density (e Å^{-3})	2.248 / -0.938	3.94 / -1.57	1.972 / -1.630
CCDC no.	1539834	1539835	1539836

S.3.3. Crystal structure of [1-(propene)$_2$][BAr^F$_4$]

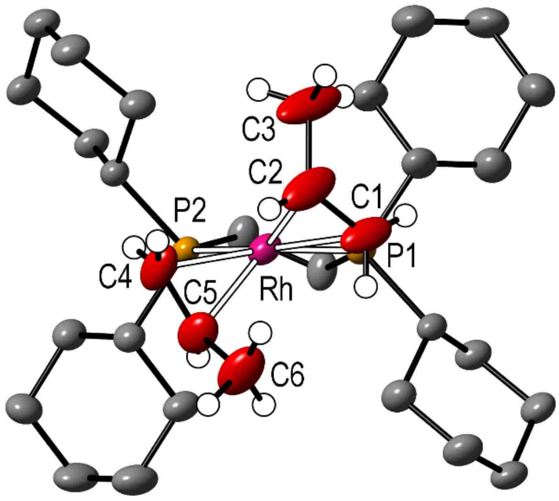

Figure S43: Molecular structure of **[1-(propene)$_2$][BAr^F$_4$]** with thermal ellipsoids at 35% probability. Hydrogen atoms of the phosphine ligand and one disordered component of both propene ligands have been omitted for clarity.

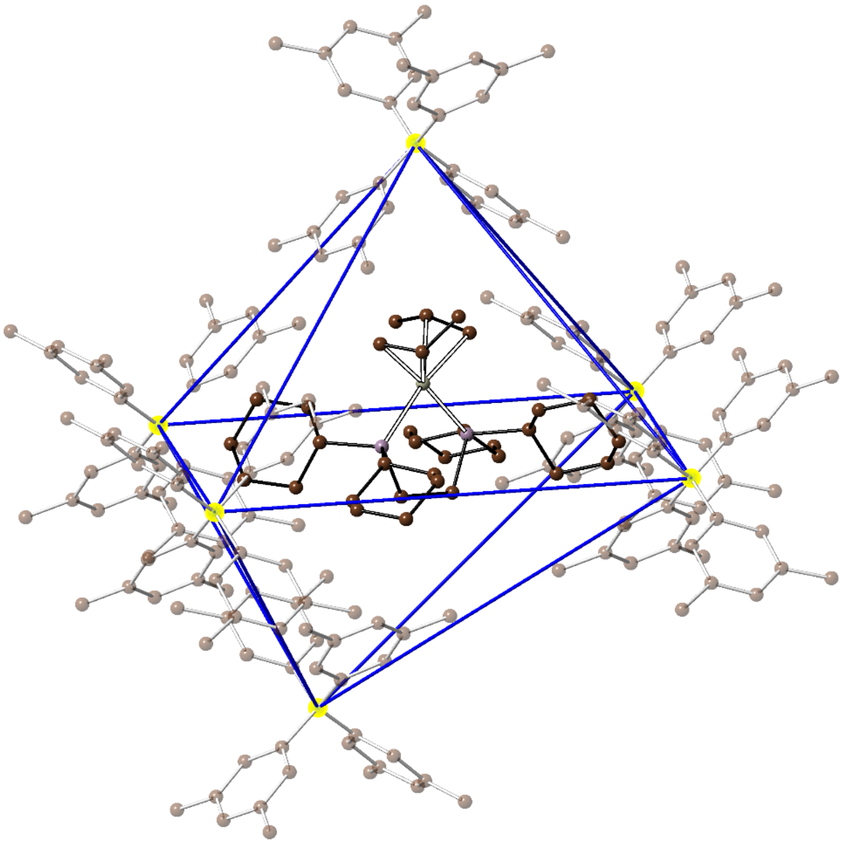

Figure S44: Packing arrangement of the [BAr^F$_4$]$^-$ anions around the units of [1-(propene)$_2$]$^+$ in the crystal structure of **[1-(propene)$_2$][BAr^F$_4$]**. The [BAr^F$_4$]$^-$ anions occupy the positions for a distorted octahedron.

S.3.4. Additional details on the crystal structures

Comments for the crystal structures of [1-(ethene)$_2$][BAr^F$_4$]-*Oct* and [1-(ethene)$_2$][BAr^F$_4$]-*Hex*: It is noted that the structure of **[1-(ethene)$_2$][BAr^F$_4$]-*Oct*** has an elevated R-factor, as well as a low full θ_{max} value. This is primarily due to a loss in high angle data – which is rationalized by the three consecutive single-crystal to single-crystal synthetic steps during its preparation; thus, increasing strain on the lattice.

For **[1-(ethene)$_2$][BAr^F$_4$]-*Hex*** no such loss of data is presented as it was ultimately crystallized from solution. The PLATON-checkcif crystallographic report corresponding to the molecular structure of **[1-(ethene)$_2$][BAr^F$_4$]-*Hex*** contains an A alert (PLAT602_ALERT_2_A VERY LARGE Solvent Accessible VOID(S) in Structure ! Info) due to the very large voids due to unidentified solvent molecules of crystallization (presumably pentane) present in the lattice. This is noted and commented upon in the full text and in the associated CIF.

Comments for the crystal structure of [1-(propene)][BAr^F$_4$] Similarly to **[1-(ethene)$_2$][BAr^F$_4$]-*Oct*** there is a somewhat elevated R-factor and a low θ_{max} value attributed to the loss of high angle data due to crystal quality degrading due to sequential single-crystal to single-crystal transformations. This has been accordingly addressed in the corresponding CIF.

Comments for the crystal structure of [1-(propene)$_2$][BAr^F$_4$]: Both coordinated propene molecules are disordered over two sites. The disorder model was successful conjoined. Distance restraints were applied to the atoms of the propene molecules. Displacement similarity and vibrational restraints were additionally applied to the atoms propene molecules.

Comments for the crystal structure of [1-(butene)][BAr^F$_4$]: Similarly to **[1-(propene)][BAr^F$_4$]** there is a somewhat elevated R-factor due to the loss of high angle data due to crystal quality degrading due to sequential single-crystal to single-crystal transformation. The coordinated cis-2-butene was modelled as disordered over a crystallographic 2-fold axis. Displacement similarity and vibrational restraints were additionally applied to the atoms of the 2-butene.

S.4. Solid state molecular organometallic catalyst (SMOM-cat) data

S.4.1. Catalytic isomerization experiments of 1-butene to 2-butene

S.4.1.1. General experimental considerations for the gas phase catalytic isomerization of 1-butene to 2-butene

Six SMOM-catalyst were investigated in total, **[1-NBA][BAr$^F_4$]-large**, **[1-NBA][BAr$^F_4$]-crushed**, **[1-(ethene)$_2$][BAr$^F_4$]-*Oct*-large** **[1-(ethene)$_2$][BAr$^F_4$]-*Oct*-crushed**, **[1-(ethene)$_2$][BAr$^F_4$]-*Hex*-large, [1-(ethene)$_2$][BAr$^F_4$]-*Hex*-crushed**. Large samples refer to single crystals of the corresponding catalyst of an approximate edge length of 0.2mm in all the cases. Crushed samples refer to crystalline samples of the corresponding catalyst which have been finely pulverized prior to use. Crystalline **[1-NBA][BAr$^F_4$]-large** was prepared by following the previously reported solid state synthetic route.[S1]

All the catalysts (2.5 mg each) were loaded to an appropriate J Young high pressure NMR tube (2.05 mL) in an argon-filled box and then sealed with a Teflon stopcock. The NMR tubes were then transferred and fitted to a Schlenk using a custom-built glass T-piece adaptor (see Figure S2) with appropriate connecting tube. This T-piece adaptor allowed us to exposure the J Young high pressure NMR tube (containing the catalyst) to vacuum-argon on one side and the reagent 1-butene gas on the other side. Once the NMR tube was fitted to the T-piece, the system was sequentially, evacuated (< 1 x 10^{-3} mbar) and refilled with argon three times, evacuated and refilled with 1-butene (15 psi, 298 K) three times, before the NMR tube containing the catalyst was evacuated for 30 sec under dynamic vacuum (< 1 x 10^{-3} mbar). After this time, the NMR tube containing the catalyst was refilled with 1-butene gas (15 psi) as a timer was simultaneously started, then quickly sealed and transferred to a NMR spectrometer which was set up *a priori* by locking and shimming to a sample of C_6D_6 in a similar bore NMR tube. Then, the first gas phase ^{1}H NMR spectrum of the NMR tube head space was acquired as quickly as possible (first catalytic data point typically within a reaction time of 50-60 sec, the exact time for each run was noted) and then a batch of sequential ^{1}H NMR experiments was set up until the catalysis reached the thermodynamic equilibrium conditions for the isomerization (mixture of 1-butene:2-butene of 3:97, approximately). For **[1-NBA][BAr$^F_4$]-large** and **[1-(ethene)$_2$][BAr$^F_4$]-*Oct*-large**, 5 and 6 hr are needed, respectively, to reach the thermodynamic equilibrium conditions for the isomerization of 1-butene to 2-butene; therefore, for these catalysts 90% conversion was used to calculate TON and TOF. For the rest of catalyst screened the thermodynamic equilibrium conditions are reached within 20 to 30 min, depending on the catalyst used (see S.4.1.2 to S.4.1.7).

The extent of conversion was measured by the comparison of the integral of the two alkene CH resonances of 2-butene and the alkyl CH_2 resonance of 1-butene. These have been previously shown to be comparable by gas phase NMR spectroscopy.[S4]

TON and TOF are calculated assuming that all sites are equally catalytically active, and are therefore, a minimum number. Intuitively surface sites would be more active than those at the center of the bulk by a simple mass transit argument. When adding the 1-butene gas to the sample extreme care must be taken to expose the sample to the minimum amount of vacuum possible (typically 30 sec), as it was found extended vacuum caused for catalytic activity to fall.

S.4.1.2. Isomerization catalytic data for [1-NBA][BAr$^F{}_4$]-large

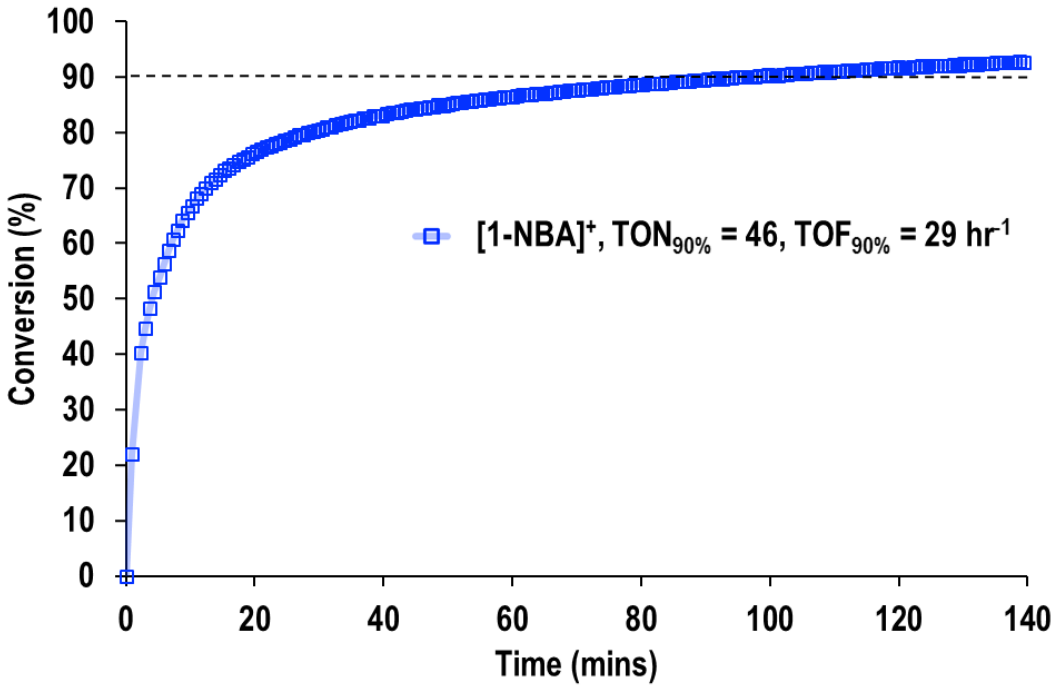

Figure S45: Data for the catalytic isomerization of 1-butene to 2-butene using **[1-NBA][BAr$^F{}_4$]-large** as measured by gas phase ^{1}H NMR spectroscopy. The dashed line indicates 90% conversion. Thermodynamic equilibrium conditions for the isomerization (1-butene:2-butene. 97:3%) are reached within 5 hr of catalysis.

S.4.1.3. Isomerization catalytic data for [1-(ethene)$_2$][BAr$^F_4$]-*Oct*-large

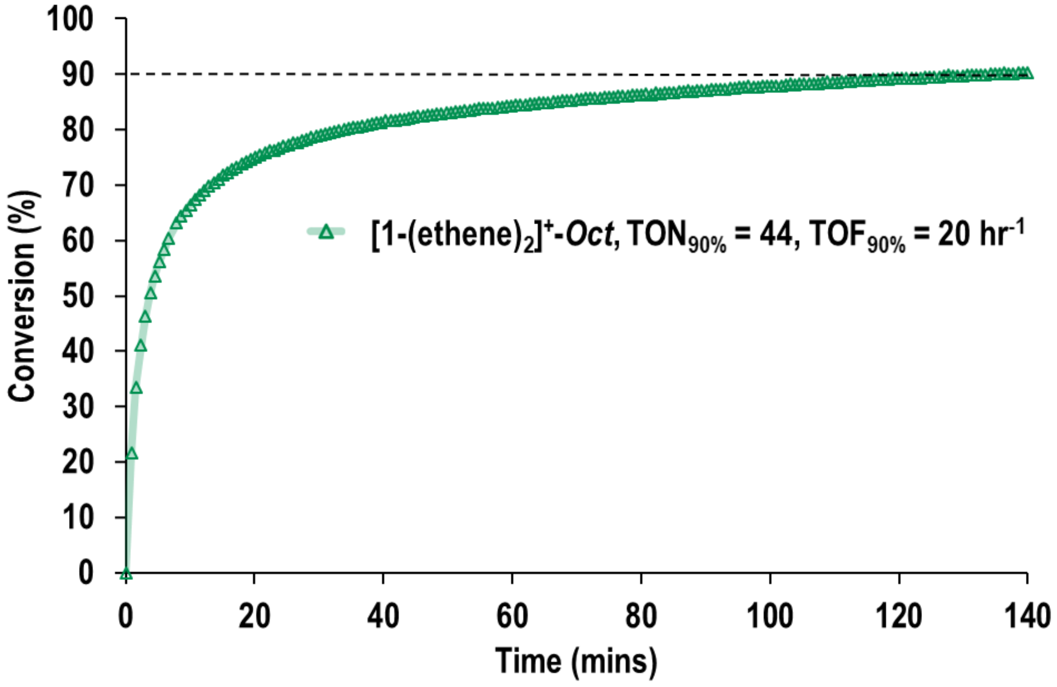

Figure S46: Data for the catalytic isomerization of 1-butene to 2-butene using **[1-(ethene)$_2$][BAr$^F_4$]-*Oct*-large** as measured by gas phase ^{1}H NMR spectroscopy. The dashed line indicates 90% conversion. Thermodynamic equilibrium conditions for the isomerization (1-butene:2-butene. 97:3%) are reached within 6 hr of catalysis.

S.4.1.4. Isomerization catalytic data for [1-(ethene)$_2$][BAr$^F{}_4$]-*Hex*-large

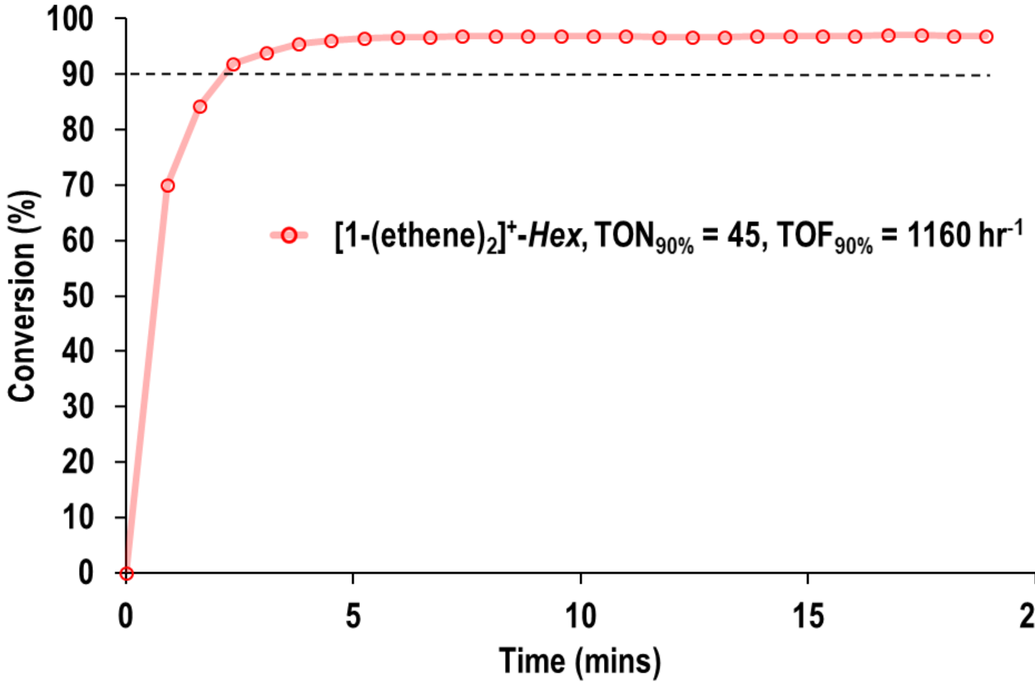

Figure S47: Data for the catalytic isomerization of 1-butene to 2-butene using **[1-(ethene)$_2$][BAr$^F{}_4$]-*Hex*-large** as measured by gas phase ^{1}H NMR spectroscopy. The dashed line indicates 90% conversion. Thermodynamic equilibrium conditions for the isomerization (1-butene:2-butene. 97:3%) are reached within 6 min of catalysis.

S.4.1.5. Isomerization catalytic data for [1-NBA][BAr$^F{}_4$]-crushed

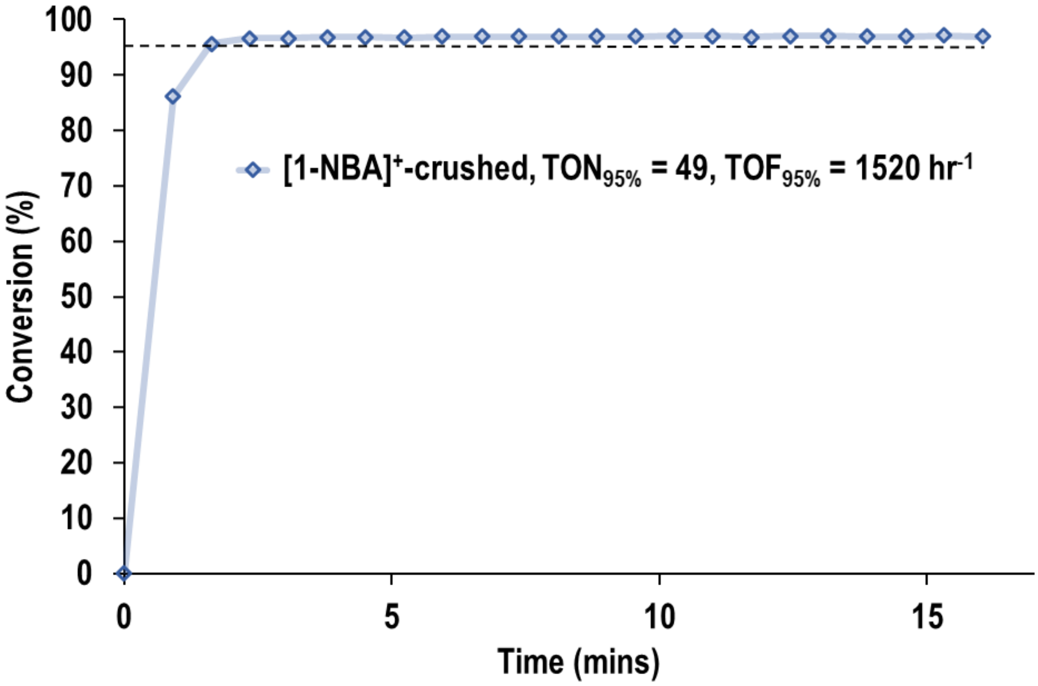

Figure S48: Data for the catalytic isomerization of 1-butene to 2-butene using **[1-NBA][BAr$^F{}_4$]-crushed** as measured by gas phase ^{1}H NMR spectroscopy. The dashed line indicates 95% conversion. Thermodynamic equilibrium conditions for the isomerization (1-butene:2-butene. 97:3%) are reached within 3 min of catalysis.

S.4.1.6. Isomerization catalytic data for [1-(ethene)$_2$][BAr$^F{}_4$]-*Oct*-crushed

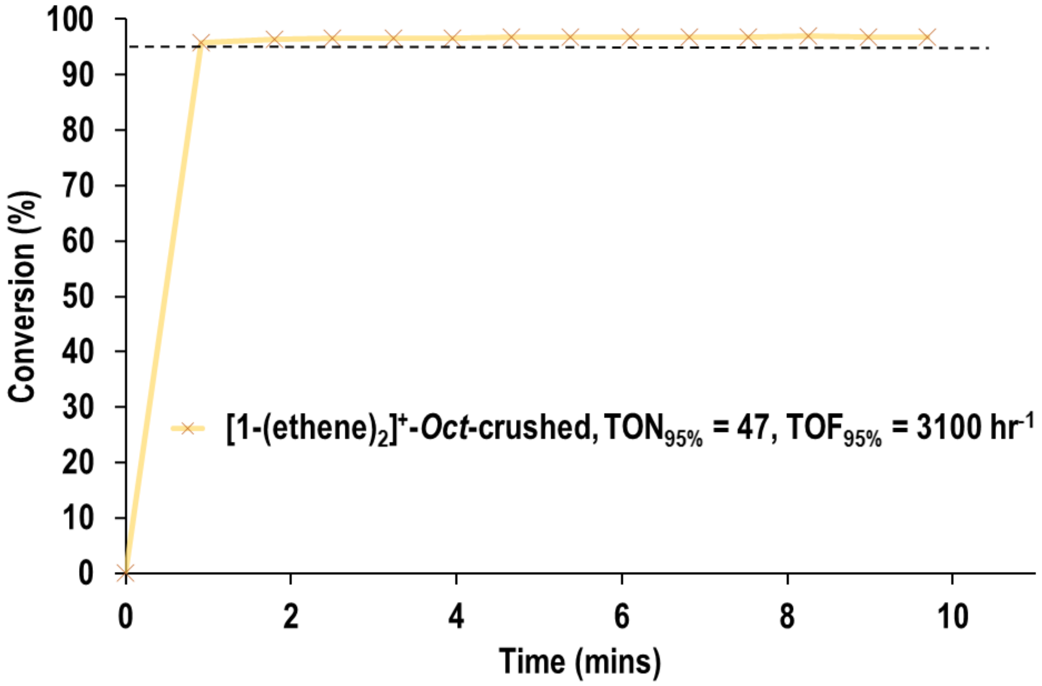

Figure S49: Data for the catalytic isomerization of 1-butene to 2-butene using **[1-(ethene)$_2$][BAr$^F{}_4$]-*Oct*-crushed** as measured by gas phase ^{1}H NMR spectroscopy. The dashed line indicates 95% conversion. Thermodynamic equilibrium conditions for the isomerization (1-butene:2-butene. 97:3%) are reached within 3.5 mins of catalysis.

S.4.1.7. Isomerization catalytic data for [1-(ethene)$_2$][BAr$^F{}_4$]-*Hex*-crushed

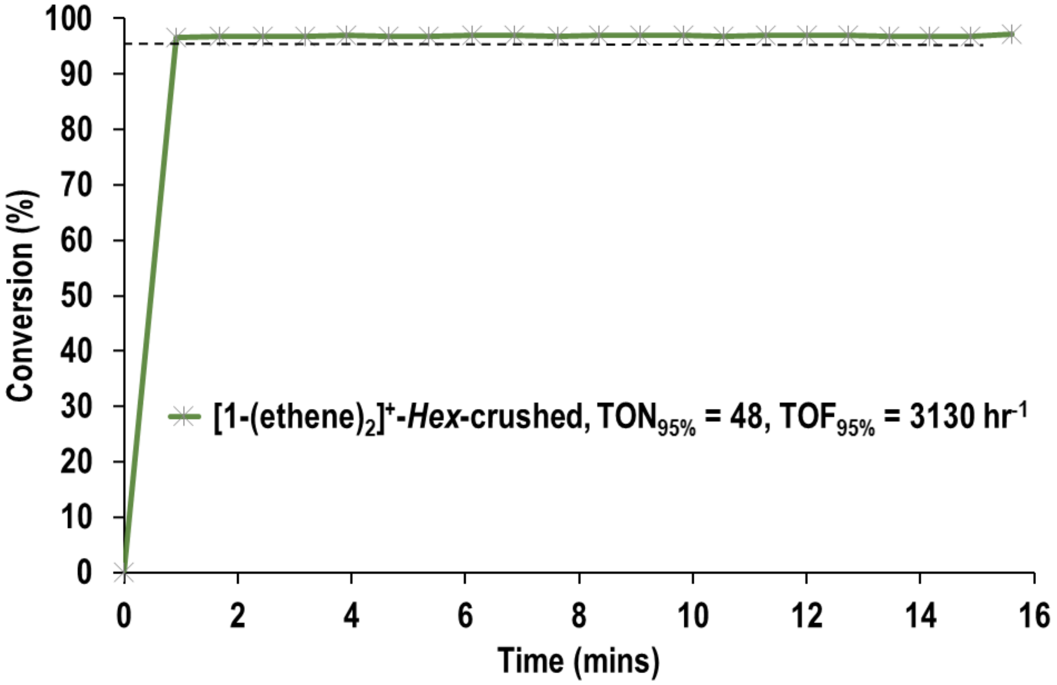

Figure S50: Data for the catalytic isomerization of 1-butene to 2-butene using **[1-(ethene)$_2$][BAr$^F{}_4$]-*Hex*-crushed** as measured by gas phase ^{1}H NMR spectroscopy. The dashed line indicates 95% conversion. Thermodynamic equilibrium conditions for the isomerization (1-butene:2-butene. 97:3%) are reached within 1 min of catalysis.

S.4.2. Catalyst recyclability for the gas phase isomerization of 1-butene to 2-butene

S.4.2.1. General experimental considerations for the catalyst recycling studies

Recycling studies were carried out by charging a J Young high pressure NMR tube with a given catalyst (2.5 mg each) and allowing to react with 1-butene gas as accounted in S.4.1.1. Once catalysis reached the thermodynamic equilibrium conditions for the isomerization (mixture of 1-butene:2-butene of 4:96, approximately) the timer was stopped, the NMR tube removed from the NMR diffractometer. Then it was sequentially, fitted back to the Schlenk line using the T-piece adaptor (see Figure S2), evacuated (< 1 x 10^{-3} mbar) under dynamic vacuum for 30 sec, and then the NMR tube was charged with fresh 1-butene gas (15 psi) and sealed with a Teflon stopcock as a timer was started. Throughout each recycling experiment the 1-butene gas regulator was left untouched to ensure the same pressure upon refilling the NMR tube.

S.4.2.1.1. Catalyst recycling studies for [1-NBA][BAr$^F{}_4$]-large

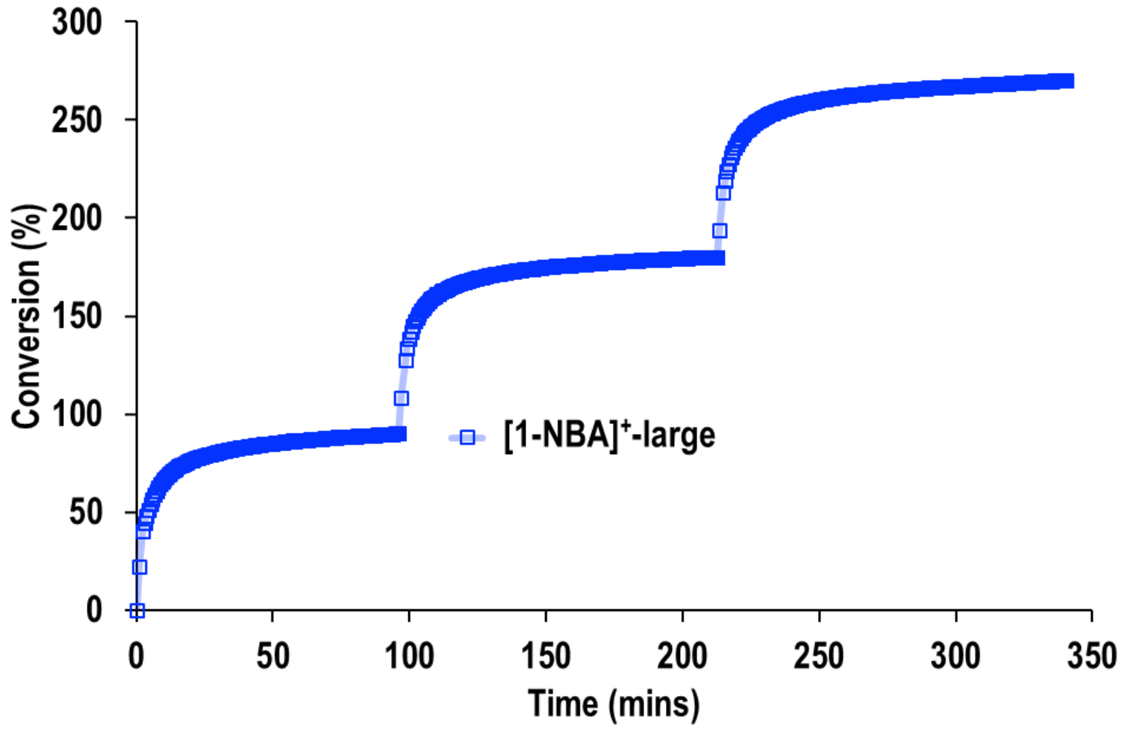

Figure S51: Data for the catalytic recyclability isomerization of 1-butene to 2-butene using **[1-NBA][BAr$^F{}_4$]-large** as measured by gas phase ^{1}H NMR spectroscopy. This dataset correspond to recyclability experiments over three 1-butene recharges after 90% conversion was achieved for each catalytic run.

S.4.2.1.2. Catalyst recycling studies for [1-(ethene)$_2$][BAr$^F_4$]-*Oct*-large

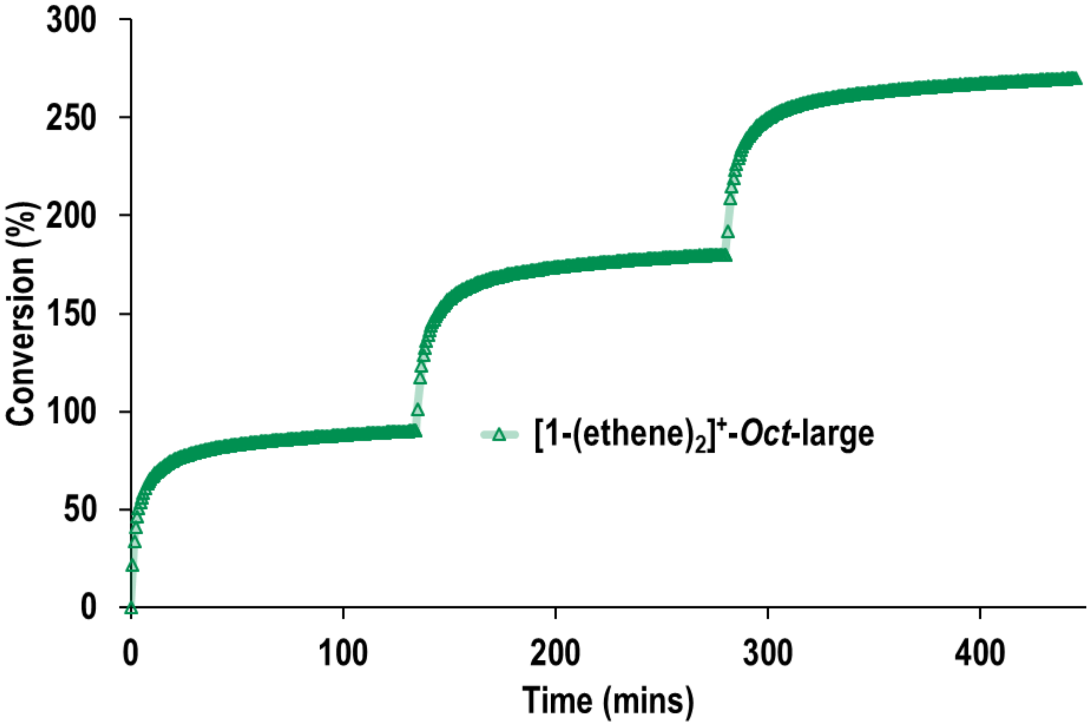

Figure S52: Data for the catalytic recyclability isomerization of 1-butene to 2-butene using **[1-(ethene)$_2$][BAr$^F_4$]-*Oct*-large** as measured by gas phase ^{1}H NMR spectroscopy. This dataset correspond to recyclability experiments over three 1-butene recharges after 90% conversion was achieved for each catalytic run.

S.4.2.1.3. Catalyst recycling studies for [1-(ethene)$_2$][BAr$^F{}_4$]-*Hex*-large

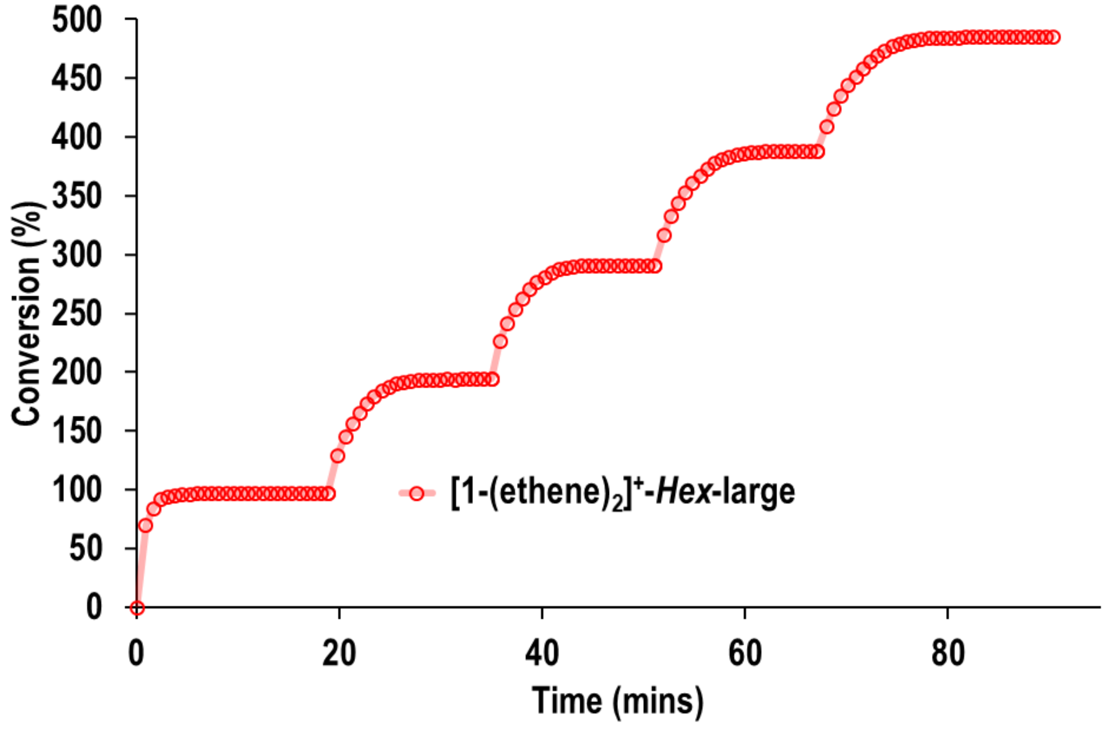

Figure S53: Data for the catalytic recyclability isomerization of 1-butene to 2-butene using **[1-(ethene)$_2$][BAr$^F{}_4$]-*Hex*-large** as measured by gas phase ^{1}H NMR spectroscopy. This dataset correspond to recyclability experiments over five 1-butene recharges after thermodynamic equilibrium conditions have been reached (1-butene:2-butene, 3:97% conversion) for each catalytic run.

S.4.2.1.4. Catalyst recycling studies for [1-NBA][BAr$^F_4$]-crushed

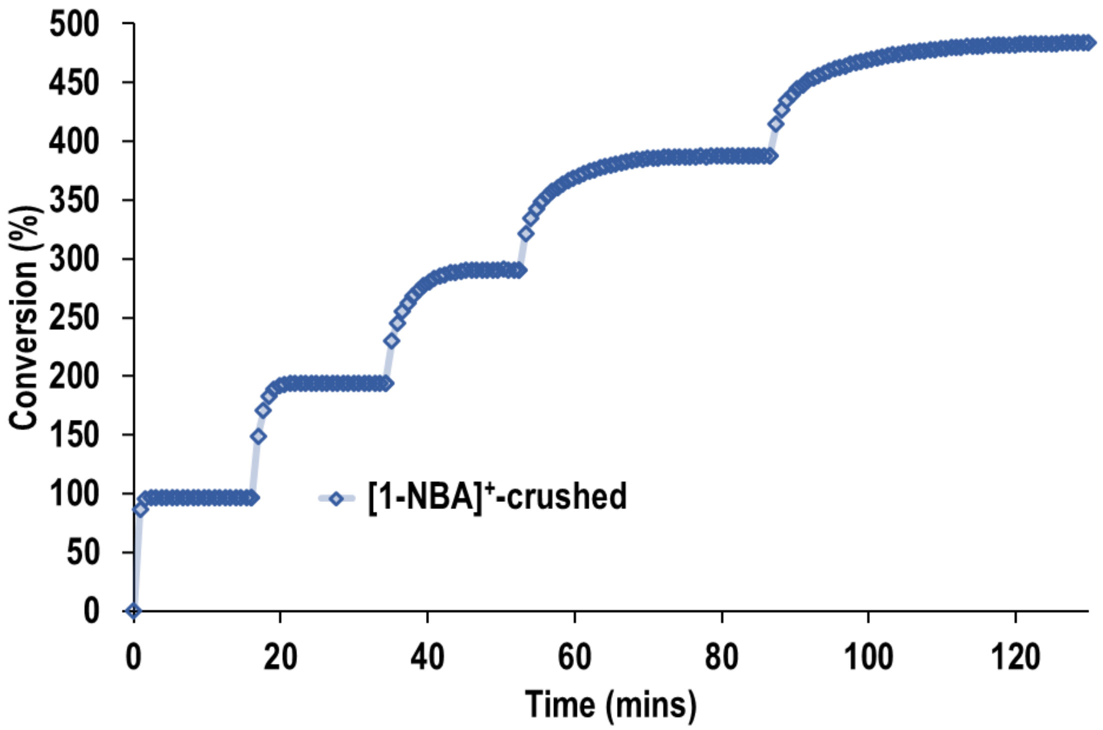

Figure S54: Data for the catalytic recyclability isomerization of 1-butene to 2-butene using **[1-NBA][BAr$^F_4$]-crushed** as measured by gas phase ^{1}H NMR spectroscopy. This dataset correspond to recyclability experiments over five 1-butene recharges after thermodynamic equilibrium conditions have been reached (1-butene:2-butene, 3:97% conversion) for each catalytic run.

S.4.2.1.5. Catalyst recycling studies for [1-(ethene)$_2$][BAr^F$_4$]-*Oct*-crushed

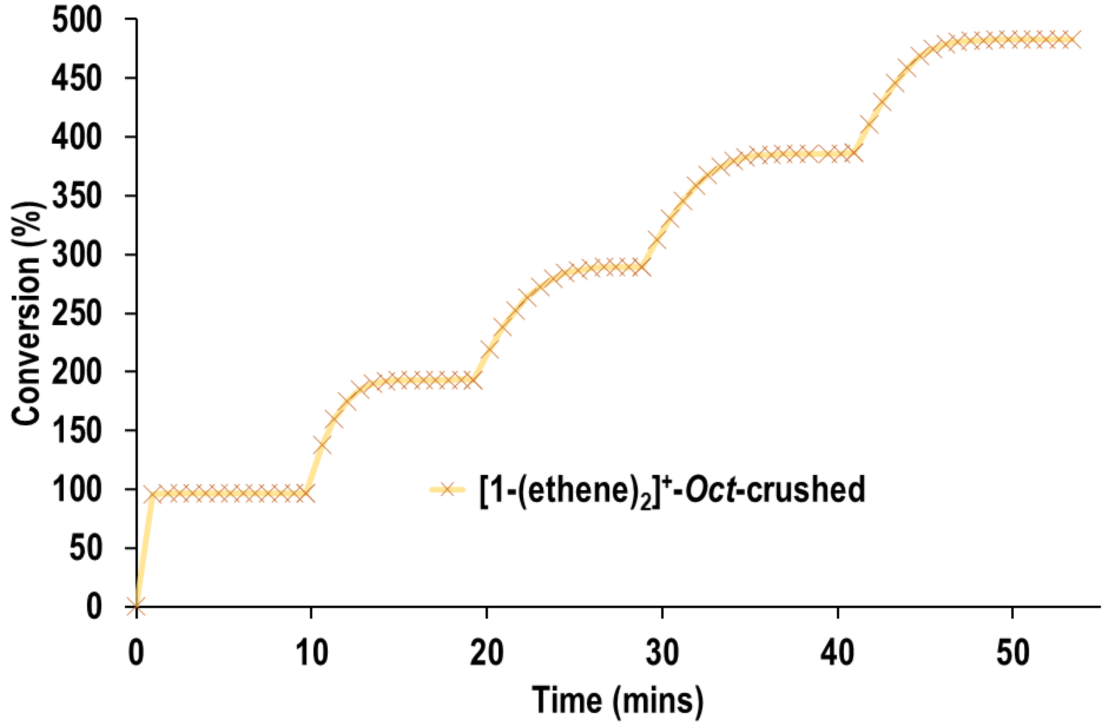

Figure S55: Data for the catalytic recyclability isomerization of 1-butene to 2-butene using **[1-(ethene)$_2$][BAr^F$_4$]-*Oct*-crushed** as measured by gas phase ^{1}H NMR spectroscopy. This dataset correspond to recyclability experiments over five 1-butene recharges after thermodynamic equilibrium conditions have been reached (1-butene:2-butene, 3:97% conversion) for each catalytic run.

S.4.2.1.6. Catalyst recycling studies for [1-(ethene)$_2$][BAr$^F{}_4$]-*Hex*-crushed

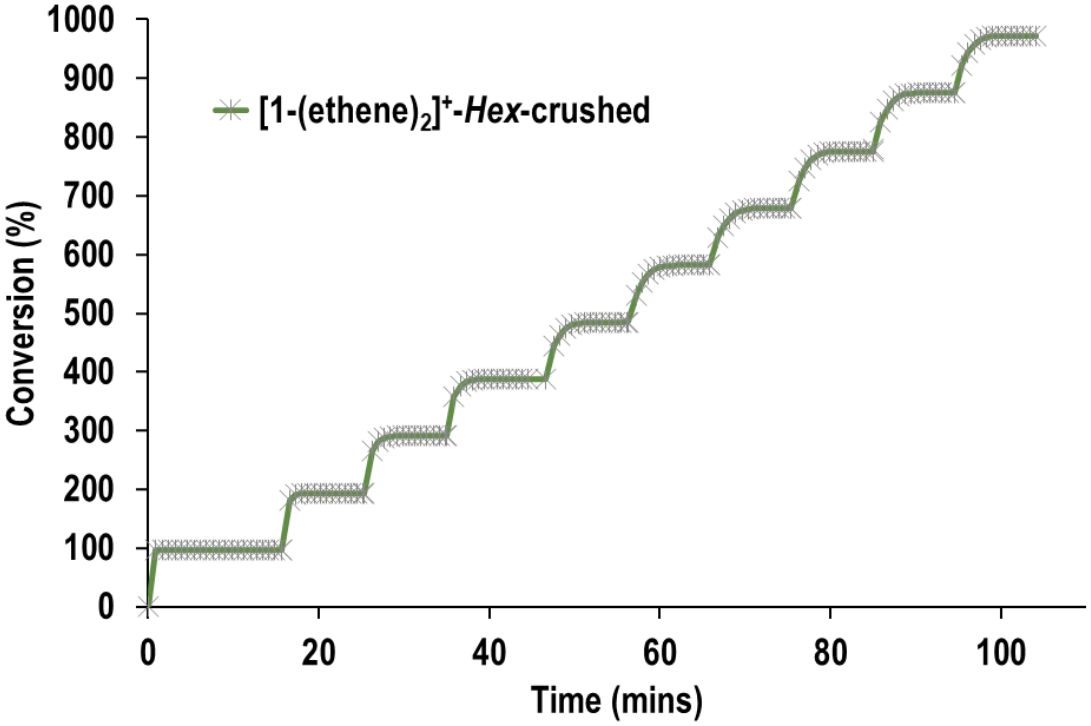

Figure S56: Data for the recyclability for catalytic isomerization of 1-butene to 2-butene using **[1-(ethene)$_2$][BAr$^F{}_4$]-*Hex*-crushed** as measured by gas phase ^{1}H NMR spectroscopy. This dataset correspond to recyclability experiments over ten 1-butene recharges after thermodynamic equilibrium conditions have been reached (1-butene:2-butene, 3:97% conversion) for each catalytic run.

S.4.2.1.7. Comparison of TON and TOF for the various SMOM-catalysts

Table S3: Catalytic data for the recyclability in the isomerization of 1-butene to 2-butene using the catalysts **[1-NBA][BAr$^F{}_4$]-large**, **[1-(ethene)$_2$][BAr$^F{}_4$]-*Oct*-large** and **[1-(ethene)$_2$][BAr$^F{}_4$]-*Hex*-large**. These datasets are for the recyclability experiments over three 1-butene recharges for **[1-NBA][BAr$^F{}_4$]-large** and **[1-(ethene)$_2$][BAr$^F{}_4$]-*Oct*-large**, whilst five 1-butene recharges for **[1-(ethene)$_2$][BAr$^F{}_4$]-*Hex*-large**.

catalyst	catalytic run	$TON_{90\%}$	$TOF_{90\%}$
**[1-NBA]-large**	1st	46	29
	2nd	46	24
	3rd	46	22
**[1-(ethene)$_2$]-*Oct*-large**	1st	44	20
	2nd	44	19
	3rd	44	17
**[1-(ethene)$_2$]-*Hex*-large**	1st	45	1160
	2nd	45	450
	3rd	45	450
	4th	45	400
	5th	45	350

Table S4: Catalytic data for the recyclability in the isomerization of 1-butene to 2-butene using the catalysts **[1-NBA][BAr^F$_4$]-crushed**, **[1-(ethene)$_2$][BAr^F$_4$]-*Oct*-crushed** and **[1-(ethene)$_2$][BAr^F$_4$]-*Hex*-crushed**. These datasets are for the recyclability experiments over five 1-butene recharges for **[1-NBA][BAr^F$_4$]-crushed** and **[1-(ethene)$_2$][BAr^F$_4$]-*Oct*-crushed** whilst ten 1-butene recharges for **[1-(ethene)$_2$][BAr^F$_4$]-*Hex*-crushed**.

catalyst	catalytic run	$TON_{90\%}$	$TOF_{90\%}$
**[1-NBA]-large**	1st	79	29
	2nd	49	24
	3rd	48	24
	4rd	48	24
	5th	48	22
**[1-(ethene)$_2$]-*Oct*-large**	1st	47	20
	2nd	47	19
	3rd	47	19
	4rd	47	19
	5th	47	17
**[1-(ethene)$_2$]-*Hex*-large**	1st	48	3130
	2nd	47	1700
	3rd	47	1190
	4rd	47	1040
	5th	47	680
	6th	47	680
	7th	47	580
	8th	47	680
	9th	47	680
	10th	47	680

S.4.2.2. Surface *versus* bulk catalytic studies

S.4.2.2.1. Surface passivation studies

Samples of **[1-NBA][BAr$^F_4$]-large** and **[1-NBA][BAr$^F_4$]-crushed** were exposed to CO (15 and 30 psi for 10 and 150 seconds, respectively) to give the expected complex [($Cy_2PCH_2CH_2PCy_2$)Rh$(CO)_2$][BAr$^F_4$]. Solution $^{31}P\{^1H\}$ NMR (in $F_2C_6H_4$) spectroscopy showed formation of [($Cy_2PCH_2CH_2PCy_2$)Rh$(CO)_2$][BAr$^F_4$] (ca. 25 and 70% conversion, exposing to/for 15/10 and 30/150 psi/seconds of CO, respectively) whilst the remaining unreactive **[1-NBA][BAr$^F_4$]** was converted to **[1-$F_2C_6H_4$][BAr$^F_4$]** upon dissolution in 1,2-$F_2C_6H_4$. [($Cy_2PCH_2CH_2PCy_2$)Rh$(CO)_2$][BAr$^F_4$] is inactive towards the catalytic isomerization of 1-butene to 2-butene. Assuming that the surface of the crystals (both large and crushed) would react faster than the bulk, effectively turning off the surface for catalysis – the intention being investigating whether this is a surface process or bulk process. However with **[1-NBA][BAr$^F_4$]-large** significant loss of crystallinity (cracking of the passivated surface) of the crystals occurred upon reaction with CO as observed by optical microscopy (see Figure S57), which likely exposed the interior of the passivated crystals for the catalytic isomerization of 1-butene to 2-butene . Presumably the same would be happening on **[1-NBA][BAr$^F_4$]-crushed**, but not be observable by optical microscopy.

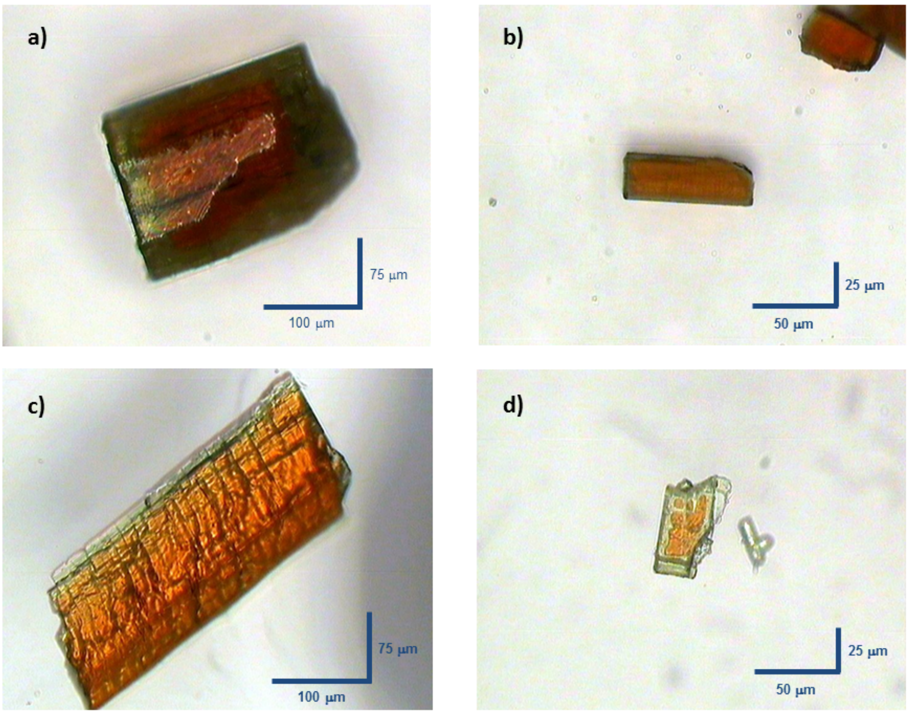

Figure S57: Optical microscope pictures of a) big and b) small size single crystals of **[1-NBA][BAr$^F_4$]-large**, and c) big and d) small size **[1-NBA][BAr$^F_4$]-large** crystals exposed to CO (15 psi, 298 K) for 10 seconds showing extensive cracking of the CO-passivated-surface of the crystals.

S.4.2.2.2. Solid state NMR study

A sample of **[1-NBA][BAr$^{F}_{4}$]-large** (c. 70 mg) in a J. Young's flask (c. 50 mL) was treated with 1-butene (15 psi, 298 K). After 5 minutes at room temperature the flask was then evacuated (1 × 10^{-2} mbar). The sample was then loaded into a 4 mm rotor and capped off in an argon filled glovebox. A $^{31}P\{^{1}H\}$ NMR spectrum revealed the sample still contained a significant quantity of **[1-NBA][BAr$^{F}_{4}$]** (Figure S58).

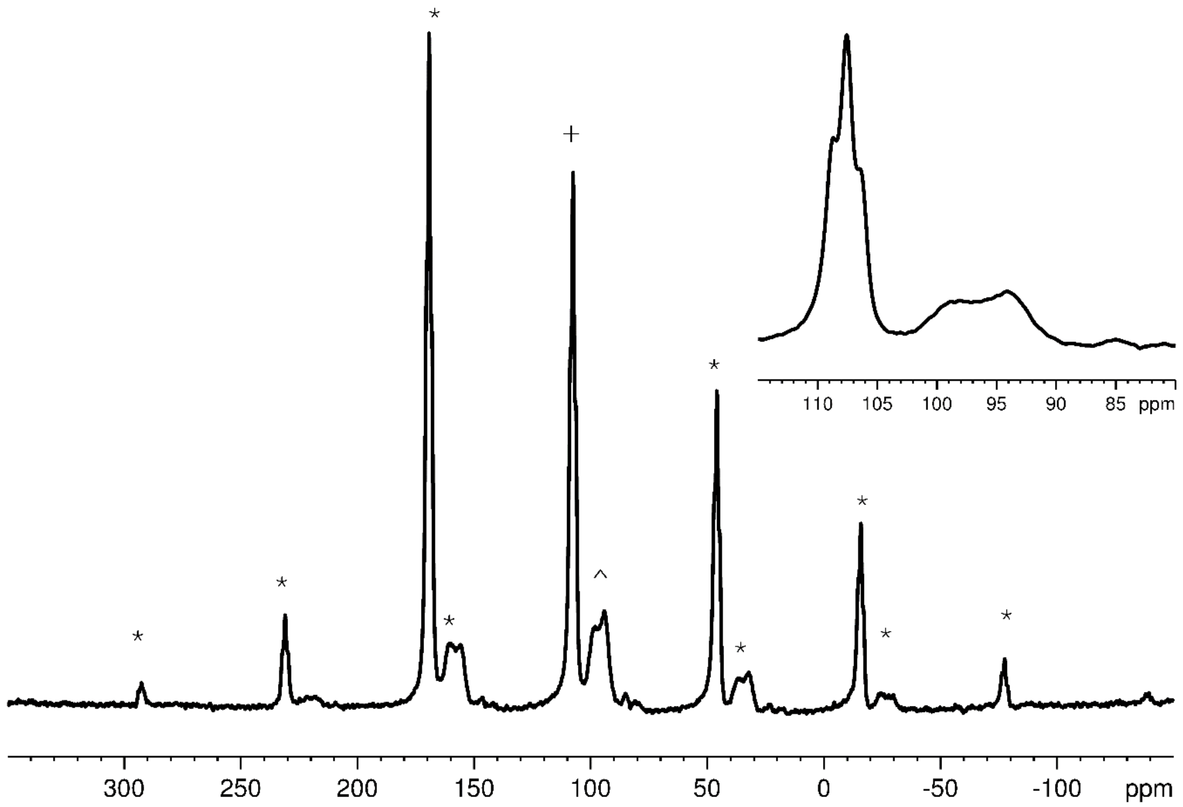

Figure S58: The $^{31}P\{^{1}H\}$ SSNMR spectrum (162 MHz, 298 K, 10 kHz spin rate) of **[1-NBA][BAr$^{F}_{4}$]**-large treated with 1-butene (15 psi) for 5 minutes. The resonance marked + is due to **[1-NBA][BAr$^{F}_{4}$].** The resonance marked ^ is due to **[1-(butene)][BAr$^{F}_{4}$].** The resonances marked * are due to spinning sidebands. The inset is a zoom of the central resonances.

S.4.3. Catalytic transfer dehydrogenation of butane to 2-butene

S.4.3.1. Experimental details for the catalytic transfer dehydrogenation

Encouraged by the reaction of **[1-NBA][BAr$^{F}_{4}$]** with 1-butene to form **[1-(butadiene)][BAr$^{F}_{4}$]**, investigation into the ability of **[1-NBA][BAr$^{F}_{4}$]** to act as a transfer dehydrogenation catalyst was undertaken (Scheme S3). A typical experiment was as follows – a high pressure NMR tube (sealed with a Teflon stopcock) was loaded with 6 mg (0.00405 mmol) of **[1-NBA][BAr$^{F}_{4}$]** in an argon-filled glove box. This was then taken out of the glove box, and evacuated on a Schlenk

line (< 1 × 10^{-2} mbar). To this butane gas was added and the stopcock sealed. The gas feed was changed to ethene and set to the appropriate pressure so that the total pressure was 15 psi (Table S5). The glass T-piece and connecting tubing was evacuated and refilled three times (with ethene), before the stopcock was opened. The loaded tubes were left to stand, and the reaction monitored by gas phase ^{1}H NMR of the head space, and though conditions have not been optimized, transfer dehydrogenation definitively occurs. No other hydrogen acceptors were examined.

Scheme S3: The transfer dehydrogenation reaction, catalyst loadings were 6 mg, and catalyst used were **[1-NBA][BAr$^{F}_{4}$]** and **[1-(ethene)$_2$][BAr$^{F}_{4}$]-*Hex***.

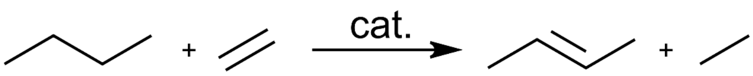

Table S5: Data table for transfer dehydrogenation studies.

Catalyst	% Conv. butane to butene	Time (hrs)	Temp. (°C)	starting ethene:butane ratio	min. TON
**[1-NBA]$^{+}$-crushed**	63%	168	80	2:1	3.86
**[1-NBA]$^{+}$-crushed**	40%	24	80	2:1	2.45
**[1-NBA]$^{+}$-crushed**	15%	72	25	1:1	1.39
**[1-NBA]$^{+}$-crushed**	27%	24	25	1:2	3.31
**[1-NBA]$^{+}$-crushed**	33%	168	25	1:2	4.04
**[1-NBA]$^{+}$-crushed**	18%	24	25	1:2	2.21
**[1-(ethene)$_2$]$^{+}$-*Hex***	18%	24	25	1:2	2.05

Acceptorless dehydrogenation (i.e. in the absence of ethene) showed no evidence of proceeding by gas phase ^{1}H NMR spectroscopy; however, the **[1-NBA][BAr$^{F}_{4}$]** catalyst was converted to **[1-(butadiene)][BAr$^{F}_{4}$]**. This was shown to be due to traces amounts of butene impurity within the gas – a sample of **[1-NBA][BAr$^{F}_{4}$]** (10 mg) was put under butane gas and left to stand overnight, forming **[1-(butadiene)][BAr$^{F}_{4}$]** (approximately quantitatively by ^{31}P{^{1}H} solution NMR spectroscopy). The headspace gas was then transferred (by vacuum transfer) onto a new sample of **[1-NBA][BAr$^{F}_{4}$]**, and some **[1-(butadiene)][BAr$^{F}_{4}$]** was formed, though not quantitatively (by ^{31}P{^{1}H} solution NMR spectroscopy). Transfer of the gas onto a third sample showed no evidence of **[1-(butadiene)][BAr$^{F}_{4}$]** formation.

S.4.4. Oligomerization of ethene

[1-NBA][BAr$^{F}_{4}$] (10 mg, 0.00673 mmol, crushed) was loaded into a high pressure NMR tube in an argon-filled glove box. This was then evacuated on a Schlenk line (< 1 × 10^{-2} mbar) before being filled with ethene gas (15 psi, 298 K). This was left to stand for one week at ambient temperature. After one week, gas phase ^{1}H NMR

spectroscopy showed the formation of approximately one equivalent 2-butene gas (Figure S59).

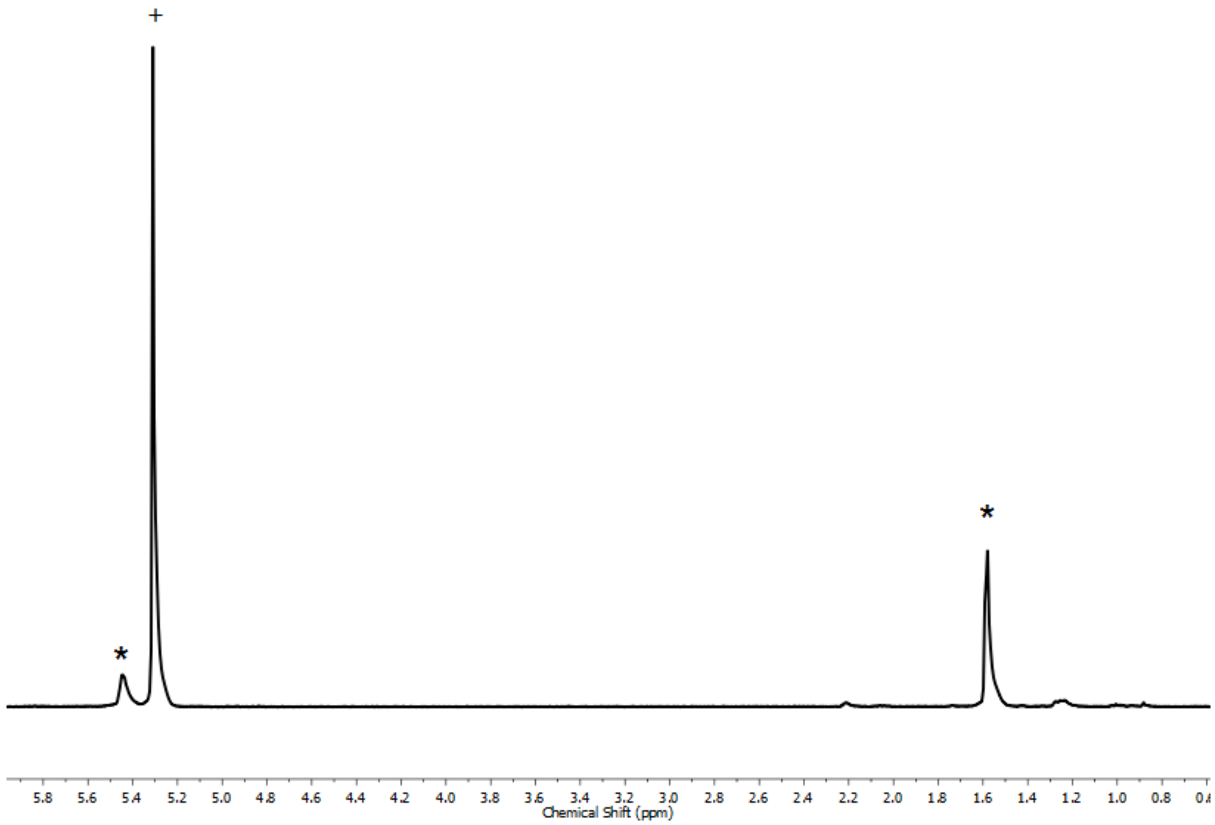

Figure S59: The gas phase ^{1}H NMR spectrum (298 K, 400 MHz) of a sample of **[1-NBA][BAr^F$_4$]-crushed** which was left under ethene (15 psi, 298 K) for one week. The resonance marked + is due to ethene, the resonances marked * are 2-butene.

S.5. Computational studies

S.5.1. Computational methods

Geometry optimizations. All static Kohn-Sham DFT calculations were performed on periodic models of the studied rhodium complexes, employing the Gaussian Plane Wave (GPW) formalism as implemented in the QUICKSTEP[S18] module within the CP2K program suite (Version 5.0).[S19] Molecularly optimized basis sets of double-ζ quality plus polarization in their short-range variant (DZVP-MOLOPT-SR-GTH)[S20] were used on all atomic species. The interaction between the core electrons and the valence shell (Rh: 17, B: 3, C: 4, P: 5, F: 7, H: 1 electrons) was described by Goedecker-Teter-Hutter (GTH) pseudo potentials.[S21-23] The generalized gradient approximation (GGA) to the exchange-correlation functional according to Perdew-Burke-Ernzerhof (PBE)[S24] was used in combination with Grimme's D3-correction for dispersion interactions.[S25] The auxiliary plane wave basis set was truncated at a cutoff of 500 Ry. The maximum force convergence criterion was set to 10^{-4} E_h·$Bohr^{-1}$, whilst default values were used for the remaining criteria. The convergence criterion for the self-consistent field (SCF) accuracy was set to 10^{-7} E_h and 10^{-8} E_h for geometry optimizations and vibrational analysis, respectively. Transition states were located utilizing the dimer method[S26] starting from an initial guess structure, and applying the same convergence criteria as defined above for a regular optimisation. In some cases the initial structure was found by applying the climbing image nudged elastic band (CI-NEB)[S27] approach with 5 images. Here the maximum force criterion was loosened to $8{\cdot}10^{-4}$ E_h·$Bohr^{-1}$ in order to speed up the optimization. Likewise, the SCF accuracy was reduced to 10^{-6} E_h to overcome SCF convergence problems in some cases. Optimized stationary points were characterized by analysis of their numerical second derivatives with a displacement of 0.01 Bohr. For local minima the Hessian matrix contains only positive eigenmodes, whilst transition states have exactly one imaginary eigenvalue. Thermal corrections (T = 298.15 K, p = 1 bar) were calculated based on a partial Hessian of the cationic portion in each structure within the harmonic oscillator approximation (HO). The TAMkin[S28] software tool in conjunction with the partial Hessian vibrational analysis (PHVA) scheme was utilized to diagonalize the Hessian matrix and generate all partition functions. The Brillouin zone was sampled using the Γ-point. Initial coordinates were obtained from the experimental crystal structure, from which other structures were manually generated. Periodic boundary conditions (PBC) were applied throughout in combination with fixed unit cell parameters obtained from experiment (**[1–(propene)][BAr$^F_4$]**: *a* 12.7894 Å *b* 12.8961 Å *c* 19.8711 Å α 91.2560° β 90.7450° γ 96.8630°; **[1–(butene)][BAr$^F_4$]**: *a* 19.1979 Å *b* 17.1549 Å *c* 19.9732 Å α 90.0000° β 90.3100° γ 90.000°). All geometries were fully relaxed without imposing constraints, whilst keeping unit cell parameters constant. The geometries of the isolated cationic portion of **[1–(propene)][BAr$^F_4$]** (**I$^+$**) and free propene were fully optimized following the same protocol as outlined above, but here a non-periodic cubic supercell of length

25 Å was employed. For comparison with solution phase reactivity, geometries of **I^+**, its *bis*-η^2-propene adduct (**I_{bis}^+**) and molecular propene were fully optimised using Gaussian 09 (Revision D.01),[S29] without imposing symmetry constraints (C_1 symmetry). Initial coordinates for **I_{bis}^+** revealing the relative orientation of the two propene units were extracted from the experimental single crystal X-ray structure of **[1–(propene)$_2$][BAr$^F{}_4$]**. These calculations employed the SMD(CH_2Cl_2)/BP86/SDD(Rh,P)/6-31G(d,p) level of theory, with a polarization functions added for P (ζ = 0.387). Details of this type of calculation have been reported elsewhere.[S1] Effects on the geometry due to solute-solvent interactions were treated implicitly with a polarizable continuum model, using the IEFPCM formalism in conjunction with the SMD[S30] model, where the chosen dielectric constant (ε = 8.93) corresponds to that of dichloromethane. The D3-correction for dispersion interactions was applied following geometry optimisation.

Calculation of solid-state and in vacuo nuclear magnetic resonance parameters. Isotropic ^{13}C and ^{1}H magnetic shielding constants (σ_{iso}) were generated using the GIPAW method[S31,S32] as implemented in CASTEP 8.0.[S33] Single-point calculations were performed on the fully optimized crystal structures. The plane wave basis was truncated at 80 Ry, while the Brillouin zone was sampled using a Monkhorst-Pack grid[S34] with *k*-point spacing of 0.04 Å^{-1}. These settings were shown to yield converged NMR parameters on related systems.[S6,35] The calculations were performed using the PBE GGA functional.[S24] Ultra-soft pseudopotentials were generated on-the-fly to represent the core electron shells. Scalar-relativistic effects were incorporated through the zeroth-order regular approximation (ZORA).[S36-37] The default ultrafine SCF convergence threshold was used in the calculations (10^{-8} eV atom^{-1}). Computed ^{13}C isotropic shielding constants were converted into chemical shift values (δ) via a linear regression procedure.[S38] To this end, calculated isotropic magnetic shielding tensors were plotted against selected experimental chemical shifts to yield a linear correlation plot (see Supporting Figure S62). Data were collected for one asymmetric unit within the unit cell and averaged for the carbon atoms in the four $C_6H_3(CF_3)_2$ residues present in the BAr$^F{}_4$ counterion. Chemical shifts were then determined from the expression $\delta(^{13}C) = (\text{intercept} - \sigma_{iso}) / m$, where the intercept with the y-axis corresponds to σ_{ref} of the reference compound and *m* is the slope. The absence of sufficiently large set of data for proton chemical shifts prevents the use of the linear regression method for this nucleus. Hence, relative isotropic proton (^{1}H) chemical shifts were obtained by referencing computed shielding constants against those of the standard tetramethylsilane (TMS) according to $\delta_{iso}(^1H) = \sigma_{ref}(TMS) - \sigma_{iso}$, where σ_{ref}(TMS) was calculated as 31.15 ppm (cubic supercell with length 20 Å).[S6,35] ^{1}H chemical shifts of cationic species **[1–(propene)]$^+$** and **[1–(allyl hydride)]$^+$** were calculated based on their CP2K-optimized gas phase

geometries. As in the case for TMS, a cubic supercell with length 20 Å was utilized in the GIPAW calculations.

S.5.2. Optimized geometries

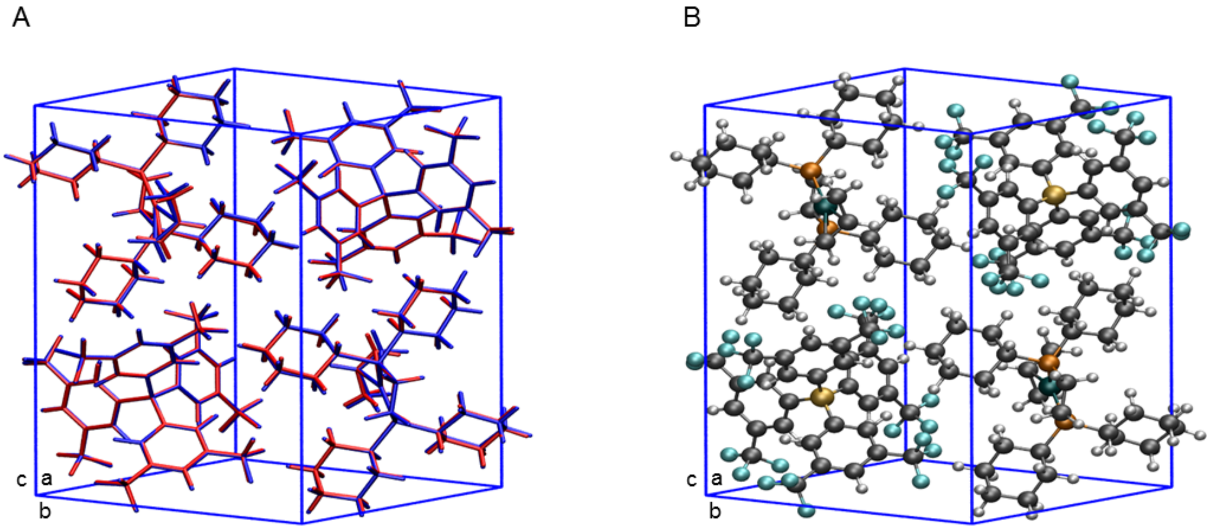

Figure S60: (**A**) Overlay of the crystal structure (red) and the DFT-optimized structure (blue) of **[1–(propene)][BAr^F$_4$]**. (**B**) DFT-optimized geometry of **[1–(propene)][BAr^F$_4$]** (C grey, P orange, F blue, B yellow, Rh green, H white).

Table S6: Key optimized bond metrics (Å, °) for **[1–(propene)][BAr^F$_4$]**.

Parameter	PBE-D3	Parameter	PBE-D3
Rh–P1	2.287	P1–Rh–P2	84.89
Rh–P2	2.227	C100–C200–C300	120.55
Rh–C100	2.424	P1–Rh–C100	117.56
Rh–C200	2.143	P1–Rh–C200	152.47
Rh–C300	2.260	P1–Rh–C300	164.55
C100–C200	1.496	P2–Rh–C100	156.26
C200–C300	1.392	P2–Rh–C200	118.69
Rh···H100	1.939	P2–Rh–C300	95.93
C100–H100	1.167	P1–Rh–H100	89.28

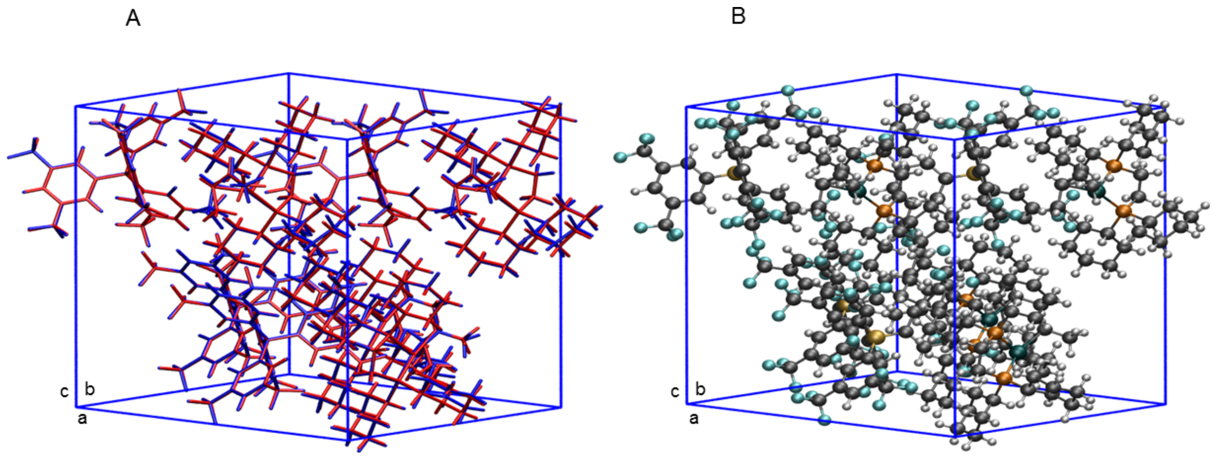

Figure S61: (**A**) Overlay of the crystal structure (red) and the DFT-optimized structure (blue) of **[1–(butene)][BAr^F$_4$]**. (**B**) DFT-optimized geometry of **[1–(butene)][BAr^F$_4$]** (C grey, P orange, F blue, B yellow, Rh green, H white).

Table S7: Key optimized bond metrics (Å, °) for **[1–(butene)][BAr^F$_4$]**.

**Parameter**	**PBE-D3**	**Parameter**	**PBE-D3**
Rh–P1	2.285	P1–Rh–P1’	84.92
Rh–P1’	2.214	C1–C2–C3	121.08
Rh–C1	2.506	C2–C3–C4	124.36
Rh–C2	2.145	P1–Rh–C1	119.82
Rh–C3	2.260	P1–Rh–C2	155.39
Rh–C4	2.989	P1–Rh–C3	158.55
C1–C2	1.501	P1–Rh–C4	129.70
C2–C3	1.398	P1’–Rh–C1	148.11
C3–C4	1.507	P1’–Rh–C2	114.74
Rh···H1	2.045	P1’–Rh–C3	101.11
C1–H1	1.150	P1’–Rh–C4	114.72

S.5.3. Calculated $^{1}H/^{13}C$ NMR chemical shifts

The GIPAW calculations utilized the fully optimized structure of **[1–(propene)][BAr$^F_4$]** and **[1–(butene)][BAr$^F_4$]**. Linear regression plots for deriving parameters σ_{ref} and slope m for both compounds are shown in Figure S62. In each plot one outlier can be identified, which corresponds to carbon in the CF_3 groups. A comparison of the fully optimized and crystal structures reveals that C–F bond distances are overestimated by ~0.06 Å in the fully optimized geometry, which in turn results in a decrease of the (averaged) magnetic shielding constant by ~15 ppm relative to the one obtained for the partially relaxed structure. This outlier was therefore omitted in the linear fits. As a result of the notably reduced σ_{iso}, the associated chemical shift $\delta(^{13}C)$ for the CF_3 group is too large.

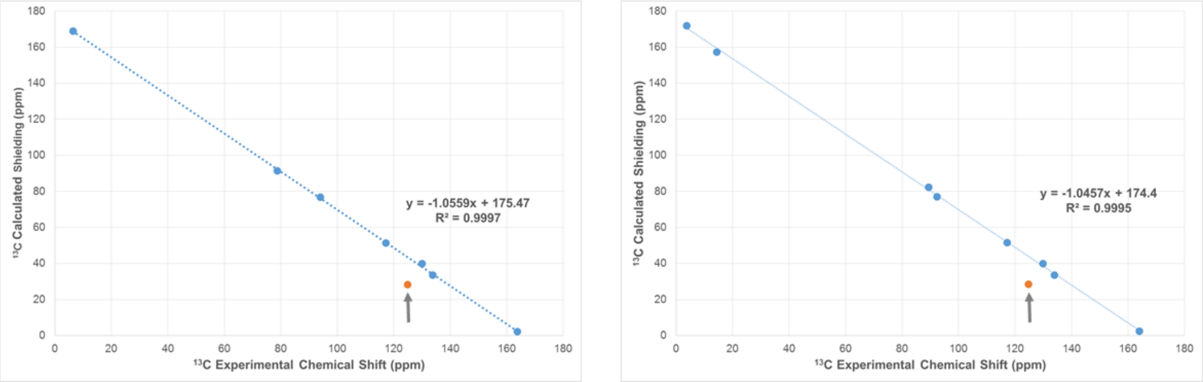

Figure S62: Correlation between calculated isotropic ^{13}C magnetic shielding tensors and experimental chemical shifts. Left: **[1–(propene)][BAr$^F_4$]**, right: **[1–(butene)][BAr$^F_4$]** The arrow indicates an outlier in the fit, corresponding to the $\mathit{C}F_3$ resonance. This data point results from an overestimation of the C-F bond lengths, and was not included in the linear regression.

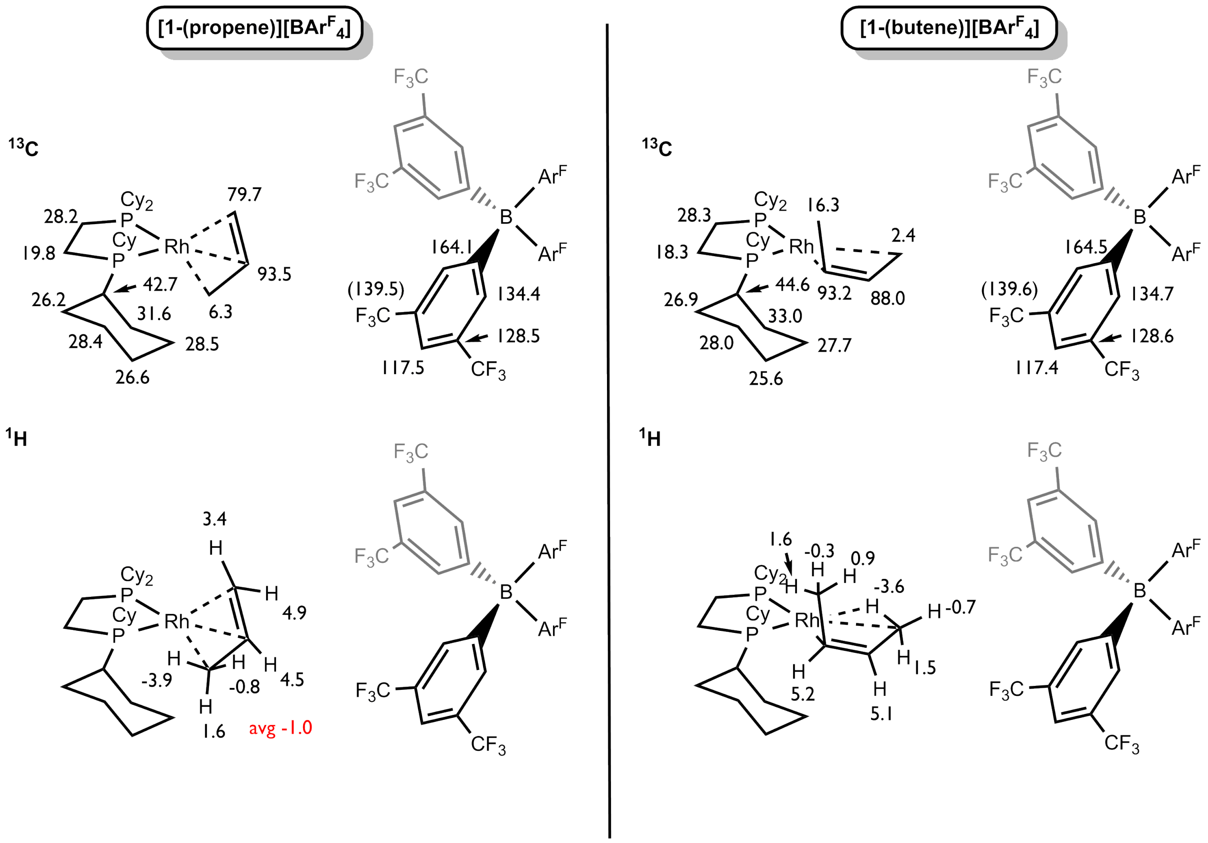

Figure S63: Summary of GIPAW NMR results (PBE/cutoff 80Ry) for bulk **[1–(propene)][BAr^F$_4$]** and **[1–(butene)][BAr^F$_4$]**. (Reference: δ(^{13}C) linear regression, δ(^{1}H) TMS; chemical shifts are given in ppm).

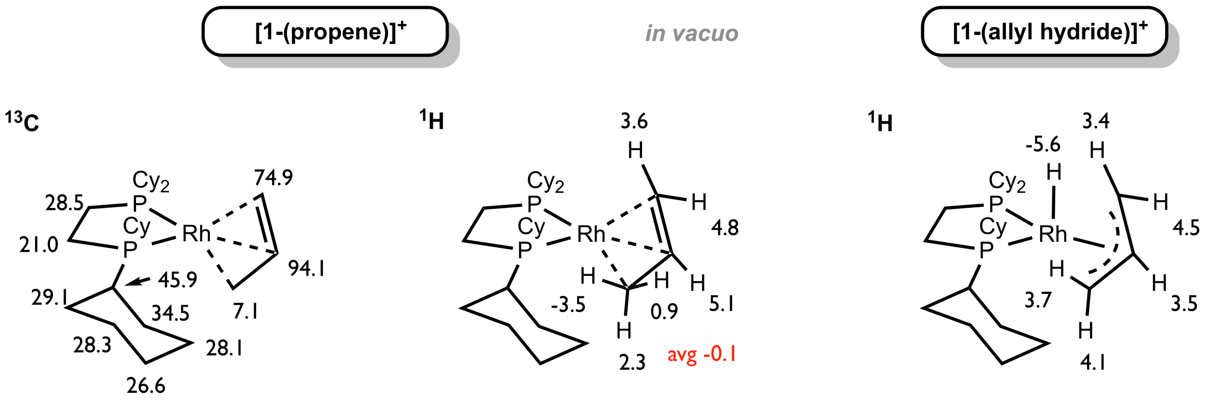

Figure S64: Summary of GIPAW NMR results (PBE/cutoff 80Ry) for the isolated cationic portions **[1–(propene)]$^+$** and **[1–(allyl hydride)]$^+$**. (References: δ(^{13}C) linear regression, δ(^{1}H) TMS, chemical shifts are given in ppm).

(a)

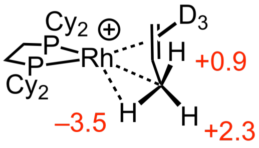

A

$\delta(^1H)$: 0.33·(–3.5) + 0.33·(+2.3) + 0.33·(+0.9) = **–0.10**

(b)

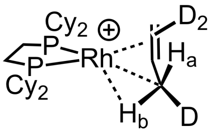

B

$\delta(^1H_a)$: 0.246·(+2.3) + 0.376·(–3.5) + 0.378·(+0.9) = **–0.41**

$\delta(^1H_b)$: 0.246·(+0.9) + 0.376·(+2.3) + 0.378·(–3.5) = **–0.24**

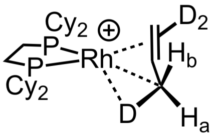

24.6%

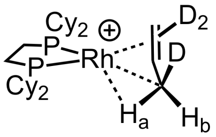

37.6%

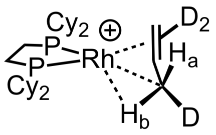

37.8%

(c)

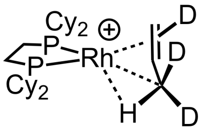

C

$\delta(^1H)$: 0.434·(–3.5) + 0.284·(+0.9) + 0.282·(+2.3) = **–0.62**

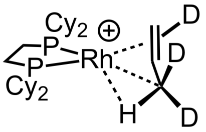

43.4%

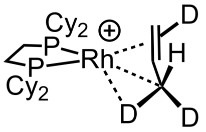

28.4%

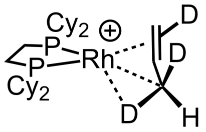

28.2%

Figure S65: Summary of GIPAW NMR results (PBE/cutoff 80Ry) for the isolated cationic species **[1–(d$_3$-propene)]$^+$** and its isotopomers. Boltzmann weighting factors ($p_i = e^{-\Delta Gi/kT} / \sum_j e^{-\Delta Gj/kT}$) are based on relative Gibbs free energies for each isotopomer/rotamer. Gibbs energies for each isotopomer were calculated by permuting positions of the deuterium label and generating the corresponding partition function. ($\delta(^1H)$ relative to TMS, chemical shifts are given in ppm).

S.5.4. Mechanisms

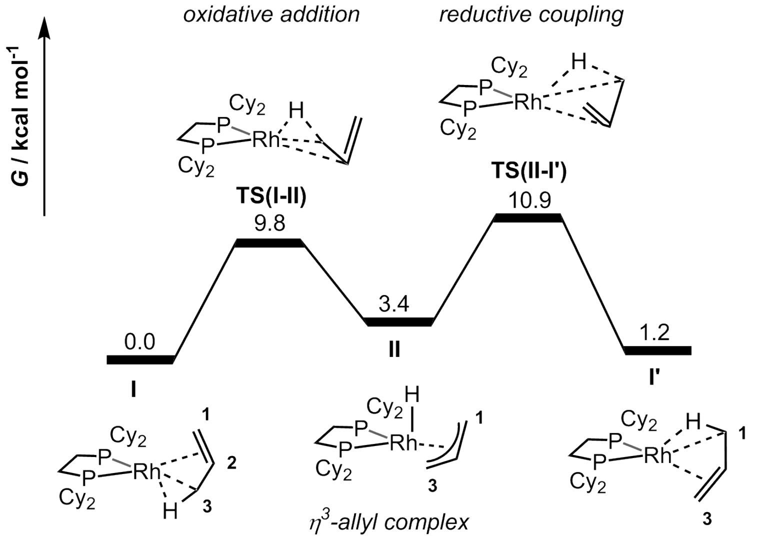

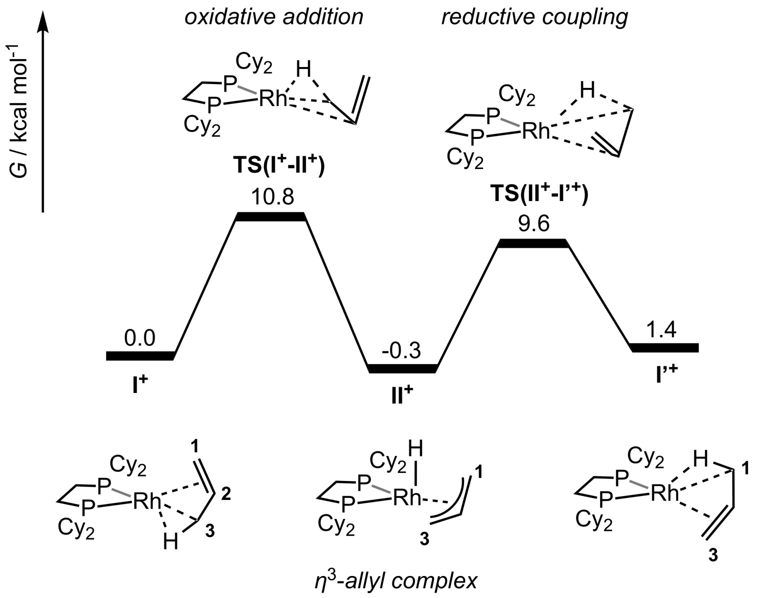

Figure S66: Top: calculated Gibbs Free Energy profile (periodic system) for the 1,3-H shift reaction via oxidative cleavage / reductive coupling in **[1–(propene)][BAr$^{F}{}_{4}$]**. Bottom: calculated Gibbs Free Energy profile (molecular system, Gaussian09, SMD(CH_2Cl_2)/BP86/SDD(Rh,P)/6-31G(d,p)) for the 1,3-H shift reaction via oxidative cleavage / reductive coupling in **[1–(propene)]$^{+}$**.

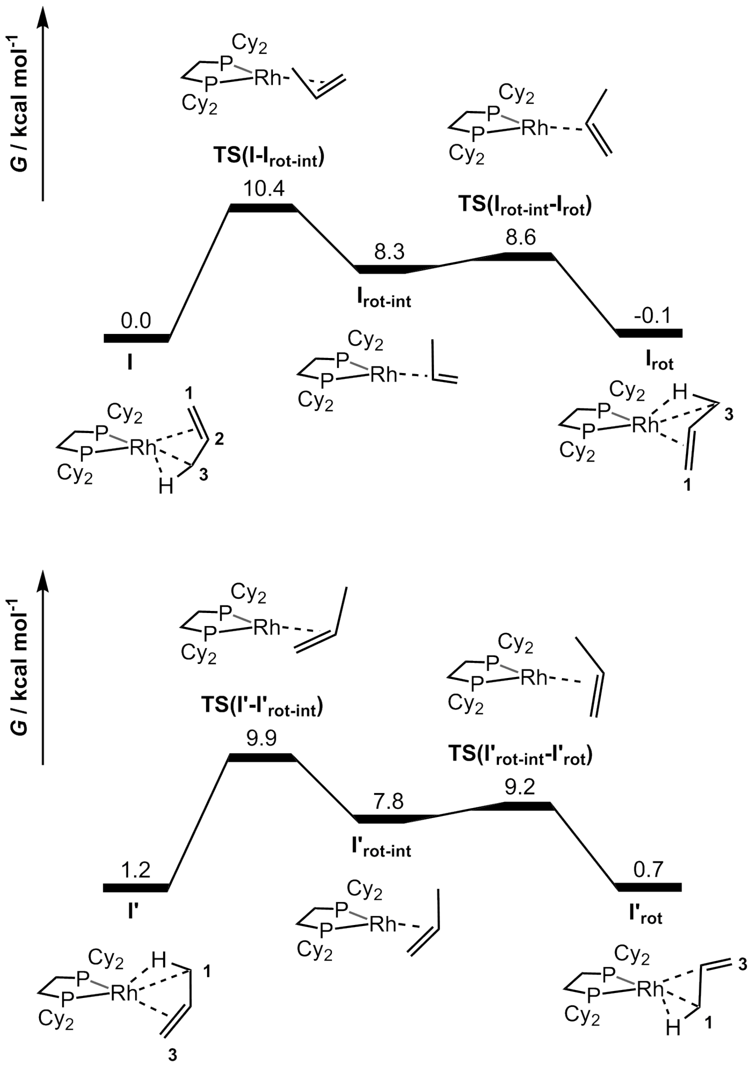

Figure S67: Calculated Gibbs Free Energy profile (periodic system) for the (anti)clockwise rotation of propene in **[1–(propene)][BAr^F$_4$]**.

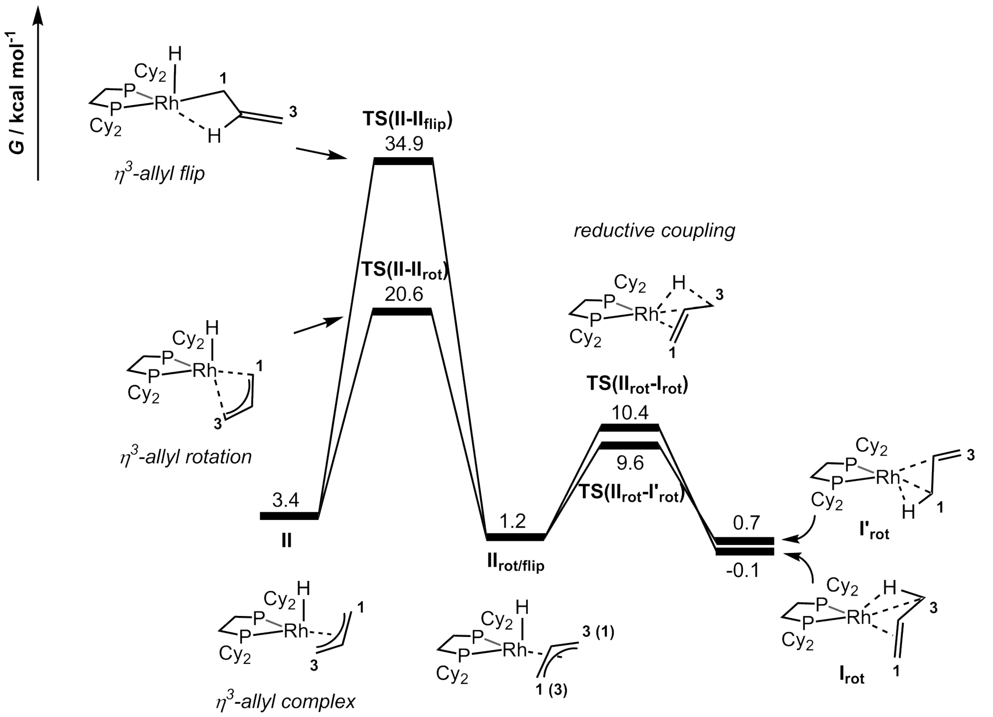

Figure S68: Calculated Gibbs Free Energy profile (periodic system) for the rearrangement of the allyl complex followed by reductive coupling in **[1–(propene)][BAr^F$_4$]**.

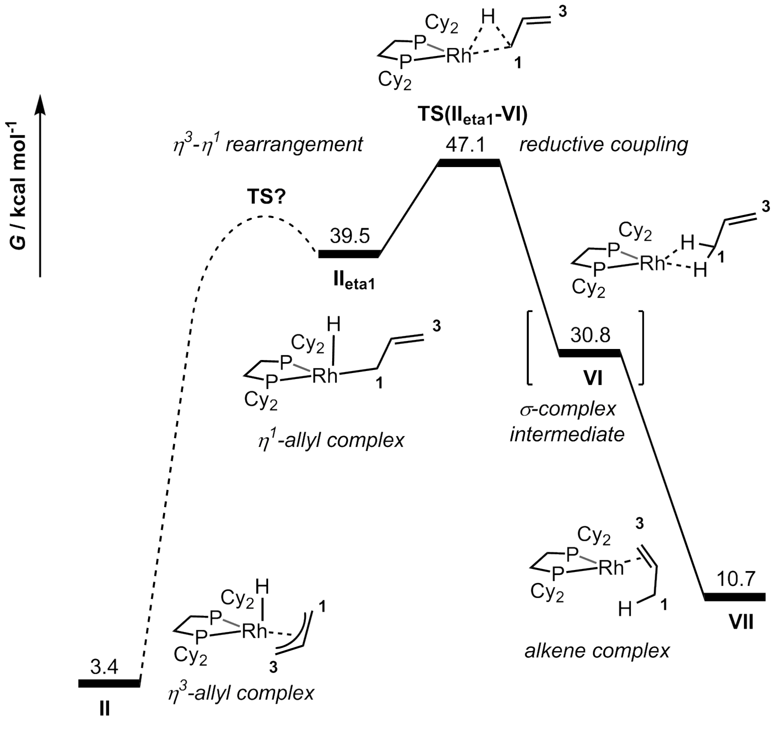

Figure S69: Calculated Gibbs Free Energy profile (periodic system) for the η^3-η^1-rearrangement of the allyl complex followed by reductive coupling in **[1–(propene)][BAr^F$_4$]**.

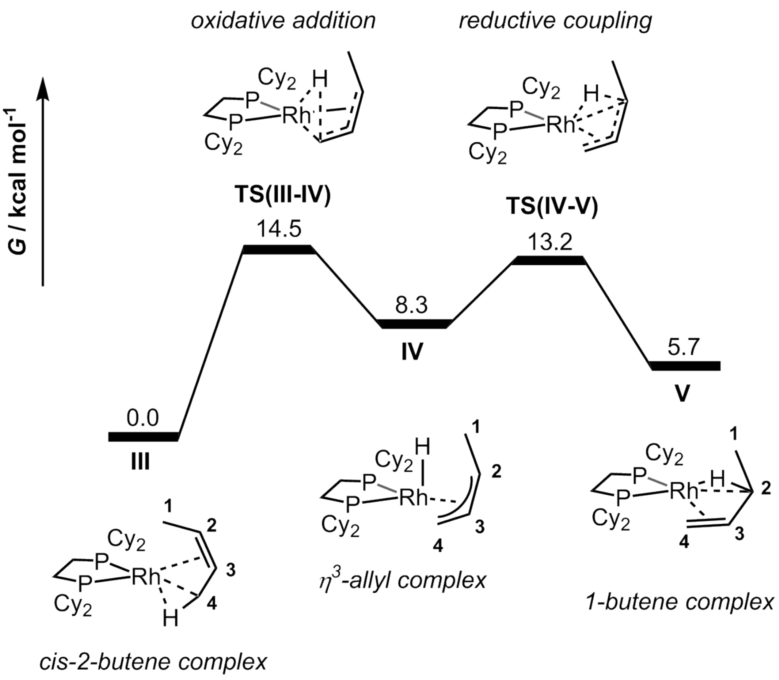

Figure S70: Calculated Gibbs Free Energy profile (periodic system) for the 1,3-H shift reaction via oxidative cleavage / reductive coupling in **[1–(butene)][BAr$^{F}_{4}$]**. The reaction in the direction from right to left corresponds to the isomerization of 1-butene to *cis*-2-butene.

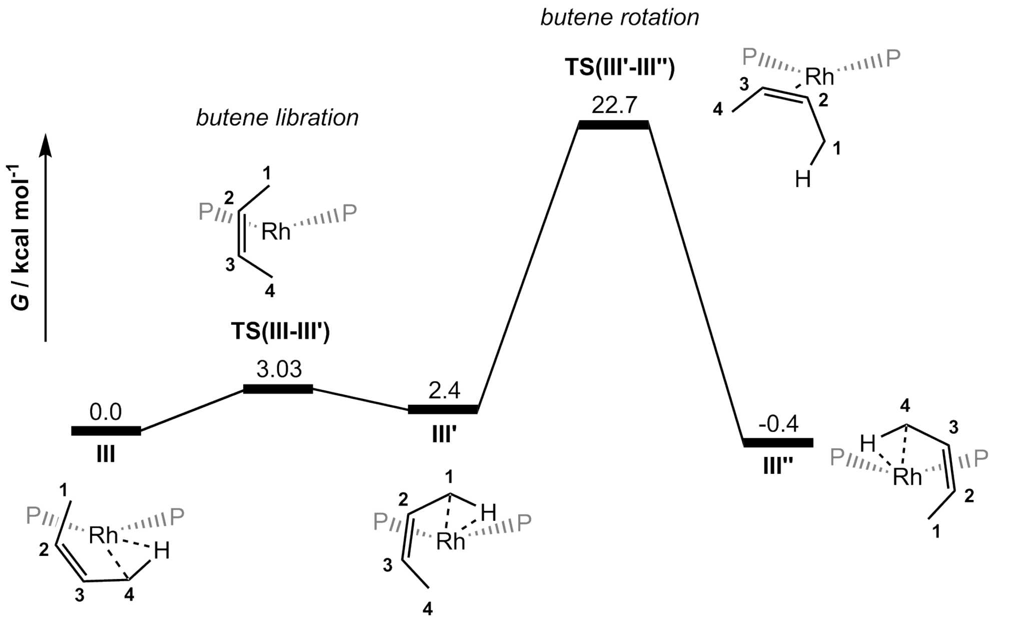

Figure S71: Calculated Gibbs Free Energy profile (periodic system) for the libration and rotation of *cis*-2-butene in **[1–(butene)][BAr$^{F}_{4}$]**.

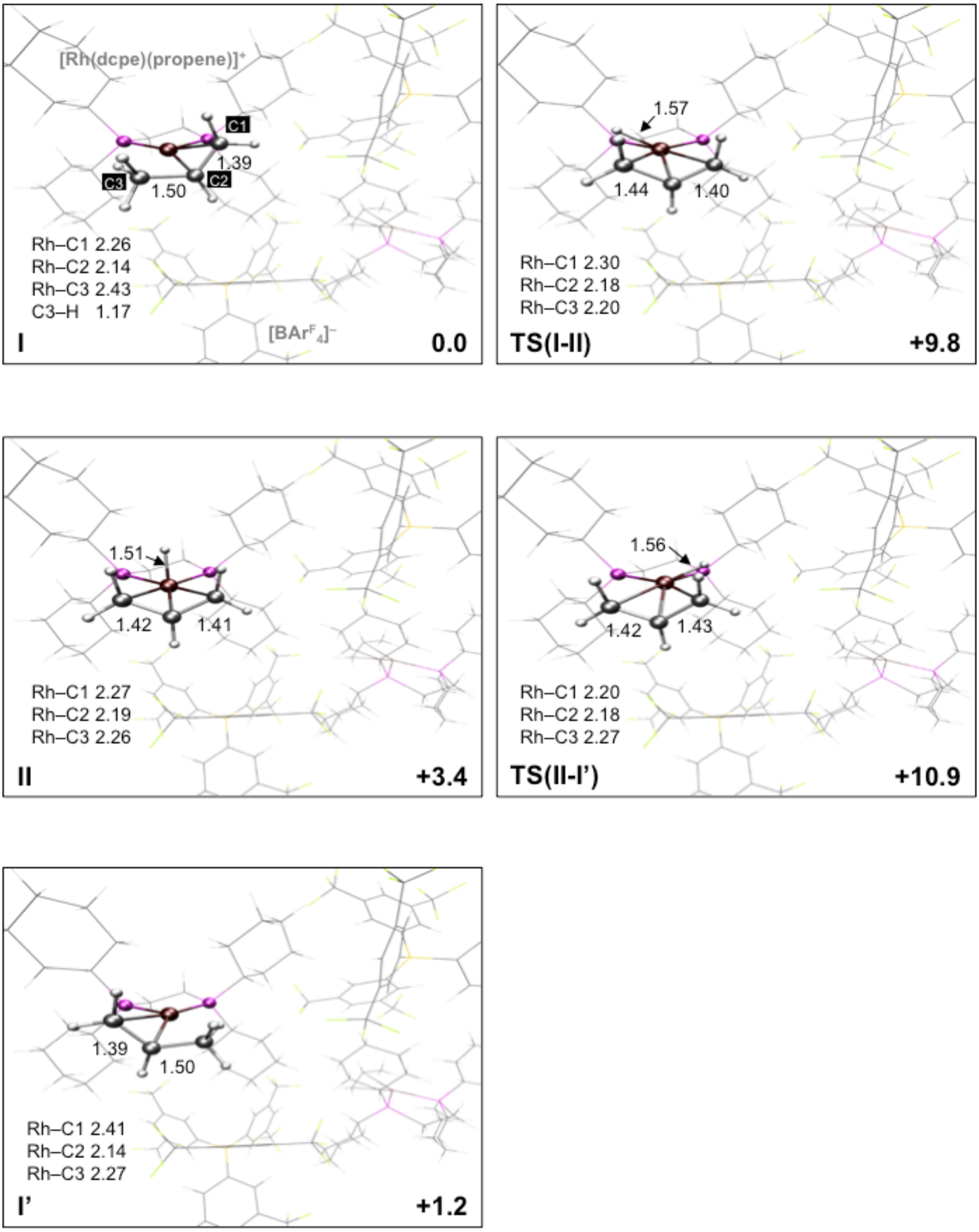

Figure S72: Optimized geometries (periodic system, bond distances in Å) along with Gibbs Free Energies (kcal mol^{-1}) for the 1,3-H shift reaction via oxidative cleavage / reductive coupling in **[1–(propene)][BAr$^F{}_4$]**.

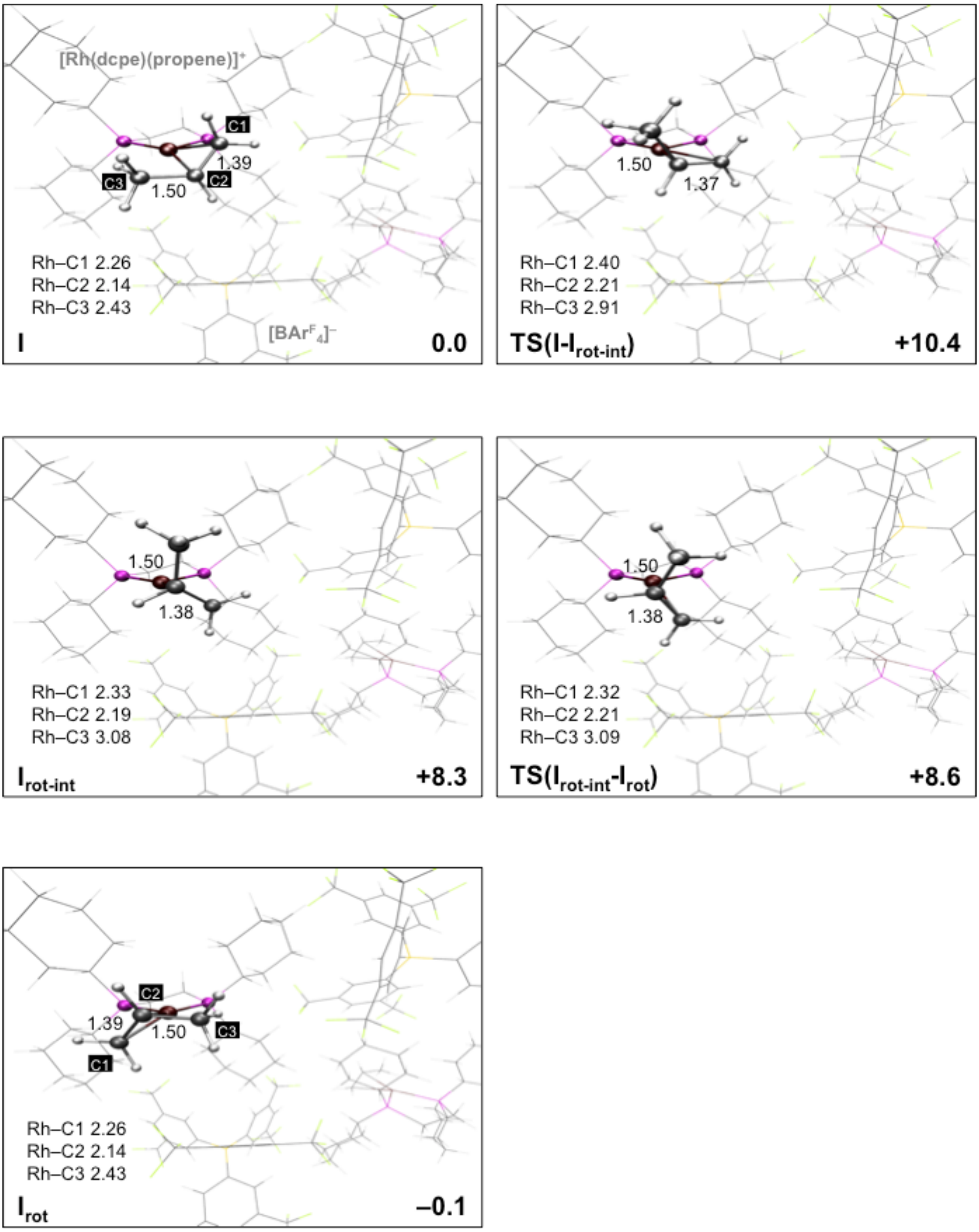

Figure S73: Optimized geometries (periodic system, bond distances in Å) along with Gibbs Free Energies (kcal mol^{-1}) for the clockwise rotation of propene in **[1–(propene)][BAr$^F_4$]**.

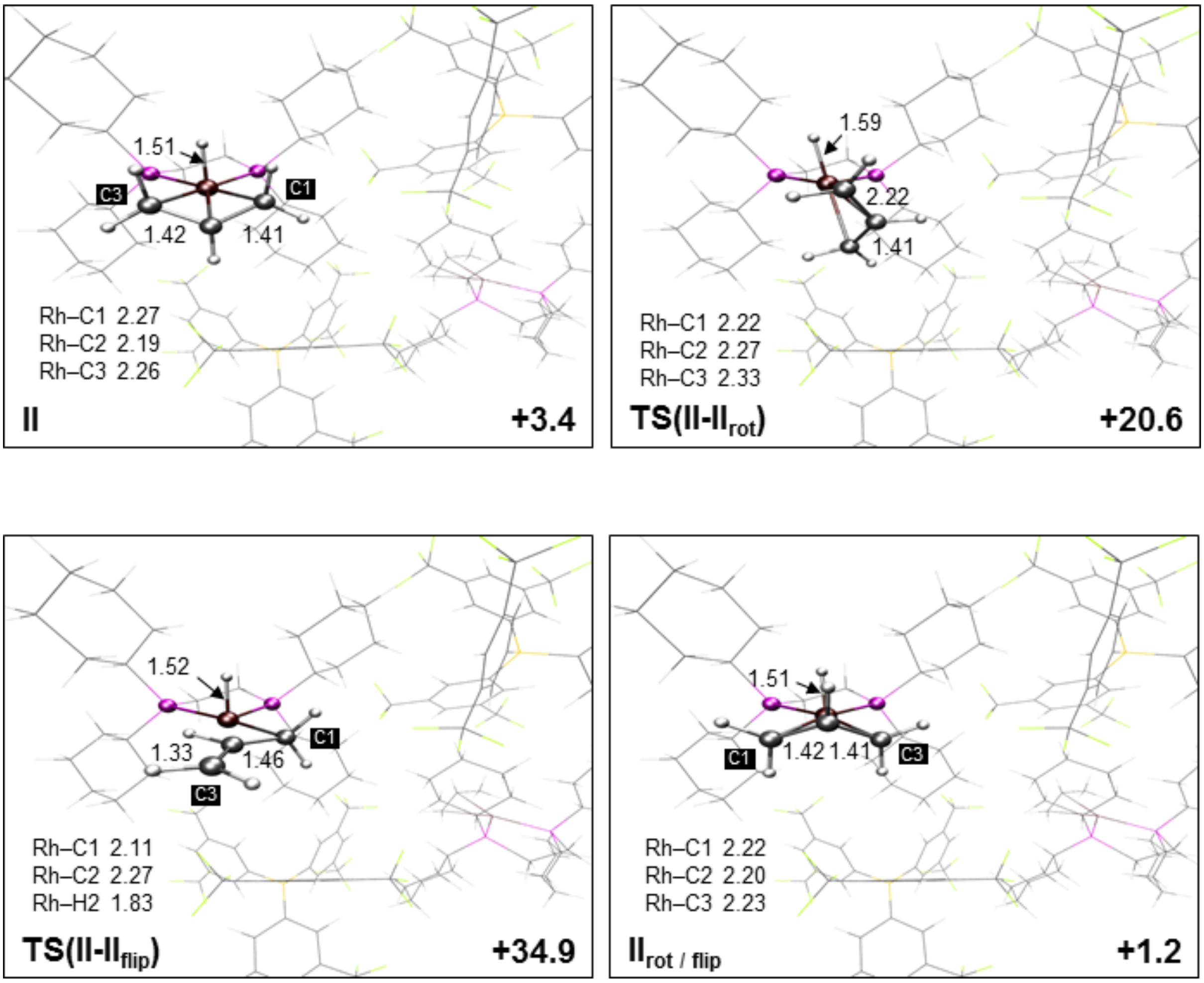

Figure S74: Optimized geometries (periodic system, bond distances in Å) along with Gibbs Free Energies (kcal mol^{-1}) for the rearrangement of the allyl complex in **[1–(propene)][BAr$^F{}_4$]**.

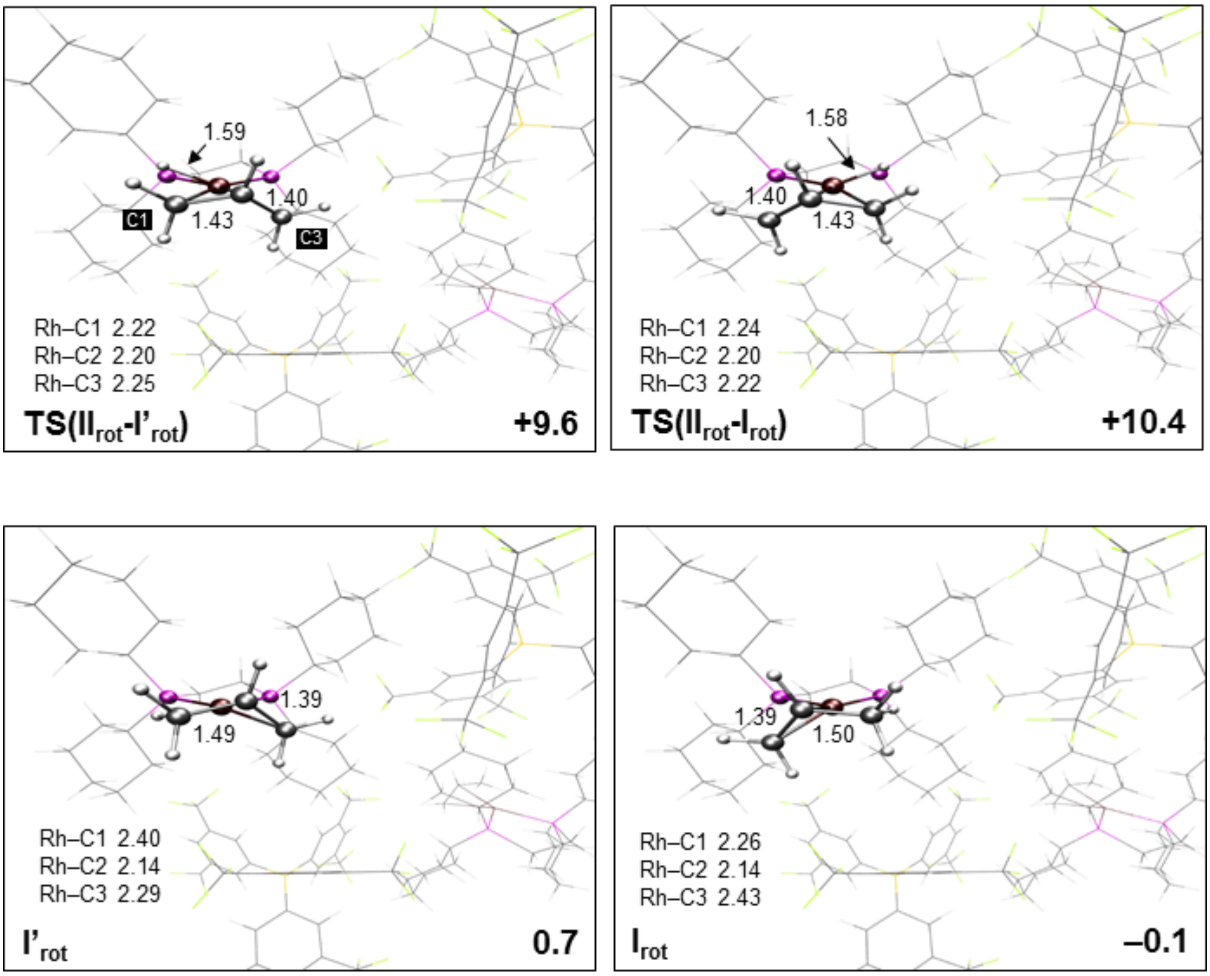

Figure S75: Optimized geometries (periodic system, bond distances in Å) along with Gibbs Free Energies (kcal mol^{-1}) for reductive coupling following allyl rearrangement in **[1–(propene)][BAr$^F_4$]**.

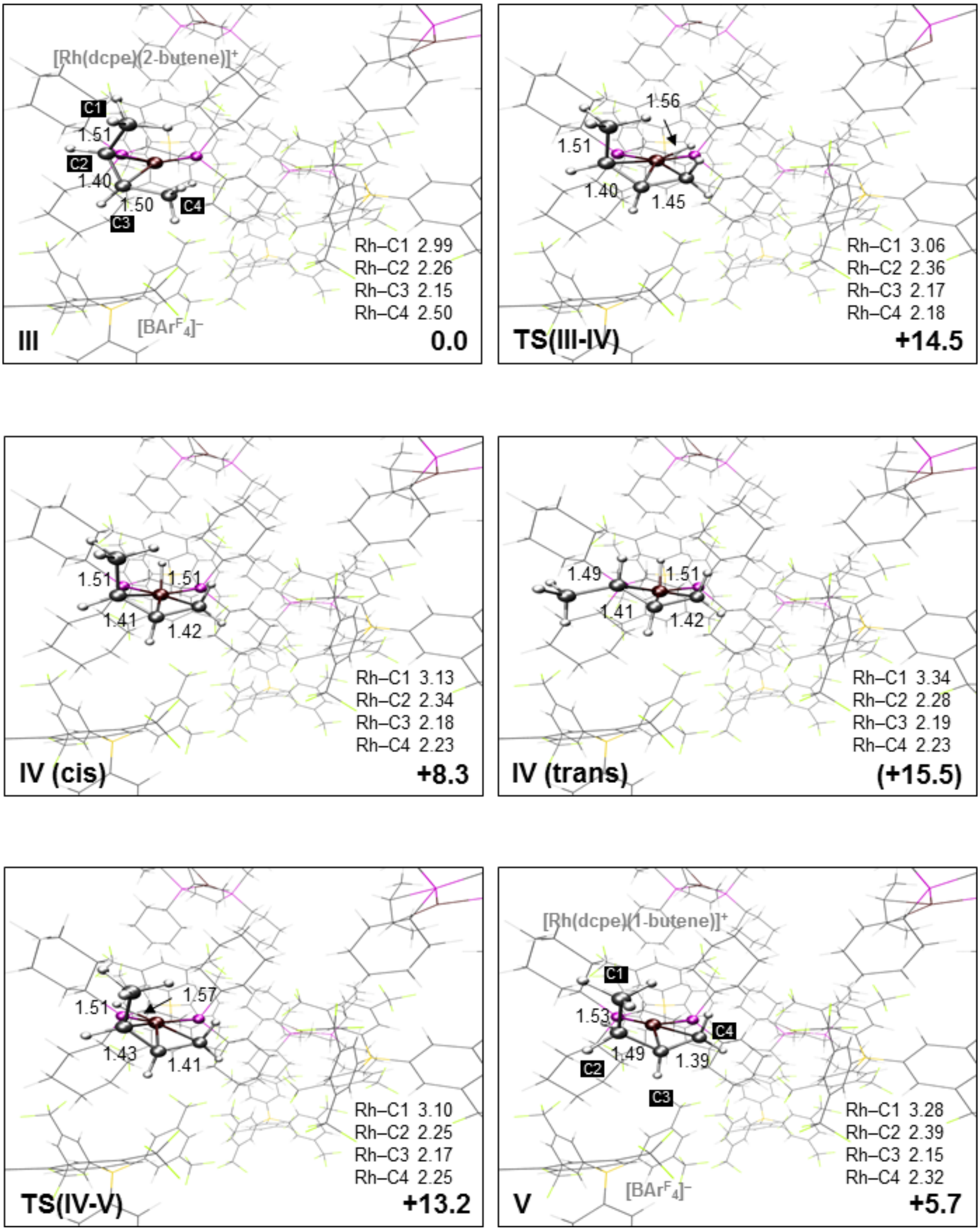

Figure S76: Optimized geometries (periodic system, bond distances in Å) along with Gibbs Free Energies (kcal mol^{-1}) for the 1,3-H shift reaction via oxidative cleavage / reductive coupling in **[1–(butene)][BAr$^F_4$]**.

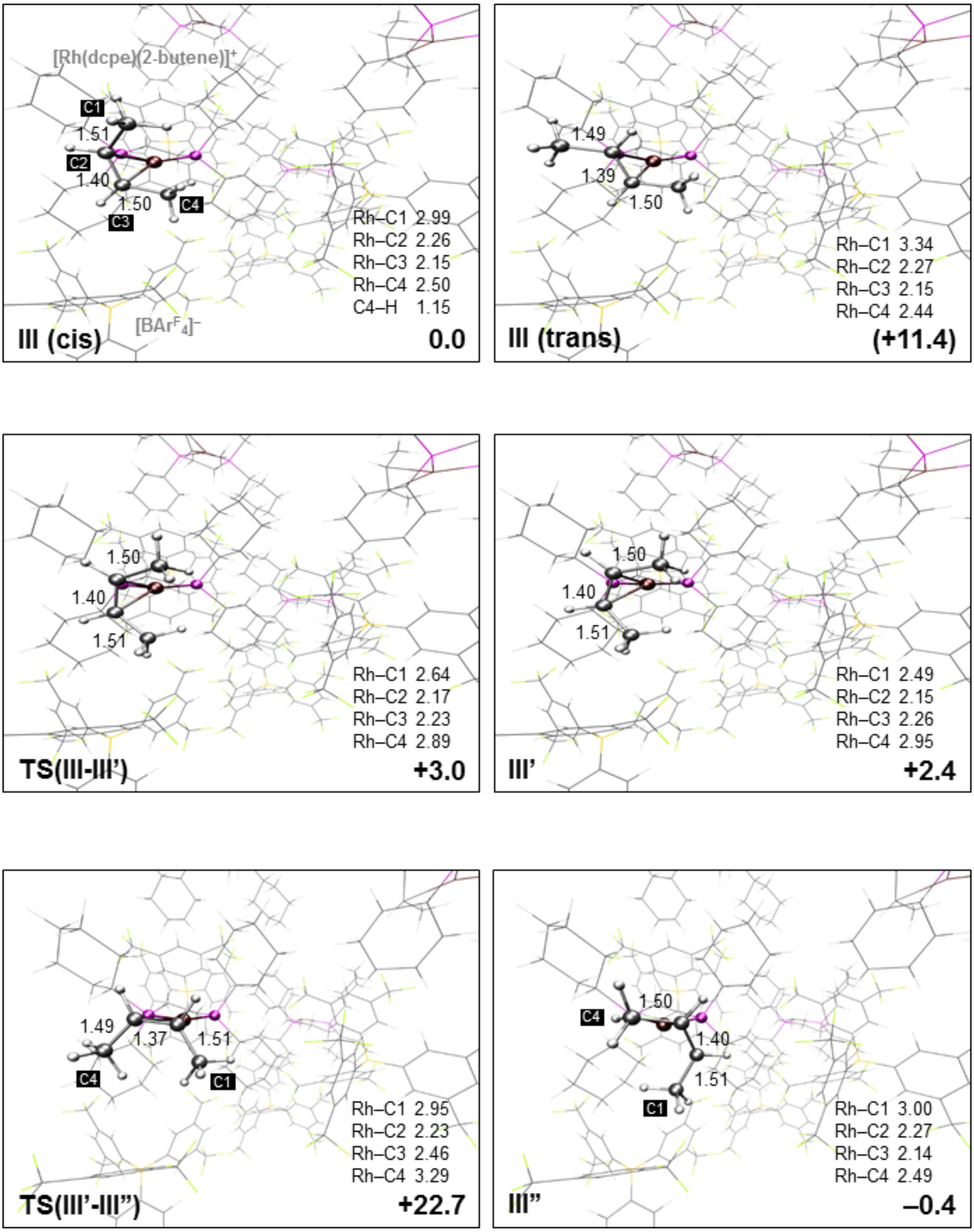

Figure S77: Optimized geometries (periodic system, bond distances in Å) along with Gibbs Free Energies (kcal mol^{-1}) for the libration and rotational process in **[1–(butene)][BAr$^F_4$]**.

S.6. References

S1 Pike, S. D.; Chadwick, F. M.; Rees, N. H.; Scott, M. P.; Weller, A. S.; Krämer, T.; Macgregor, S. A. *J. Am. Chem. Soc.* **2015**, *137*, 820.

S2 Peersen, O. B.; Wu, X. L.; Kustanovich, I.; Smith, S. O. *J. Magn. Reson.* **1993**, *104*, 334.

S3 Earl, W. L.; Vanderhart, D. L. *J. Magn. Reson.* **1982**, *48*, 35.

S4 Pike, S. D.; Krämer, T.; Rees, N. H.; Macgregor, S. A.; Weller, A. S. *Organometallics* **2015**, *34*, 1487.

S5 Lubben, A. T.; McIndoe, J. S.; Weller, A. S. *Organometallics* **2008**, *27*, 3303.

S6 Chadwick, F. M.; Rees, N. H.; Weller, A. S.; Krämer, T.; Iannuzzi, M.; Macgregor, S. A. *Angew. Chem. Int. Edit.* **2016**, *55*, 3677.

S7 Fulmer, G. R.; Miller, A. J. M.; Sherden, N. H.; Gottlieb, H. E.; Nudelman, A.; Stoltz, B. M.; Bercaw, J. E.; Goldberg, K. I. *Organometallics* **2010**, *29*, 2176.

S8 Cosier, J.; Glazer, A. M. *J. Appl. Crystallogr.* **1986**, *19*, 105.

S9 Otwinowski, Z.; Minor, W. *Methods in Enzymology* **1997**, *276*, 307.

S10 Oxford Diffraction Ltd., 2011.

S11 Palatinus, L.; Chapuis, G. *J. Appl. Crystallogr.* **2007**, *40*, 786.

S12 Sheldrick, G. M. *Acta Cryst. A* **2015**, *71*, 3.

S13 Betteridge, P. W.; Carruthers, J. R.; Cooper, R. I.; Prout, K.; Watkin, D. J. *J. Appl. Crystallogr.* **2003**, *36*, 1487.

S14 Cooper, R. I.; Thompson, A. L.; Watkin, D. J. *J. Appl. Crystallogr.* **2010**, *43*, 1100.

S15 Sheldrick, G. M. *Acta Crystallogr., Sect. A* **2008**, *64*, 112.

S16 Dolomanov, O. V.; Bourhis, L. J.; Gildea, R. J.; Howard, J. A. K.; Puschmann, H. *J. Appl. Cryst.* **2009**, *42*, 339.

S17 Spek, A. *J. Appl. Crystallogr.* **2003**, *36*, 7.

S18 VandeVondele, J.; Krack, M.; Mohamed, F.; Parrinello, M.; Chassaing, T.; Hutter, J. *Comput. Phys. Commun.* **2005**, *167*, 103.

S19 Hutter, J.; Iannuzzi, M.; Schiffmann, F.; VandeVondele, J. *Wires Comput. Mol. Sci.* **2014**, *4*, 15.

S20 VandeVondele, J.; Hutter, J. *J. Chem. Phys.* **2007**, *127*, 114105.

S21 Hartwigsen, C.; Goedecker, S.; Hutter, J. *Phys. Rev. B* **1998**, *58*, 3641.

S22 Goedecker, S.; Teter, M.; Hutter, J. *Phys. Rev. B* **1996**, *54*, 1703.

S23 Krack, M. *Theor. Chem. Acc.* **2005**, *114*, 145.

S24 Perdew, J. P.; Burke, K.; Ernzerhof, M. *Phys. Rev. Lett.* **1996**, *77*, 3865.

S25 Grimme, S.; Antony, J.; Ehrlich, S.; Krieg, H. *J. Chem. Phys.* **2010**, *132*, 154104.

S26 Henkelman, G.; Jonsson, H. *J. Chem. Phys.* **1999**, *111*, 7010.

S27 Henkelman, G.; Uberuaga, B. P.; Jonsson, H. *J. Chem. Phys.* **2000**, *113*, 9901.

S28 Ghysels, A.; Verstraelen, T.; Hemelsoet, K.; Waroquier, M.; Van Speybroeck, V. *J. Chem. Inf. Model.* **2010**, *50*, 1736.

S29 Gaussian 09.

S30 Marenich, A. V.; Cramer, C. J.; Truhlar, D. G. *J. Phys. Chem. B* **2009**, *113*, 6378.

S31 Pickard, C. J.; Mauri, F. *Phys. Rev. B* **2001**, *63*, 245101.

S32 Yates, J. R.; Pickard, C. J.; Mauri, F. *Phys. Rev. B* **2007**, *76*, 024401.

S33 Clark, S. J.; Segall, M. D.; Pickard, C. J.; Hasnip, P. J.; Probert, M. J.; Refson, K.; Payne, M. C. *Z. Kristallogr.* **2005**, *220*, 567.

S34 Monkhorst, H. J.; Pack, J. D. *Phys. Rev. B* **1976**, *13*, 5188.
S35 Chadwick, F. M.; Krämer, T.; Gutmann, T.; Rees, N. H.; Thompson, A. L.; Edwards, A. J.; Buntkowsky, G.; Macgregor, S. A.; Weller, A. S. *J. Am. Chem. Soc.* **2016**, *138*, 13369.
S36 van Lenthe, E.; Baerends, E. J.; Snijders, J. G. *J. Chem. Phys.* **1993**, *99*, 4597.
S37 van Lenthe, E.; Baerends, E. J.; Snijders, J. G. *J. Chem. Phys.* **1994**, *101*, 9783.
S38 Lodewyk, M. W.; Siebert, M. R.; Tantillo, D. J. *Chem. Rev.* **2012**, *112*, 1839.
